# Amyloidosis is associated with thicker myelin and increased oligodendrogenesis in the adult mouse brain

**DOI:** 10.1002/jnr.24672

**Published:** 2020-06-18

**Authors:** Solène Ferreira, Kimberley A. Pitman, Shiwei Wang, Benjamin S. Summers, Nicole Bye, Kaylene M. Young, Carlie L. Cullen

**Affiliations:** ^1^ Menzies Institute for Medical Research University of Tasmania Hobart Tasmania Australia; ^2^ School of Medicine University of Tasmania Hobart Tasmania Australia

**Keywords:** Alzheimer's disease, amyloid, dementia, myelin, oligodendrocyte, RRID:AB_91939, RRID:AB_476692, RRID:AB_570666, RRID:AB_662798, RRID:AB_162542, RRID:AB_2040202, RRID:AB_2236897, RRID:AB_2493179, RRID:AB_2534017, RRID:AB_2534117, RRID:AB_2535792, RRID:AB_2535794, RRID:AB_2535864, RRID:AB_2536183, RRID:AB_2617137, RRID:AB_2827931, RRID:AB_10013361, RRID:AB_10806491, RRID:IMSR_JAX:006148, RRID:IMSR_JAX:007669, RRID:IMSR_JAX:008169, RRID:MMRRC_034836‐JAX, RRID:SCR_000441, RRID:SCR_002798, RRID:SCR_003070, RRID:SCR_011323

## Abstract

In Alzheimer's disease, amyloid plaque formation is associated with the focal death of oligodendrocytes and soluble amyloid β impairs the survival of oligodendrocytes in vitro. However, the response of oligodendrocyte progenitor cells (OPCs) to early amyloid pathology remains unclear. To explore this, we performed a histological, electrophysiological, and behavioral characterization of transgenic mice expressing a pathological form of human *amyloid precursor protein* (*APP*), containing three single point mutations associated with the development of familial Alzheimer's disease (*PDGFB‐APP^Sw.Ind^*, also known as J20 mice). *PDGFB‐APP^Sw.Ind^* transgenic mice had impaired survival from weaning, were hyperactive by 2 months of age, and developed amyloid plaques by 6 months of age, however, their spatial memory remained intact over this time course. Hippocampal OPC density was normal in P60‐P180 *PDGFB‐APP^Sw.Ind^* transgenic mice and, by performing whole‐cell patch‐clamp electrophysiology, we found that their membrane properties, including their response to kainate (100 µM), were largely normal. However, by P100, the response of hippocampal OPCs to GABA was elevated in *PDGFB‐APP^Sw.Ind^* transgenic mice. We also found that the nodes of Ranvier were shorter, the paranodes longer, and the myelin thicker for hippocampal axons in young adult *PDGFB‐APP^Sw.Ind^* transgenic mice compared with wildtype littermates. Additionally, oligodendrogenesis was normal in young adulthood, but increased in the hippocampus, entorhinal cortex, and fimbria of *PDGFB‐APP^Sw.Ind^* transgenic mice as pathology developed. As the new oligodendrocytes were not associated with a change in total oligodendrocyte number, these cells are likely required for cell replacement.

## Graphical abstract

Myelin structure is altered in the hippocampus of mice overexpressing a pathological variant of the human amyloid precursor protein (PDGFB‐APP^Sw.Ind^; *APP mice*). *APP* mice have longer paranodes, thicker myelin, and shorter nodes of Ranvier than wildtype mice by 3 months of age, however, total axon density and the proportion of axons that were myelinated was normal. Between 4 and 6 months of age, *APP* mice have more new oligodendrocytes added to the hippocampus, entorhinal cortex, and fimbria, while total oligodendrocyte and oligodendrocyte progenitor cell density remained stable, suggesting that new oligodendrocytes may be produced to replace oligodendrocytes lost due to amyloid pathology.

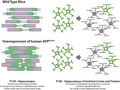

**Significance**

Alzheimer's disease is the leading cause of dementia and presents growing social and economic challenges. Once initiated, Alzheimer's disease can affect all cell types in the brain and treating this condition will likely require early detection and a combination therapy to halt disease progression. This study has determined that early amyloid pathology affects immature brain cells called oligodendrocyte progenitor cells (OPCs) and mature brain cells called oligodendrocytes, but does not prevent OPCs from producing new cells. Our findings suggest that OPCs are a possible target to protect against early neurodegeneration in Alzheimer's disease.

## 1 INTRODUCTION

Alzheimer's disease is a progressive neurodegenerative disease characterized post‐mortem by the presence of extracellular plaques of aggregated amyloid β (Miller et al., 1993; Roher et al., 1993; Burgold et al., 2011; reviewed by Selkoe & Hardy, 2016) and neurofibrillary tangles, formed by the intracellular aggregation of cytoskeletal proteins, primarily hyperphosphorylated tau (Braak & Braak, 1996; Goedert, Spillantini, Jakes, Rutherford, & Crowther, 1989; Iseki et al., 2006; Schmidt, Lee, & Trojanowski, 1990). In normal human aging, white matter degeneration occurs in brain regions critical for cognitive and emotional processing, including the hippocampus, neocortex, and frontal white matter tracts, and the level of white matter degeneration correlates with declining information processing speeds and developing cognitive impairment (Charlton et al., 2006; Chopra et al., 2018; Fan et al., 2019; Hirsiger et al., 2017). However, in Alzheimer's disease white matter degeneration is exacerbated (Benitez et al., 2014; Brueggen et al., 2019; Choi, Lim, Monteiro, & Reisberg, 2005; O'Dwyer et al., 2011; Stricker et al., 2009; Zhang, Schuff et al., 2009), and oligodendrocyte loss and demyelination have been detected at sites of pathological damage in the gray and white matter, post‐mortem (Behrendt et al., 2013; Mitew et al., 2010; Tse, Cheng, Ma, & Herrup, 2018).

White matter degeneration may occur early in human Alzheimer's disease pathology. Diffusion tensor imaging (DTI) studies, examining individuals in the preclinical stages of disease, determined that measures of fractional anisotropy increased and measures of mean diffusivity decreased in white matter regions such as the fornix, cingulum, and corpus callosum, and changes in these DTI parameters correlated with amyloid β1‐42 load (Gold et al., 2014; Racine et al., 2014; Shi, Zhao, Wong, Wang, & Mok, 2015). Furthermore, in preclinical individuals carrying genetic mutations that increase their risk of developing Alzheimer's disease, a lower cerebral spinal fluid concentration of amyloid β1‐42, indicative of increased amyloid plaque deposition (Grimmer et al., 2009), correlated with more severe white matter hyperintensities (Lee et al., 2016; Scott et al., 2015).

*In vitro*, rodent oligodendrocyte and oligodendrocyte progenitor cell (OPC) function is impaired by amyloid proteins. The exposure of cultured rat oligodendrocytes to amyloid β1‐42 or amyloid β25‐35 resulted in oxidative stress and cell death (Lee et al., 2004; Xu et al., 2001), and exposure to amyloid β1‐42 impaired myelin sheath formation (Horiuchi et al., 2012). Additionally, exposure of a mouse OPC line (mOP) to amyloid β1‐42 induced cell death of both differentiated and undifferentiated cells (Desai, Guercio, Narrow, & Bowers, 2011). The influence of amyloid pathology on OPC and oligodendrocyte health has also been examined in vivo, in mice carrying human pathological variants of *amyloid precursor protein (APP)* and *PSEN1* (Desai et al., 2011). In *APP/PSEN1* transgenic mice, amyloid plaques formed by 2 months of age (Radde et al., 2006), and expression of the OPC proteoglycan NG2 and the oligodendrocyte proteins CNPase and MBP increased in the hippocampus (Wu et al., 2017). At 6 months of age, despite an increase in OPC density and proliferation and increased newborn oligodendrocyte density, focal demyelination was detected in association with amyloid plaques, and myelin aberrations were apparent including double ensheathment, excess cytoplasm in the inner loop, myelin out‐folding, degenerating sheaths, and myelin ballooning (Behrendt et al., 2013). However, in these mice it is difficult to attribute specific changes in cells of the oligodendrocyte lineage with *APP* overexpression or amyloidosis, as PSEN1 modulates Notch signaling (Newman et al., 2014), and can directly impact oligodendrocyte maturation and myelination (Zhang, Tadesse et al., 2009). In triple transgenic (3xTg) mice that carry human pathological variants in *APP^Sw^*, *PSEN1^M146V^*, and *MAPT^P301L^*, myelin protein expression was reduced in the hippocampal CA1 at 2 months of age and Schaffer collateral axons were dystrophic and had granulated myelin (Desai et al., 2009), such that fewer myelinated CA1 axons were present by 6 months of age (Desai et al., 2010). Although the expression of amyloid β1‐42 was not increased until 6 months of age in the 3xTg mice (Desai et al., 2010), the viral delivery of intracellular targeted anti‐amyloid β antibodies at 2 months of age, to prevent amyloid β aggregation, resulted in normal myelination at 6 months (Desai et al., 2010), suggesting that amyloid pathology was the primary driver of oligodendrocyte damage in these mice.

We aimed to determine whether pathological *APP* signaling, in the absence of pathological *PSEN1* or *MAPT*, was sufficient to influence oligodendrocyte lineage cell function in the hippocampus, fimbria, or entorhinal cortex in early stages of disease. The hippocampus and entorhinal cortex are brain regions affected early in Alzheimer's disease (Du et al., 2001; Pennanen et al., 2004), and the fimbria is the major white matter tract that connects the hippocampal hemispheres to subcortical and cortical regions such as the thalamus and prefrontal cortex (Jin & Maren, 2015; Wyss, Swanson, & Cowan, 1980). Herein, we show that mice carrying the Swedish and Indiana mutations in *APP* (*PDGFB‐APP^Sw.Ind^* transgenic mice) maintain a normal density of OPCs and oligodendrocytes in the hippocampus from P60 to P180, however, OPC behavior is altered by amyloid pathology. In young adulthood (P100), OPCs in the hippocampus of *PDGFB‐APP^Sw.Ind^* transgenic mice have an increased response to GABA, displaying larger currents upon bath application of the neurotransmitter. Oligodendrocyte maturation also appears to be affected in the hippocampus of these mice, as the nodes of Ranvier are shorter and the paranodes longer, and this phenotype is associated with increased myelin thickness by P100. The number of new oligodendrocytes produced by adult OPCs was normal in early adulthood, but increased in the hippocampus, entorhinal cortex, and fimbria of *PDGFB‐APP^Sw.Ind^* transgenic mice as amyloid pathology developed. As total oligodendrocyte density was unchanged by P180, it is likely that the newborn oligodendrocytes replace oligodendrocytes lost to pathology.

## 2 METHODS

### 2.1 Experimental subjects

Male and female mice were used for these experiments; *n* = 238 mice were assigned to experimental cohorts, including *n* = 29 *APP* mice that were assigned but died prior to the required timepoint, preventing analysis. Experimental mouse numbers did not allow testing of effects between genders. *Rosa26‐YFP* cre‐sensitive reporter mice (Srinivas et al., 2001) were purchased from the Jackson Laboratory [B6.129X1‐Gt(ROSA)26Sortm1(EYFP)Cos/J, stock #006148; RRID:IMSR_JAX:006148] and backcrossed onto a C57BL/6 background in house for >10 generations. *PDGFB‐APP^Sw,Ind^* mice overexpressing human APP with mutations predisposing to Alzheimer's disease (referred to here as *APP* mice, but also known as J20 mice; Mucke et al., 2000) were purchased from the Jackson Laboratory [B6.Cg‐Zbtb20Tg(PDGFB‐APPSwInd)20Lms/2Mmjax, stock #006293; RRID:MMRRC_034836‐JAX] and backcrossed onto a C57BL/6 background in house for > 20 generations. *Prnp‐MAPT^P301S^* (*MAPT*) transgenic mice (Ferreira et al., 2020; Yoshiyama et al., 2007), that express a human variant of *MAPT* were purchased from the Jackson Laboratory [B6;C3‐Tg(Prnp‐MAPT*P301S)PS19Vle/J, stock #008169; RRID:IMSR_JAX:008169] and backcrossed onto a C57BL/6 background for >20 generations. *Pdgfrα‐CreER^T2^* transgenic mice (Rivers et al., 2008) were previously generated in the laboratory of Prof. William D Richardson (University College London, UK; no RRID available). *Pdgfrα–H2BGFP* mice (Hamilton, Klinghoffer, Corrin, & Soriano, 2003) were purchased from the Jackson Laboratory [B6.129S4‐Pdgfratm11(EGFP)Sor/J, stock # 007669; RRID:IMSR_JAX:007669] and backcrossed onto a C57BL/6 background for >20 generations. Mice were maintained on a C57BL/6 background and bred to generate experimental mice that were heterozygous for each transgene. Male and female littermates were weaned >P35 and housed in individually ventilated cages (Optimice) at 21°C, on a 12h light/dark cycle (07:00–19:00) with food and water available *ad libitum*.

### 2.2 Ethical approval

All animal experiments were approved by the Animal Ethics Committee of the University of Tasmania (13741 and 16151) and carried out in accordance with the Australian code of practice for the care and use of animals in science. Details of animal experiments are reported in accordance with the ARRIVE guidelines.

### 2.3 Experimental design and procedures

The source for all materials used in this study is clearly identified in text or in Table S1

#### 2.3.1 Genotyping

*Pdgfrα‐H2BGFP* transgene expression was determined by detecting GFP expression upon light illumination of the head with a BlueStar flashlight (Nightsea, Lexington USA) at P1‐P2. *Cre recombinase* and *Rosa26‐YFP* transgene expression was evaluated by polymerase chain reaction (PCR) of genomic DNA extracted from ear biopsies as previously described (O'Rourke et al., 2016). *MAPT* and *APP* transgenes were also detected by PCR using Taq DNA polymerase with a standard magnesium‐free Taq buffer (M0329L; New England BioLabs), the deoxynucleotide (dNTP) solution mix (N0447L; New England BioLabs), and the following primers: MAPT 5′ GGG GAC ACG TCT CCA CGG CAT CTC AGC AAT GTC TCC and MAPT 3′ TCC CCC AGC CTA GAC CAC GAG AAT, or APP 5′ GGT GAG TTT GTA AGT GAT GCC and APP 3′ TCT TCT TCT TCC ACC TCA GC. Each reaction was heated to 94°C for 4 min and amplified across 35 cycles of 94°C for 30 s, 57°C for 45 s, and 72°C for 60 s, followed by a final 10 min at 72°C, to yield DNA fragments of ~350 bp and ~360 bp, respectively. *MAPT* and *APP* PCR products were run on a 2% (w/v) agarose gel in TAE containing SYBR‐safe (Thermo Fisher Scientific) and visualized using an Amersham Imager 600 (GE Healthcare Life Sciences, UK).

#### 2.3.2 Tamoxifen preparation and delivery

Control and *APP* mice carrying the *Pdgfrα‐CreER^T2^* and *Rosa26‐YFP* transgenes were used for all lineage tracing studies. To activate Cre recombinase and enable expression of the yellow fluorescent reporter, Tamoxifen (Sigma) was dissolved in corn oil (40 mg/ml) by sonication (Ultrasonic cleaner FXP 8M, Unisonics Australia) at 21°C for 2 hr. Adult mice (P60) received 300 mg tamoxifen/kg body weight daily for four consecutive days by oral gavage (as per O'Rourke et al., 2016). Mice were analyzed 7, 60, 90, or 120 days after their first dose of Tamoxifen, and are referred to as P60 + 7, P60 + 60, P60 + 90, and P60 + 120, respectively.

#### 2.3.3 Western blot

Mice were terminally anesthetized using sodium pentobarbital (i.p 100 mg/kg) and transcardially perfused with ice‐cold 0.01 M phosphate‐buffered saline (PBS). On ice, the dorsal region of the hippocampus was collected from 1 mm thick coronal slices spanning ~Bregma −1.06 to −2.18 (Franklin & Paxinos, 2007), and prepared for analysis by Western blot as per Auderset, Cullen, and Young (2016). Briefly, the SeeBlue Plus2 Pre‐Stained Protein Standard (Novex, Life Technologies) and lysates were run on precast Bolt™ 4%–12% Bis‐Tris Plus gels (Life Technologies, Australia) and transferred onto ethanol‐activated PVDF membranes (BioRad). Each membrane was blocked for 1 hr at 21°C by immersion in 0.2% (v/v) Tween‐20 in Tris‐Buffered Saline (TBS‐T) containing 5% (w/v) skim milk powder, before being incubated with mouse anti‐6E10 (1:500, Covance; detects the C‐terminal of human APP; RRID:AB_662798) diluted in TBS‐T containing 5% (w/v) skim milk powder, overnight at 4°C. Each membrane was washed thrice in TBS‐T before being incubated with goat anti‐mouse HRP (1:10,000, Dako; RRID:AB_2617137) diluted in TBS‐T containing 1% (w/v) skim milk powder. After washing in TBS‐T the membrane was exposed to a 1:1 mix of Immobilon Western™ HRP Peroxidase Solution (Millipore) and Luminol Reagent (Millipore) to visualize the protein bands on an Amersham Imager 600 (GE Healthcare Life Sciences, UK). To control for protein loading, membranes were stripped by washing with PBS, TBS‐T, and blot stripping buffer (ThermoScientific), before incubating with mouse anti‐β‐actin (1:1,000, Sigma; RRID:AB_476692) diluted in TBS‐T containing 5% (w/v) skim milk. After washing, secondary goat anti‐mouse HRP (1:10,000, Dako; RRID:AB_2617137) was applied and the protein bands and visualized as previously described. Human APP or amyloid β oligomer expression was calculated by measuring the integrated density of the APP protein band at ~100 kDa or the amyloid β oligomer band at ~12 kDa (Collins, King, Woodhouse, Kirkcaldie, & Vickers, 2015) and normalizing the signal to β‐actin protein (~42 kDa) expression levels for each sample.

#### 2.3.4 Locomotor and cognitive testing

Behavioral testing was carried out on wildtype (WT) and *APP* transgenic littermates at P60, P90, or P180. Individual mice were tested once, and separate cohorts analyzed at each age. All behavioral testing was carried out during the dark phase of the light–dark cycle. Mice were moved to the testing room 2 hr prior to the light cycle change and habituated to the room for 3 hr. All testing was carried out within the same 5‐hr window of the dark phase. Sodium lights were used in the room and bright lights were used above the maze as needed. All trials were video recorded and animal movement tracked using automated tracking software (EthoVision XT 11, Noldus, Netherlands; RRID:SCR_000441). Male mice were tested prior to female mice, but the order of testing was otherwise randomized for each test. All equipment was cleaned with 70% ethanol between trials.

##### T‐Maze

The T‐Maze was performed using a protocol adapted from Deacon and Rawlins (2006). A mouse was placed in the start arm and once they chose to explore the left or right arm of the maze, retreat from that arm was blocked for 1 min. The mouse was then returned to the start arm and allowed to make another choice. This was repeated 10 times. Mice naturally exhibit exploratory behavior and tend to choose the arm not visited in the previous trial, therefore, returning to the same arm in successive trials was recorded as an error. The number of trials in which the mouse failed to alternate was recorded and is expressed as a proportion (%) of the number of trials [(errors/total trial number) × 100]. If a mouse failed to complete the trial (e.g., did not leave the start arm), data from that mouse were excluded from analysis.

##### Open field

The open field assessment was performed using a protocol adapted from Wang et al. (2013), to assess locomotor and anxiety‐like behavior. Each mouse was placed in an open square arena (30 cm^2^, with walls that were 20 cm in height) lit (200 lux) to create a bright center and dark perimeter, and the speed of movement and total distance moved was measured over a 10 min period.

##### Barnes maze

Mice underwent a shortened version of the Barnes maze protocol, adapted from Attar et al. (2013). On day 1, mice were placed in the brightly lit center (120 lux) of an elevated (30 cm above the ground), circular maze (100 cm diameter) that contained 20 holes evenly spaced around the circumference. After 1 min, the mice were gently directed to the escape box located underneath one of the holes in the circumference and left to habituate to the box for 5 min. On days 2 and 3, the maze was raised to 70 cm, and light intensity in the center of the maze increased to 160 lux. Distinct patterns were placed on each wall surrounding the maze, acting as spatial reference points that remained consistent throughout all trials. At the start of each trial, the mouse was placed at the center of the maze under a covered start box for 15–30 s before the box was removed, and the mouse left to explore until it found the escape box or 5 min elapsed. If a mouse did not find the escape box prior to the end of the trial, it was given direction to the box and allowed to enter it. After entering the escape box, each mouse was left for 1 min before being returned to the home cage to await the next trial. Mice were trained to learn the location of the escape box across three trials per day with an inter‐trial interval of 30–45 min. During training, approaching any hole that did not lead to the escape box was considered a primary error, and the number of primary errors made during a trial was measured as an indicator of learning (reviewed by Gawel, Gibula, Marszalek‐Grabska, Filarowska, & Kotlinska, 2019).

Short‐term and long‐term memory were assessed 1 day and 2 weeks after the initial training, respectively. For each memory probe trial, mice were returned to the maze with the escape box removed and were left to explore the maze for 5 min. The maze was divided into four quadrants within the tracking software (EthoVision XT 11) and the quadrant containing the hole that previously led to the escape box was designated the target zone. The proportion of time spent within the target zone during the probe trial was measured as an indicator of intact memory for the location of the escape box.

#### 2.3.5 Electrophysiology

Control, *MAPT,* and *APP* mice carrying the *Pdgfrα–H2BGFP* transgene, in which OPCs express GFP, were used for the electrophysiological characterization of OPCs. Following cervical dislocation, P30 (P30‐P35) and P100 (P100‐P114) mice were decapitated and their brains transferred into ice‐cold slicing solution (124 mM NaCl, 26 mM NaHCO_3_, 1 mM NaH_2_PO_4_, 2.5 mM KCl, 2 mM MgCl_2_, 2.5 mM CaCl_2_, 10 mM glucose, and 1 mM Na‐kynurenate) saturated with 95% O_2_/5% CO_2_. Horizontal brain slices (300 µm), prepared using a VT1200s vibratome (Leica), were incubated at 21°C in slicing solution that lacked Na‐kynurenate. Whole‐cell patch‐clamp recordings were made at 21°C from GFP^+^ cells situated among the Schaffer collaterals in CA1 of the hippocampus. Recordings were made using an Axopatch200B or HEKA patchclamp EPC800 amplifier, collected using PClamp9.2 or PClamp10.5 software (Molecular Devices; RRID:SCR_011323), sampled at a rate of 50 kHz and filtered at 10 kHz. The perfusion solution contained 144 mM NaCl, 2.5 mM KCl, 2.5 mM CaCl_2_, 10 mM HEPES, 1 mM NaH_2_PO_4_, and 10 mM glucose set to pH 7.4 and saturated with O_2_. Electrodes were prepared from glass capillaries with a resistance of 3–6 MΩ when filled with an internal solution containing 130 mM K‐gluconate, 4 mM NaCl, 0.5 mM CaCl_2_, 10 mM HEPES, 10 mM BAPTA, 4 mM MgATP, and 0.5 mM Na_2_GTP set to a pH of 7.2–7.4, and at an osmolarity of 290 ± 5 mOsm/kg. A correction for the resulting liquid junction potential was not applied (approximated as 15.6 mV; Clampex 11.1 software; Molecular devices).

Upon breakthrough, resting membrane potential (RMP), capacitance, membrane resistance, and the magnitude of the voltage‐gated inward (sodium) current, elicited by a voltage step from −60 mV to 20 mV, were recorded as previously described (Clarke et al., 2012). Cells with a voltage‐gated sodium channel current <60 pA were classified as newly differentiated oligodendrocytes (Clarke et al., 2012) and were consequently removed from analysis. Access resistance was measured before and after each recording and was between 12 and 25 MΩ (mean 19.2 ± 0.4 MΩ). Data were not included if the access resistance changed by ≥20% over the course of the recording or exceeded 25 MΩ. To determine the effect of bath‐applied 100 µM kainate (KA; Abcam), cells were voltage clamped at −60 mV and currents elicited by 200 ms voltage steps from −100 to 20 mV (20 mV increments). To measure the effect of bath‐applied 100 µM GABA (Sigma), cells were voltage clamped at 0 mV and currents elicited by 200 ms voltage steps from −80 to + 80 mV (20 mV increments). Recordings continued for a wash‐out period (2–10 min) after drug application to ensure that the health of the cell was not affected during the recordings of KA‐ or GABA‐evoked currents. The average steady state current magnitude, in the last 50 ms of the voltage step, was measured using clampfit 10.5 (molecular devices) and the evoked current (current in the presence of drug minus baseline current) reported. After recording the KA‐ or GABA‐evoked current, 6‐cyano‐7‐nitroquinoxaline‐2,3‐dione (CNQX; AMPA/KA receptor antagonist, 10 µM, Sigma) or picrotoxin (PTX, GABA_A_ receptor antagonist, 100 µM, Sigma) was bath applied for 2 min before reapplication of KA or GABA to the same cell.

#### 2.3.6 Immunohistochemistry and amyloid plaque detection

Tissue fixation and cryoprotection were performed as previously described (O'Rourke et al., 2016). Thirty micrometer coronal brain cryosections containing the entorhinal cortex, hippocampus, and fimbria (Bregma −1.34 to −2.70; Franklin & Paxinos, 2007) were collected and processed as floating sections. Cryosections were incubated for 1 hr at 21°C in blocking solution [10% fetal calf serum (FCS)/0.1% triton x‐100 in PBS] before being placed on an orbital shaker overnight at 4°C in blocking solution containing primary antibodies. The full list of primary antibodies used can be found in Table S1. In brief these include: goat anti‐platelet‐derived growth factor receptor α (PDGFRα, 1:100, R&D Systems; RRID:AB_2236897); rat anti‐GFP (1:2000, Nacalai Tesque; RRID:AB_10013361); rabbit anti‐OLIG2 (1:400; Merck Millipore; RRID:AB_570666); rabbit anti‐ASPA (1:200, Merck Millipore; RRID:AB_2827931); guinea pig anti‐Iba1 (1:250, Synaptic Systems; RRID:AB_2493179); mouse anti‐Caspr (1:200; Neuromab; RRID:AB_10806491); rabbit anti‐Nav1.6 (1:200; Alomone labs; RRID:AB_2040202); mouse anti‐6E10 (1:500, Covance; RRID:AB_662798); or rabbit anti‐MAP2 (1:1,000, Merck Millipore; RRID:AB_91939). Sections were washed thrice in PBS before being incubated overnight at 4°C on an orbital shaker in blocking solution containing secondary antibodies, conjugated to Alexa Fluors (Life Technologies Corporation): donkey anti‐rat 488 (1:500; RRID:AB_2535794); donkey anti‐rabbit 488 (1:1,000; RRID:AB_2535792); donkey anti‐rabbit 568 (1:1,000; RRID:AB_2534017); donkey anti‐rabbit 647 (1:1,000; RRID:AB_2536183); donkey anti‐goat 647 (1:1,000; RRID:AB_2535864); donkey anti‐mouse 647 (1:1,000; RRID:AB_162542); or goat anti‐guinea pig 488 (1:1,000; RRID:AB_2534117). Cell nuclei were visualized by the inclusion of Hoechst 33342 (1:10,000, Invitrogen). To detect amyloid plaques, tissue sections were transferred into 0.1% (w/v) Thioflavin S (Sigma)/60% (v/v) ethanol/40% (v/v) PBS, and agitated on a shaker for 3 min at 21°C. Sections were de‐stained by washing twice in 50% ethanol (v/v) in PBS and thrice in PBS. Floating sections were mounted onto glass slides and the fluorescence preserved by the application of fluorescent mounting medium (Dako Australia Pty. Ltd., Campbellfield, Australia).

#### 2.3.7 Confocal microscopy

For cell density quantification, confocal images were collected from the entorhinal cortex, hippocampus, and fimbria of *n* = 3 brain sections per mouse for each staining condition, using an Andor spinning disk confocal microscope with Nikon Software (Andor Technology Ltd., Belfast, Northern Ireland) or UltraView Nikon Ti spinning disk confocal microscope with Volocity software (Perkin Elmer, Waltham, USA). Images were collected using a 20x air objective (3 µm z‐spacing) with standard excitation and emission filters for DAPI, FITC (Alexa Fluor‐488), TRITC (Alexa Fluor 568) and CY5 (Alexa Fluor 647). Cell number and area measurements were performed manually using Fiji software (NIH, Washington DC, USA; RRID:SCR_003070) or Adobe Photoshop by an experimenter blind to the age and genotype of the mice imaged. High magnification (40× air objective) images were collected from any region of the hippocampus, fimbria, and entorhinal cortex and only serve to demonstrate the morphology of the labeled cells quantified.

To measure node and paranode lengths, Hoescht 33342 nuclear labeling was initially used to identify the CA1 region of the hippocampus or the fimbria, and to ensure the unbiased selection of non‐overlapping fields of view within each structure. Images were collected using an UltraView Nikon Ti spinning disk confocal microscope (100× oil objective; 0.5 µm z‐spacing). When nodes and their flanking paranodes were intact within a single *z*‐plane, they were measured manually using Fiji software (NIH, Washington DC, USA). For the CA1 region of the hippocampus, 30–90 nodes and paranodes were measured, sampled from four fields of view per mouse. For the fimbria, 36–94 nodes and paranodes were measured, sampled from two fields of view per mouse. All measurements were made by a researcher blind to genotype.

#### 2.3.8 Transmission electron microscopy

Mice were terminally anesthetized using sodium pentobarbital (i.p 100 mg/kg) and transcardially perfused with Karnovsky's fixative [0.8% (v/v) glutaraldehyde/2% (w/v) PFA/0.25 mM CaCl_2_/0.5 mM MgCl_2_ in 0.1 M sodium‐cacodylate buffer]. Brains were sliced into 2 mm thick coronal slices using a rodent brain matrix (Agar Scientific, Essex, UK) and immersion fixed at 21°C for 2 hr, before being stored in 0.1 M sodium‐cacodylate buffer overnight at 4°C. The stratum lacunosum moleculare of the Cornu Ammonis subfield 1 (CA1) of the hippocampus was dissected and immersed in 1% osmium tetroxide/1.5% potassium ferricyanide in 0.065 M sodium‐cacodylate buffer, in the dark, for 2 hr at 4°C. Tissue was washed five times in Milli‐Q water, before being dehydrated [70% ethanol (v/v) in Milli‐Q water overnight at 21°C; 80% ethanol (2 × 10 min); 85% ethanol (2 × 10 min); 90% ethanol (2 × 10 min); 95% ethanol (2 × 10 min); and 100% ethanol (4 × 10 min)]. Tissue was embedded by serial exposure to: 100% propylene oxide (2 × 5 min); 75% propylene oxide/25% epon (4 hr); 67% propylene oxide/33% epon (4 hr); 50% propylene oxide/50% epon (overnight); 33% propylene oxide/67% epon (4 hr); 25% propylene oxide/75% epon (4 hr), and 100% epon (overnight). Tissue was transferred to fresh 100% epon for 4 hr before being polymerized at 60°C for 72 hr.

Seventy nanometer ultramicrotome (Reichert Ultracut S, Leica) sections were collected using a diamond knife (Diatome) and were floated on Milli‐Q‐water. Floating sections were collected with a perfect loop (Diatome) and placed on a gold grid with formvar (ProSciTech) and stained with Reynolds' lead citrate stain [lead nitrate (Sigma) and trisodium citrate dihydrate (Merck)] and 4% uranyl acetate (Serva) in 50% ethanol to enhance the contrast. Electron micrographs were collected on a HT7700 (Hitachi) transmission electron microscope. Axons were identified based on their microtubule organization (reviewed by Stassart, Möbius, Nave, & Edgar, 2018) and individual myelin lamellae (wraps) identified by the presence of major dense lines (reviewed by Simons & Nave, 2016). The g‐ratio was measured for ≥95 myelinated axons per mouse, and the number of myelin wraps for ≥27 myelinated axons per mouse. Quantification was performed by an experimenter blind to genotype for *n* = 3 mice per group.

### 2.4 Statistical analyses

**Figure 1:**
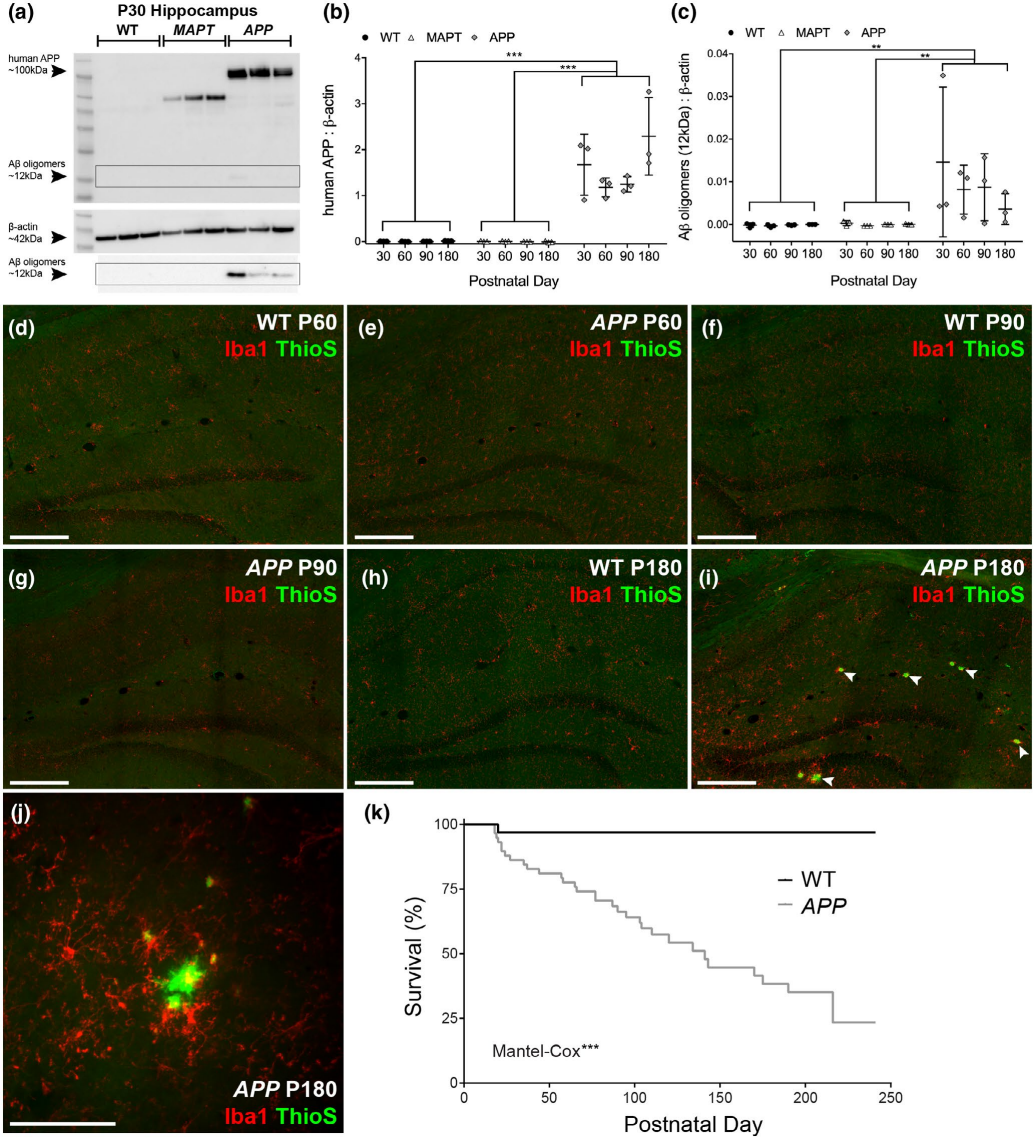
APP transgenic mice have impaired survival compared with their wildtype littermates. (a) A Western blot utilizing the anti‐human APP 6E10 antibody detects protein bands of ~100 kDa (human APP) and ~12 kDa (oligomerized amyloid β) in hippocampal protein lysates generated from P30 *APP* mice, that are absent from lysates generated from WT littermates and *Prnp‐MAPT^P301S^* (*MAPT*) transgenic mice. A protein band corresponding to β‐actin (~42 kDa) was detected in all hippocampal protein lysates. The boxed area shows the ~12 kDa oligomerized amyloid β after a longer imaging exposure. (b) Quantification of human APP expression, relative to β‐actin expression, in hippocampal lysates from P30, P60, P90, and P180 WT, *MAPT,* and *APP* transgenic mice, indicated that human APP expression was significantly elevated in *APP* mice relative to WT and *MAPT* mice at all timepoints [Two‐way ANOVA, genotype: *F* (2, 24) = 112.0, *p* < 0.001; age: *F* (3, 24) = 2.35, *p* = 0.097; interaction: *F* (6, 24) = 2.31, *p* = 0.066; *n* = 3 mice of each genotype analyzed at each age]. P180 *APP* transgenic mice expressed more human APP than P30, P60, or P90 mice of the same genotype. (c) Quantification of Aβ‐oligomer expression, relative to β‐actin expression, in hippocampal lysates from P30, P60, P90, and P180 WT, *MAPT,* and *APP* transgenic mice, indicated that Aβ‐oligomer expression was restricted to *APP* mice at all ages [Two‐way ANOVA, genotype: *F* (2, 24) = 9.06, *p* = 0.001; age: *F* (3, 24) = 0.60, *p* = 0.616; interaction: *F* (6, 24) = 0.74, *p* = 0.740, for each age group *n* = 3 WT, 3 *MAPT* and 3 *APP*]. (d–i) Coronal brain cryosections showing the hippocampus of P60, P90, and P180 WT and *APP* mice stained to detect the microglial marker Iba1 (red) and amyloid plaques (thioflavin S; green). White arrow heads indicate amyloid plaques. (j) A thioflavin S labeled amyloid plaque (green) surrounded by microglia (Iba1; red) in the hippocampus of a P180 *APP* transgenic mouse. (k) Quantification of the survival of WT and *APP* mice from birth until P241 [Log‐Rank (Mantel–Cox) test: Chi square (1) = 18.20, *n* = 32 WT and 58 *APP* mice]. *p < 0.05, **p < 0.01, ***p < 0.001 denote significant differences identified by Two‐way ANOVA, Bonferroni's post hoc analysis or Mantel–Cox test. Scale bar represents 200 µm (d–i) or 55 µm (j) [Color figure can be viewed at wileyonlinelibrary.com]

Statistical analyses were performed using GraphPad Prism 8.0 (La Jolla CA, USA; RRID:SCR_002798). Power analyses to determine sample size were not carried out a priori for the experiments included in this paper. Based on our previous experience with the techniques being applied, we aimed for sample sizes large enough to reliably detect a moderate effect size. For example, we aimed for a minimum of *n* = 12 mice per genotype per timepoint for behavioral analyses and *n* = 4 mice per genotype per timepoint for histological analyses. The large number of APP mice that died unexpectedly (Figure 1) limited the number of mice available for our experimental analyses and for some experiments or timepoints we were only able to obtain *n* = 3 mice in a group. These small sample sizes are a limitation to our study, as we do not have sufficient power to detect differences with an effect size smaller than 0.5 (Sullivan & Feinn, 2012). However, we consistently show large differences between WT and APP group means (effect sizes >1.0) and post hoc power analyses revealed that most comparisons had a statistical power >80% (G*Power 3.1; RRID:SCR_013726), with the exception being our *t*‐test comparison of average hippocampal node length per mouse (*n* = 3 mice per group), which only achieved a power of 58%.

The distribution of each data set was evaluated to determine whether the data were normally distributed using the d'Agostino and Pearson normality test or Shapiro–Wilk normality test where *n* ≥ 5. Data that were normally distributed were analyzed by a parametric test [one‐way analysis of variance (ANOVA) or two‐way ANOVA for group comparisons with a Bonferroni post hoc test], and data that were not normally distributed were analyzed using a Mann–Whitney *U* test or Kolmogorov–Smirnoff test. For data sets with *n* = 3 in any group, we were unable to test for normality, however, we applied parametric tests to analyze these data as the non‐parametric equivalents rely on ranking and are unreliable for small sample sizes (GraphPad Prism 8.0). A survival curve comparison was performed using a Log‐Rank (Mantel–Cox) test. Data are presented as mean ± standard deviation (*SD*). Statistical significance was established as *p* < 0.05. Statistical details are reported in each figure legend and individual data points are presented on each graph. Data supporting these findings will be made available by the corresponding author upon reasonable request.

## 3 RESULTS

### 3.1 *APP* mice develop histopathological features of Alzheimer's disease by P180

To confirm the expression of human APP in the brain of *APP* transgenic mice, we generated hippocampal protein lysates from WT, *Prnp‐MAPT^P301S^ (MAPT)* and *APP* mice at P30, P60, P90, and P180. By performing a series of Western blots, to detect immature and mature human APP and oligomerized amyloid β (6E10 antibody), we determined that human APP and oligomerized amyloid β were already expressed in the *APP* mouse hippocampus at P30, and that expression was relatively stable over time, but increased by P180 (Figure 1a–c). Human APP (~100 kDa) and amyloid β (~12 kDa) were not detected in hippocampal lysates from WT or *MAPT* transgenic mice (Figure 1b,c), which acted as an additional negative control for this experiment. A non‐specific protein band (~55 kDa) was detected in lysates from *MAPT* transgenic mice, however, this band does not correspond to human APP (Grant et al., 2019) but likely corresponds to human MAPT (Kalani et al., 2017; Pu et al., 2018), which shares a common epitope with amyloid β (Griner et al., 2019). To identify the cells that overexpress human APP within the hippocampus, coronal brain cryosections from P180 WT and *APP* mice were immunolabeled to detect human APP (6E10 antibody) and the neuronal marker microtubule associated protein 2 (MAP2), the mature oligodendrocyte marker aspartoacylase (ASPA), or the OPC marker, platelet‐derived growth factor α (PDGFRα) (Figure S1). Human APP was not expressed by WT mice, but was strongly expressed throughout the hippocampus and fimbria of *APP* mice (Figure S1). More specifically, human APP was strongly expressed by pyramidal, dentate granule, and mossy neurons in the hippocampus and by all ASPA^+^ mature oligodendrocytes, but not OPCs (PDGFRα^+^), in the hippocampus and fimbria of P180 *APP* mice (Figure S1).

To establish the time frame over which human APP expression caused the formation of amyloid plaques in the brains of *APP* transgenic mice, coronal brain cryosections from P60, P90, and P180 WT and *APP* mice were stained with thioflavin S (Figure 1d–j, green), which binds to β‐sheet structures and identifies amyloid β plaques (Bussière et al., 2004; Sun, Nguyen, & Bing, 2002). Cryosections were co‐labeled to detect the microglial marker Iba1 (Figure 1d–j, red). Plaques were absent from the hippocampus of WT and *APP* mice at P60 (Figure 1d,e) and P90 (Figure 1f,g), and while they were still absent from the hippocampus of P180 WT mice (Figure 1h), had formed in the hippocampus of P180 *APP* mice (Figure 1i,j). Furthermore, expression of the microglial marker Iba1 increased noticeably at P180 in the *APP* mice, indicative of reactive microgliosis, and microglia were observed to accumulate around the amyloid plaques (Figure 1j). While significant amyloid pathology was clearly observed in *APP* transgenic mice at P180, their survival was impaired from early adulthood. By quantifying the survival of WT and *APP* transgenic mice from birth until P180, we determined that ~60% of *APP* transgenic mice died prior to P180, compared with only ~3% of WT mice [Log‐rank (Mantel–Cox) test, *p* < 0.001, Figure 1k]. As we next aimed to characterize the behavioral consequences of *APP^Sw.Ind^* overexpression, it should be noted that the impaired survival of *APP* transgenic mice introduces an unavoidable bias into our analyses, skewing our characterization toward the less affected mice that survive to the older ages.

### 3.2 APP mice exhibit hyperactive behavior by P60 but do not develop spatial learning deficits by P180

**Figure 2:**
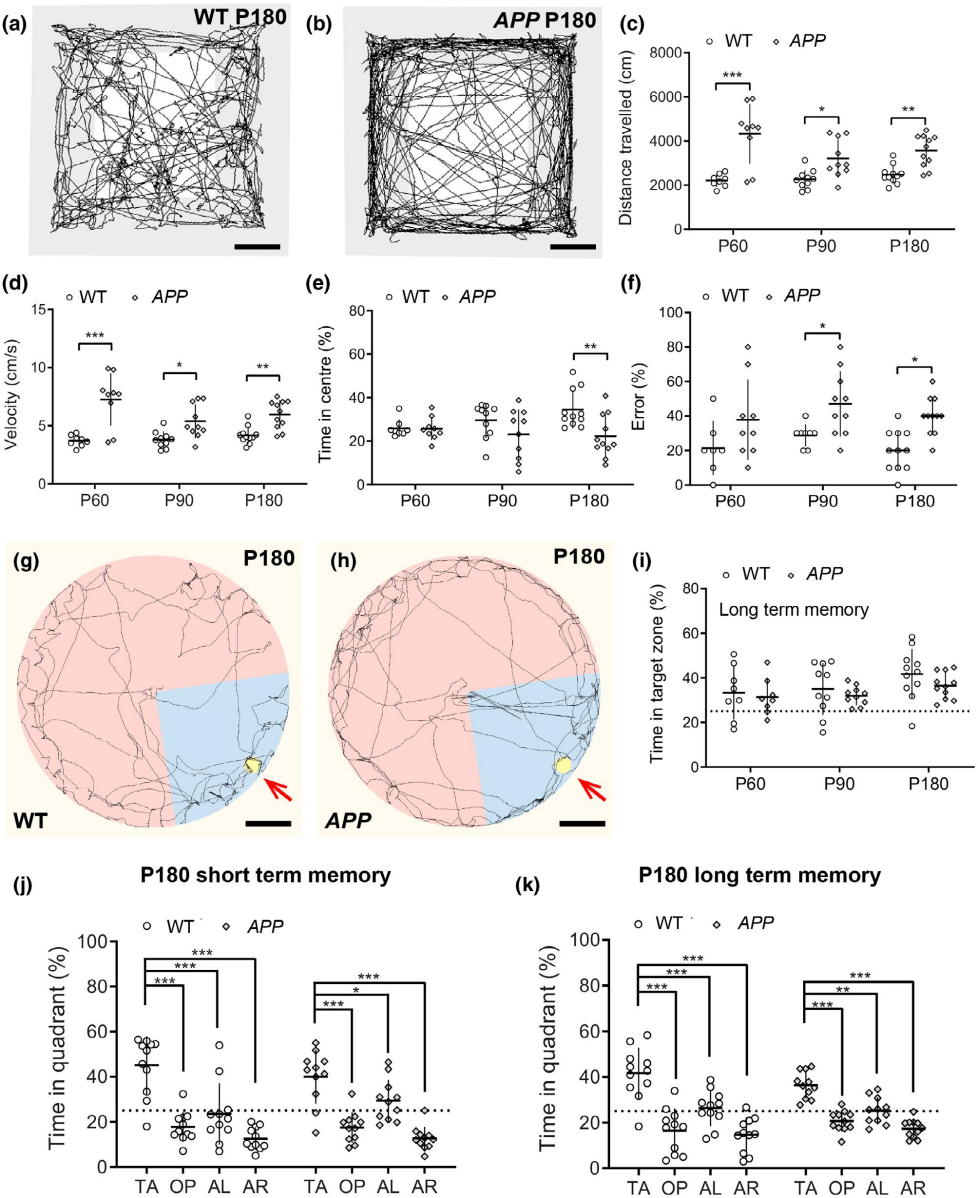
APP transgenic mice are hyperactive but show no overt learning and memory deficit by 6 months of age. (a–b) Representative track visualization images (EthoVision XT) showing movement (black lines) of P180 WT (a) and *APP* (b) mice during the open field task. (c) Quantification of the total distance traveled by WT and *APP* mice in the open field task at P60, P90, or P180 [Two‐way ANOVA, genotype: *F* (1, 53) = 48.59, *p* < 0.001; age: *F* (2, 53) = 2.30, *p* = 0.1; interaction: *F* (2, 53) = 3.18, *p* = 0.049]. (d) Quantification of the average movement velocity of WT and A*PP* mice during the open field task at P60, P90, or P180 [Two‐way ANOVA, genotype: *F* (1, 53) = 47.83, *p* < 0.001; age: *F* (2, 53) = 2.27, *p* = 0.1; interaction: *F* (2, 53) = 3.21, *p* = 0.047]. (e) Quantification of the proportion of time spent WT and A*PP* mice spend in the center of the open field during the open field task at P60, P90, or P180 [Two‐way ANOVA, genotype: *F* (1, 53) = 8.05, *p* = 0.01; age: *F* (2, 53) = 0.51, *p* = 0.6; interaction: *F* (2, 53) = 2.431, *p* = 0.1]. (f) Quantification of incorrect arm choices (% errors) made by P60, P90, or P180 WT and *APP* mice during the T‐maze alternation task [Two‐way ANOVA, genotype: *F* (1, 50) = 18.89, *p* < 0.001; age: *F* (2, 50) = 1.20, *p* = 0.2; interaction: *F* (2, 50) = 0.064, *p* = 0.9]. (g–h) Representative track visualization images (EthoVision XT) showing movement (black lines) of P180 WT (g) and *APP* (h) mice during the Barnes maze long‐term memory probe trial, carried out 2 weeks after mice learned the expected location of an escape box (red arrows). Blue shading indicates the quadrant of the maze defined as the target zone. (i) Quantification of the proportion of time P60, P90, or P180 WT or *APP* mice spent within the target zone during the long‐term memory probe trial [Two‐way ANOVA, genotype: *F* (1, 52) = 1.93, *p* = 0.170; age: *F* (2, 52) = 3.06, *p* = 0.055; interaction: *F* (2, 52) = 0.16, *p* = 0.844]. (j) Quantification of the time spent by P180 WT and *APP* mice in each quadrant of the Barnes maze during the short‐term memory probe phase [Two‐way ANOVA, genotype: *F* (1, 80) = 0.006, *p* = 0.9; maze quadrant: *F* (3, 80) = 41.96, *p* < 0.001; interaction: *F* (3, 80) = 1.212, *p* = 0.3]. (k) Quantification of the time P180 WT and *APP* mice spent in each quadrant of the Barnes maze during the long‐term memory probe phase [Two‐way ANOVA, genotype: *F* (1, 80) = 0.004, *p* = 0.9; maze quadrant: *F* (3, 80) = 42.55, *p* < 0.001; interaction: *F* (3, 80) = 1.77, *p* = 0.16]. Data are presented as mean ± *SD* for distinct WT and *APP* cohorts at each age (*n* = 8 P60 WT, 9 P60 *APP*, 10 P90 WT, 10 P90 *APP*, 11 P180 WT, and 11 P180 *APP* mice*)*. *p < 0.05, **p < 0.01, ***p < 0.001 denote significant differences identified by Bonferroni's post hoc analyses. TA: target quadrant; OP: opposite quadrant; AL: adjacent left quadrant; AR: adjacent right quadrant. Scale bars represent 5 cm (a–b) and 20 cm (g–h) [Color figure can be viewed at wileyonlinelibrary.com]

To compare the cognitive performance of WT and *APP* transgenic mice prior to and during plaque formation, WT and *APP* mice were subjected to a battery of behavioral tasks at P60, P90, or P180, with each age representing a separate cohort. WT (Figure 2a) and *APP* transgenic mice (Figure 2b) were first placed into an open field arena for 10 min, over which time the total distance each mouse traveled (Figure 2c), and the average velocity of their movement (Figure 1d) was recorded. At all ages tested, *APP* mice traveled further (Figure 2c) and faster (Figure 2d) than their WT littermates, suggesting that these mice are hyperactive. Additionally, WT and *APP* mice spent a similar proportion of time in the brightly lit center of the open field at P60 and P90, however, by P180 *APP* mice spent less of their time in the center region (Figure 2e), which is indicative of an increase in anxiety‐like behavior.

Working memory performance was subsequently evaluated by assessing spontaneous alternation in the T‐maze. We found that WT and *APP* mice performed similarly at P60, but that by P90 *APP* mice persistently made more repeated arm entries (errors) than their WT littermates (Figure 2f), suggesting that these mice have impaired working memory or attentional processing that is likely associated with their hyperactivity (Kim, Woo, Lee, & Yoon, 2017; Montarolo et al., 2019). When evaluating short‐ and long‐term memory retention by WT (Figure 2g) and *APP* transgenic mice (Figure 2h), using the Barnes maze spatial navigation task, we found that regardless of age, *APP* mice and their WT littermates performed equally well in the short‐term memory probe trial, 1 day after learning the location of the escape box [P60: WT 35.11 ± 6.9, *APP* 34.02 ± 9.2; P90: WT 40.35 ± 19.2, *APP* 44.90 ± 13.5; P180: WT 45.10 ± 12.8, *APP* 40.02 ± 11.8; mean ± *SD*, time in target zone (%)], and again 2 weeks later during the long‐term memory probe trial (Figure 2i). This is highlighted by data showing that even at P180, WT and *APP* mice spend significantly more time in the target quadrant, compared to all other quadrants during the short‐term (Figure 2j) and long‐term (Figure 2k) memory trials, indicating that mice of both genotypes learned and remembered the location of the escape box.

### 3.3 OPC density and membrane properties are unchanged but the response to GABA is increased at P100

**Figure 3:**
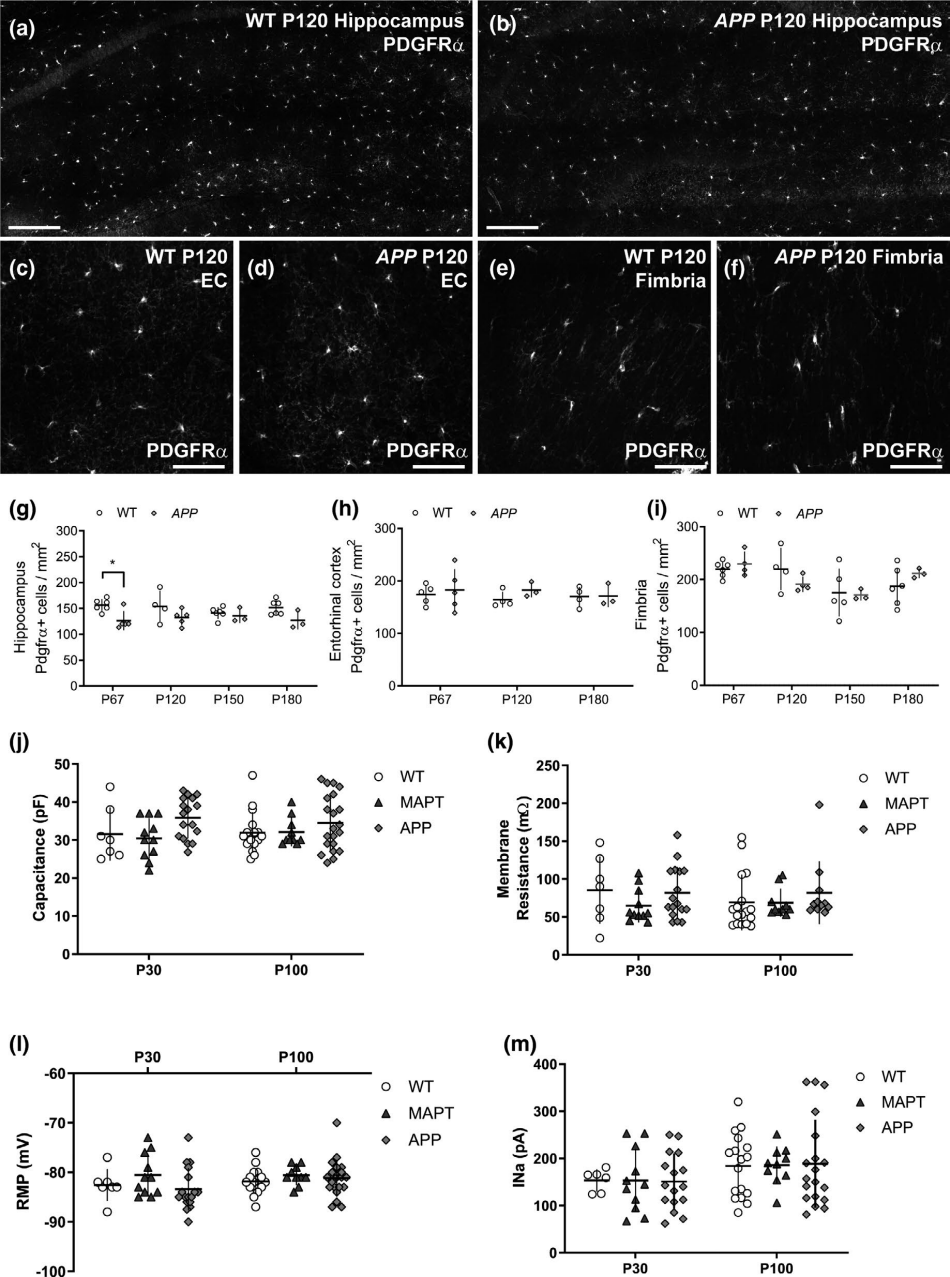
OPC density and membrane properties are normal in APP transgenic mice. (a–f) Coronal brain sections (30 µm) from P120 WT and *APP* mice were stained to detect PDGFRα^+^ OPCs in the hippocampus, entorhinal cortex, and fimbria. (g) Quantification of OPC density in the hippocampus of P60, P120, 150 and P180 WT and *APP* transgenic mice [Two‐way ANOVA, genotype: *F* (1, 30) = 13.66, *p* < 0.001; age: *F* (3, 30) = 0.14, *p* = 0.93; interaction: *F* (3, 30) = 0.91, *p* = 0.44; *n* = 6 P60 WT, 5 P60 *APP,* 4 P120 WT, 5 P120 *APP*, 6 P150 WT, 3 P150 *APP*, 6 P180 WT, and 3 P180 *APP* mice]. (h) Quantification of OPC density in the entorhinal cortex of P60, P120, and P180 WT and *APP* transgenic mice [Two‐way ANOVA, genotype: *F* (1, 18) = 0.85, *p* = 0.36; age: *F* (2, 18) = 0.22, *p* = 0.80; interaction: *F* (2, 18) = 0.20, *p* = 0.81; *n* = 5 P60 WT, 5 P60 *APP,* 4 P120 WT, 3 P120 *APP*, 4 P180 WT and 3 P180 *APP* mice]. (i) Quantification of OPC density in the fimbria of P60, P120, 150 and P180 WT and *APP* transgenic mice [Two‐way ANOVA, genotype: *F* (1, 27) = 0.0009, *p* = 0.97; age: *F* (3, 27) = 4.49, *p* = 0.01; interaction: *F* (3, 27) = 1.23, *p* = 0.31; *n* = 6 P60 WT, 4 P60 *APP,* 4 P120 WT, 4 P120 *APP*, 5 P150 WT, 3 P150 *APP*, 6 P180 WT, and 3 P180 *APP* mice]. (j) The membrane capacitance of OPCs in the hippocampus of P30 and P100 WT, *MAPT* and *APP* mice [Two‐way ANOVA, genotype: *F* (2, 77) = 3.748, *p* = 0.03; age: *F* (1, 77) = 0.03 *p* = 0.87; interaction: *F* (2, 77) = 0.45, *p* = 0.64; P30 *n* = 7 WT, 11 *MAPT* and 17 *APP*; P100 *n* = 17 WT, 10 *MAPT* and 21 *APP*]. (k) The membrane resistance of OPCs in the hippocampus of P30 and P100 WT, *MAPT,* and *APP* mice [Two‐way ANOVA, genotype: *F* (2, 67) = 1.173, *p* = 0.31; age: *F* (1, 67) = 0.24, *p* = 0.63; interaction: *F* (2, 67) = 0.49, *p* = 0.61; P30 *n* = 7 WT, 11 *MAPT* and 17 *APP*; P100 *n* = 17 WT, 10 *MAPT* and 11 *APP*]. (l) The resting membrane potential of OPCs in the hippocampus of P30 and P100 WT, *MAPT,* and *APP* mice [Two‐way ANOVA, genotype: *F* (2, 77) = 1.7166, *p* = 0.18; age: *F* (1, 77) = 1.35, *p* = 0.25; interaction: *F* (2, 77) = 0.75, *p* = 0.48; P30 *n* = 7 WT, 11 *MAPT* and 17 *APP*; P100 *n* = 17 WT, 10 *MAPT* and 21 *APP*]. (m) The voltage‐gated sodium channel current (I_Na_) recorded from OPCs in the hippocampus of P30 and P100 WT, *MAPT,* and *APP* mice [Two‐way ANOVA, genotype: *F* (2, 73) = 0.002, *p* = 0.998; age: *F* (1, 73) = 4.1, *p* = 0.047; interaction: *F* (2, 73) = 0.019, *p* = 0.98; P30 *n* = 6 WT, 11 *MAPT* and 16 *APP*; P100 *n* = 17 WT, 10 *MAPT* and 19 *APP*]. Data are presented as mean ± *SD*. *p < 0.05 denote significant differences identified by Bonferroni's post hoc analyses. Scale bars represent 185 µm (a, b) and 70 µm (c–f)

To determine how OPC behavior might be affected by amyloid pathology, we first quantified the density of PDGFRα^+^ OPCs in the hippocampus, entorhinal cortex, and fimbria of WT and *APP* mice (Figure 3a–i). We found that OPC density was slightly reduced in the hippocampus of *APP* mice compared with control mice at P67, however, this difference was not maintained at later ages (Figure 3g). In the entorhinal cortex (Figure 3h) and fimbria (Figure 3i), OPC density was not affected by genotype and remained stable over time. To determine whether amyloid pathology affected the membrane properties of OPCs, we performed whole‐cell patch‐clamp analysis of GFP^+^ OPCs in the hippocampus of brain slices collected from WT, *MAPT,* or *APP* transgenic mice carrying the *Pdgfrα–H2BGFP* transgene. We report that neither the expression of *MAPT^P301S^* nor *APP^Sw.Ind^* altered the OPC membrane capacitance (an approximate measure of cell size; Figure 3j), membrane resistance (Figure 3k), or resting membrane potential (Figure 3l), which were equivalent for WT, *MAPT,* and *APP* transgenic mice at P30 and P100. Furthermore, the magnitude of the inwards voltage‐gated sodium channel current recorded from P30 and P100 OPCs was equivalent for WT, *MAPT,* and *APP* transgenic mice (Figure 3m).

**Figure 4:**
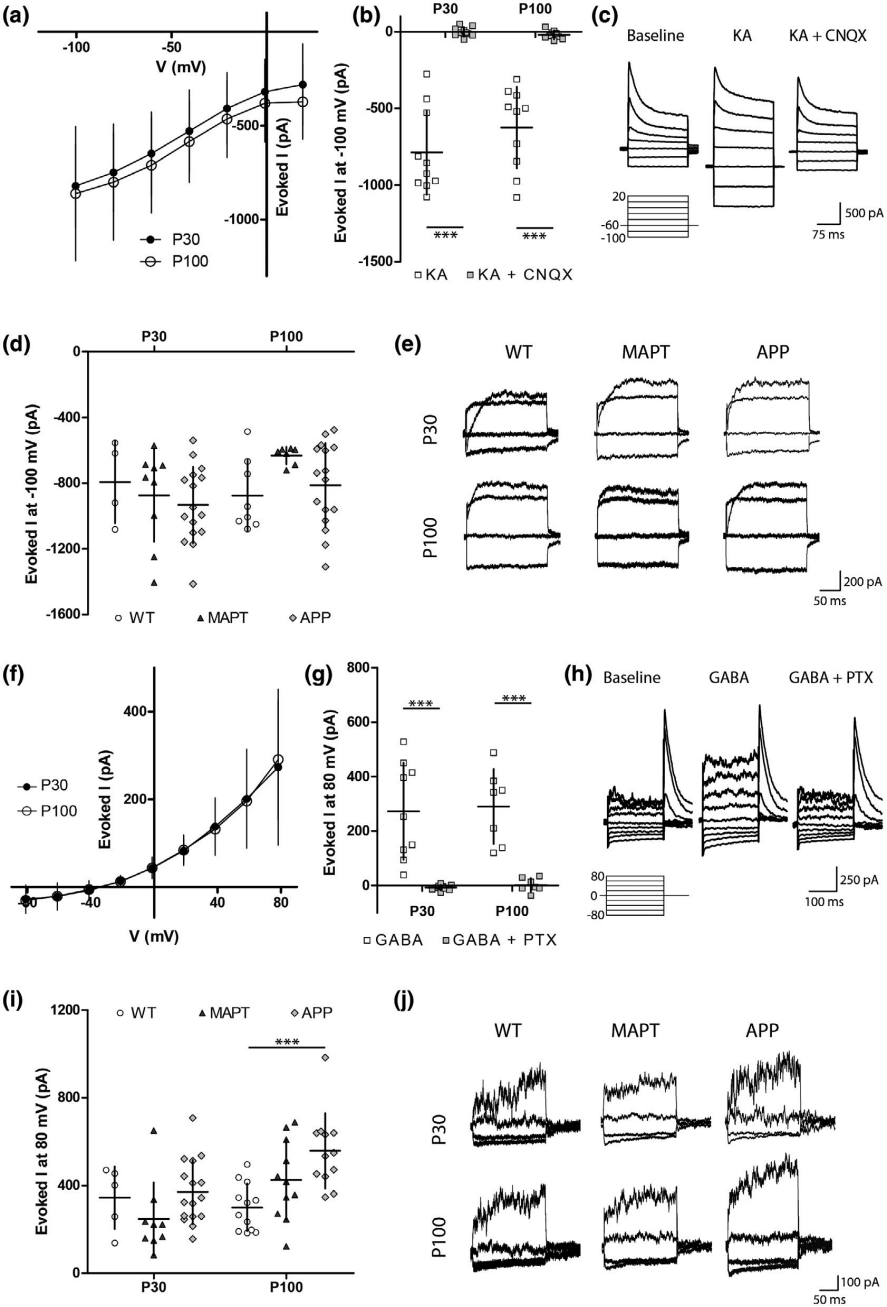
OPCs from APP transgenic mice have a heightened response to GABA. (a) I–V relationship for the current evoked in hippocampal OPCs by the bath application of 100 µM KA (mean steady state baseline current was subtracted from the mean steady state current in the presence of KA) in P30 or P100 WT mice. Each cell was submitted to multiple voltage steps [Repeated measures two‐way ANOVA, age: *F* (1, 8) = 0.2372, *p* = 0.6; voltage: *F* (1.135, 9.079) = 10.62, *p* = 0.008; interaction: *F* (6, 48) = 0.01539, *p* = 1; *n* = 3 P30 and 7 P100]. (b) Quantification of kainate (100 µM) evoked currents when hippocampal OPC are hyperpolarized to −100 mV in P30 or P100 WT mice in the presence and absence of CNQX (10 µM) [Repeated measures two‐way ANOVA, drug effect: *F* (1, 35) = 124.3, *p* < 0.001; age: *F* (1, 35) = 1.302, *p* = 0.3; interaction: *F* (1, 35) = 2.116, *p* = 0.2; *n* = 9 P30 and 10 P100]. (c) Example traces show the baseline currents, currents in the presence of kainate, or currents in the presence of kainate + CNQX from OPCs of WT mice after a family of voltage steps from −100 to 20 mV (20 mV increments). (d) Quantification of kainate‐evoked currents measured after a hyperpolarizing pulse (to −100 mV) for OPCs in the hippocampus of P30 or P100 WT, *MAPT,* or APP transgenic mice [Two‐way ANOVA, genotype: *F* (2, 54) = 0.1375 *p* = 0.3; age: *F* (1, 54) = 1.893, *p* = 0.2; interaction: *F* (2, 54) = 0.2252, *p* = 0.2; P30 *n* = 4 WT, 11 *MAPT* and 14 *APP*; P100 *n* = 8 WT, 9 *MAPT* and 14 *APP*]. (e) Example traces show KA‐evoked currents (baseline current was subtracted from currents recorded in the presence of KA) after voltage steps form −100 mV to 20 mV (40 mV increments) in P30 or P100 WT, *MAPT* or *APP* mice. (f) I–V relationship for the current evoked in hippocampal OPCs by the bath application of 100 µM GABA (mean steady state baseline current subtracted from the mean steady state current in the presence of GABA) in P30 or P100 WT mice. Each cell was submitted to multiple voltage steps [Repeated measures two‐way ANOVA, age: *F* (1, 14) = 0.002744, *p* = 1; voltage: *F* (1.123, 15.73) = 45.16, *p* < 0.001; interaction: *F* (8, 112) = 0.04518, *p* = 1; *n* = 9 P30 and 7 P100]. (g) Quantification of the GABA‐evoked current measured after hyperpolarizing hippocampal OPCs to −80 mV in the absence and presence of picrotoxin (50 µM) [Repeated measures two‐way ANOVA, drug effect: *F* (1, 28) = 47.98 *p* < 0.001; age: *F* (1, 28) = 0.1120, *p* = 0.7; interaction: *F* (1, 28) = 0.0087, *p* = 0.9; *n* = 8 P30 and 7 P100]. (h) Example traces showing the baseline currents, currents in the presence of GABA, or currents in the presence of GABA + picrotoxin from OPCs in hippocampal slices from WT mice after a family of voltage steps from −80 to 80 mV (20 mV increments). (i) Quantification of the GABA‐evoked current measured after a hyperpolarizing pulse to −80 mV in OPCs from P30 or P100 WT, *MAPT,* or *APP* mice [Two‐way ANOVA, genotype: *F* (2, 59) = 5.738, *p* = 0.005; age: *F* (1, 59) = 6.882, *p* = 0.01; interaction: *F* (2, 59) = 2.964, *p* = 0.06; P30 *n* = 5 WT, 9 *MAPT* and 16 *APP;* P100 *n* = 12 WT, 11 *MAPT* and 12 *APP*]. (j) Example traces show GABA‐evoked currents (baseline current was subtracted from currents recorded in the presence of GABA) after voltage steps from −80 mV to 80 mV (40 mV increments) for hippocampal OPCs from P30 or P100 WT, *MAPT,* or *APP* mice. Values represent mean ± *SD*. ***p < 0.001 denote significant differences identified by Bonferroni's post hoc analyses

*APP* mice are hyperactive by P60—a phenotype that may reflect altered neurotransmitter signaling or an inhibitory–excitatory imbalance in the brain (Palop et al., 2007; Sanchez et al., 2012; Snowden et al., 2019; Verret et al., 2012), therefore, we next used whole‐cell patch‐clamp electrophysiology to examine the ability of OPCs in the hippocampus of WT, *MAPT,* and *APP* mice to respond to excitatory and inhibitory neurotransmitters. GFP^+^ OPCs from WT mice were first held at −60 mV and KA (100 μM) was bath applied to activate the ionotropic AMPA/KA subtype of glutamate receptors. KA application evoked an inward current that was sensitive to the AMPA/KA receptor antagonist CNQX (Figure 4a–c). The KA‐evoked current in OPCs at P30 and P100 had a non‐ohmic voltage dependence and did not reverse (Figure 4a), which is consistent with previous reports showing that in OPCs sodium entry through AMPA/KA receptors inhibits the delayed rectifier voltage‐gated potassium channel current at depolarized potentials (Borges & Kettenmann, 1995). We found that the amplitude of the KA‐evoked current did not differ between P30 and P100, and was equivalent in WT, *MAPT,* and *APP* transgenic mice (Figure 4d,e).

To assess the response of OPCs to the inhibitory neurotransmitter GABA (100 μM), OPCs were held at 0 mV while GABA was bath applied. At P30 and P100, GABA evoked an outwardly rectifying current that was completely abolished in the presence of PTX (100 μM; Figure 4f–h), indicating that the evoked currents resulted from the activation of ionotropic GABA_A_ receptors. The GABA‐evoked current reversed at a more positive potential (−34 mV − 15.6 mV liquid junction potential approximation = ~−49.6 mV) than the expected reversal potential for chloride as calculated by the Nernst equation (~−85 mV), suggesting that the evoked current was not a pure chloride current and may, for example, also include a component that is the result of GABA_A_ mediated changes in leak potassium currents (such as the GABA_A_ mediated inhibition of two‐pore domain potassium channels demonstrated in hippocampal astrocytes; Ma, Xie, & Zhou, 2012). The amplitude of the current evoked by GABA in OPCs from P30 WT, *MAPT,* and *APP* transgenic mice did not differ (Figure 4i,j). By contrast, OPCs in hippocampal slices generated from P100 *APP* transgenic mice responded more robustly to GABA at 80 mV than OPCs from P100 WT or *MAPT* mice (Figure 4i,j). As membrane resistance was not changed in *APP* mice (Figure 3k), even if there was a potassium component to the GABA_A_ evoked current, the increased response to GABA is unlikely to be due to changes in leak potassium signaling. These data suggest that the overexpression of human APP in neurons or oligodendrocytes, or early amyloid pathology, is associated with a change in the subunit composition of GABA_A_ receptors expressed by OPCs, a change in the number of GABA_A_ receptors expressed on the cell surface, or a change in downstream signaling as a result of GABA_A_ receptor activation.

### 3.4 Node of Ranvier length is decreased and paranode length increased in the hippocampus of P100 *APP* mice

**Figure 5:**
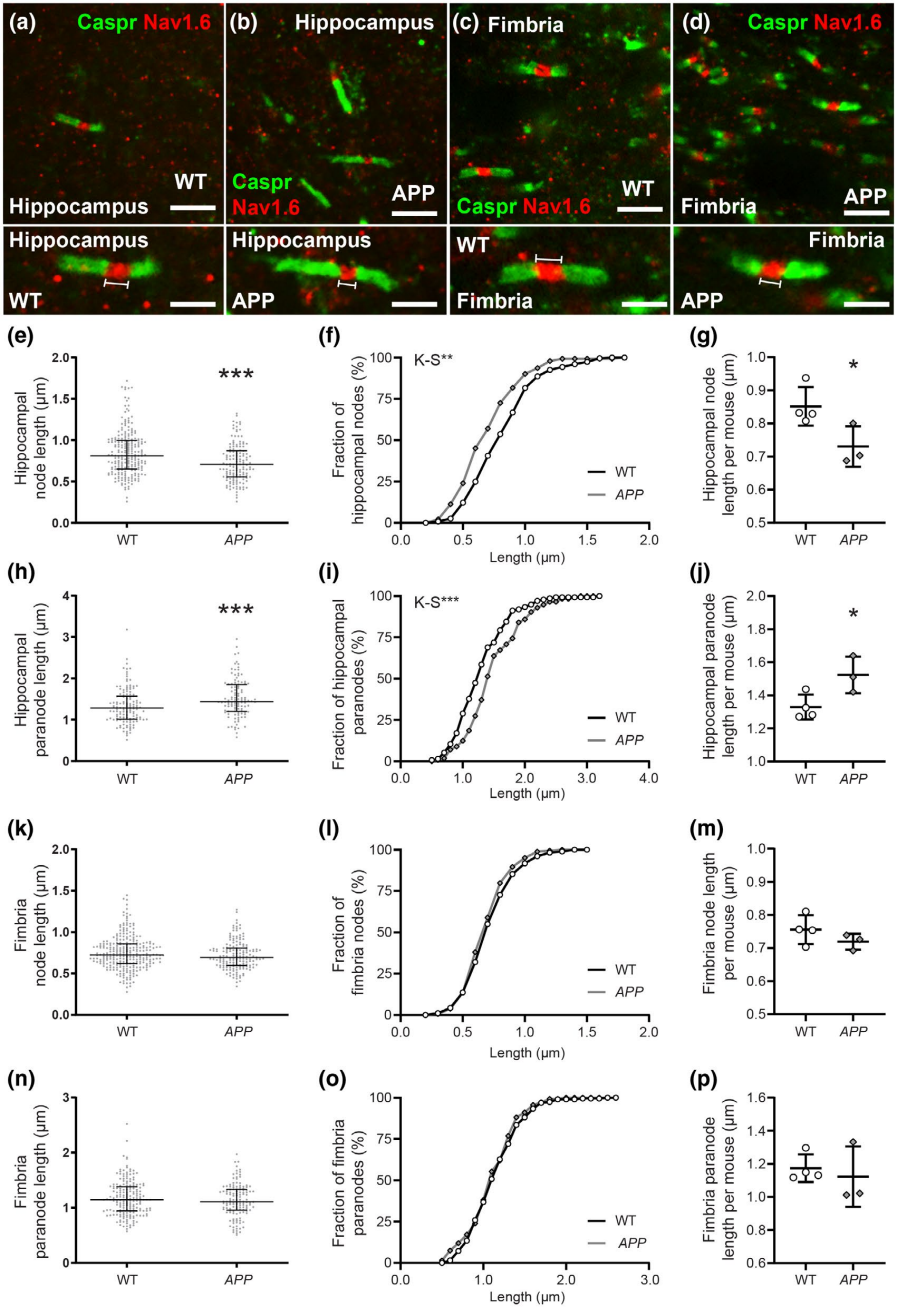
Nodes of Ranvier are shorter and paranodes longer in the hippocampus of APP transgenic mice. (a–d) 100× magnification confocal images of coronal brain sections (30 µm) from P107 WT and *APP* mice that were stained to detect Caspr (green; paranodes) and Nav1.6 (red; nodes of Ranvier) in the hippocampus and fimbria. Magnified panels depict example nodes of Ranvier that lay within a single *z*‐plane and were used for length measurements. (e) Quantification of node length in the hippocampus of WT (*n* = 229 nodes) and *APP* (*n* = 142 nodes) mice [Mann–Whitney test, *U* = 11990]. (f) Cumulative distribution plot of node length in the hippocampus of WT (open circles) and *APP* mice (gray diamonds) [Kolmogorov–Smirnov test *D* = 0.2033]. (g) Quantification of mean hippocampal node length for each WT (*n* = 4) or *APP* (*n* = 3) mouse [Two‐tailed, unpaired *t* test *t* (5) = 2.664]. (h) Quantification of paranode length in the hippocampus of WT (*n* = 135 paranodes) and *APP* (*n* = 113 paranodes) mice [Mann–Whitney test, *U* = 5568]. (i) Cumulative distribution plot of paranode length in the hippocampus of WT and *APP* mice [Kolmogorov–Smirnov test *D* = 0.2507]. (j) Quantification of mean hippocampal paranode length for each WT (*n* = 4) or *APP* (*n* = 3) mouse [Two‐tailed, unpaired *t* test, *t* (5) = 2.799]. (k) Quantification of node length in the fimbria of WT (*n* = 278 nodes) and *APP* (*n* = 163 nodes) mice [Mann–Whitney test, *U* = 20500]. (l) Cumulative distribution plot of node length in the fimbria of WT and *APP* mice [Kolmogorov–Smirnov test *D* = 0.119]. (m) Quantification of mean hippocampal node length for each WT (*n* = 4) or *APP* (*n* = 3) mouse [Two‐tailed, unpaired *t* test, *t* (5) = 1.293]. (n) Quantification of paranode length in the hippocampus of WT (*n* = 193 paranodes) and *APP* (*n* = 134 paranodes) mice [Mann–Whitney test, *U* = 12280]. (o) Cumulative distribution plot of paranode length in the hippocampus of WT and *APP* mice [Kolmogorov–Smirnov test, *D* = 0.08391]. (p) Quantification of mean hippocampal paranode length for each WT (*n* = 4) or *APP* (*n* = 3) mouse [Two‐tailed, unpaired *t* test, *t* (5) = 0.5096]. Results are presented as mean ± *SD*. *p < 0.05, **p < 0.01 and ***p < 0.001 denote significant differences identified by Mann–Whitney test, Kolmogorov–Smirnov test, or unpaired *t* test. Scale bars represent 2.8 µm (a–d) or 1.4 µm (magnified nodes below a–d) [Color figure can be viewed at wileyonlinelibrary.com]

To determine whether myelin integrity was affected in young adult *APP* transgenic mice, we first examined the morphology of the nodes of Ranvier and their associated paranodes in P100 WT and *APP* transgenic mice (Figure 5). Coronal brain cryosections containing the hippocampus (Figure 5a,b) and fimbria (Figure 5c,d) were immunolabeled to detect the nodal protein NaV1.6 (red) and the paranodal protein Caspr (green). We measured the length of each of these structures from confocal micrographs, and found that node of Ranvier length was shorter in the hippocampus of *APP* transgenic compared to WT mice (Figure 5e), with node length distribution being significantly shifted toward the formation of shorter nodes (Figure 5f). Furthermore, average node length per mouse was also reduced with *APP^Sw.Ind^* expression (Figure 5g). The observed change in node length was accompanied by a lengthening of the paranodes in the hippocampus of *APP* transgenic mice (Figure 5h), as paranode length distribution was shifted toward the generation of longer paranodes (Figure 5i). Within the hippocampus of *APP* transgenic mice, average paranode length per mouse was also increased (Figure 5j). By contrast, when node of Ranvier (Figure 5k–m) and paranode lengths (Figure 5n–p) were quantified in the fimbria, each was found to be equivalent in WT and *APP* transgenic mice, suggesting that this phenotype is region specific.

### 3.5 Myelin thickness is increased in the hippocampus of P100 *APP* transgenic mice

**Figure 6:**
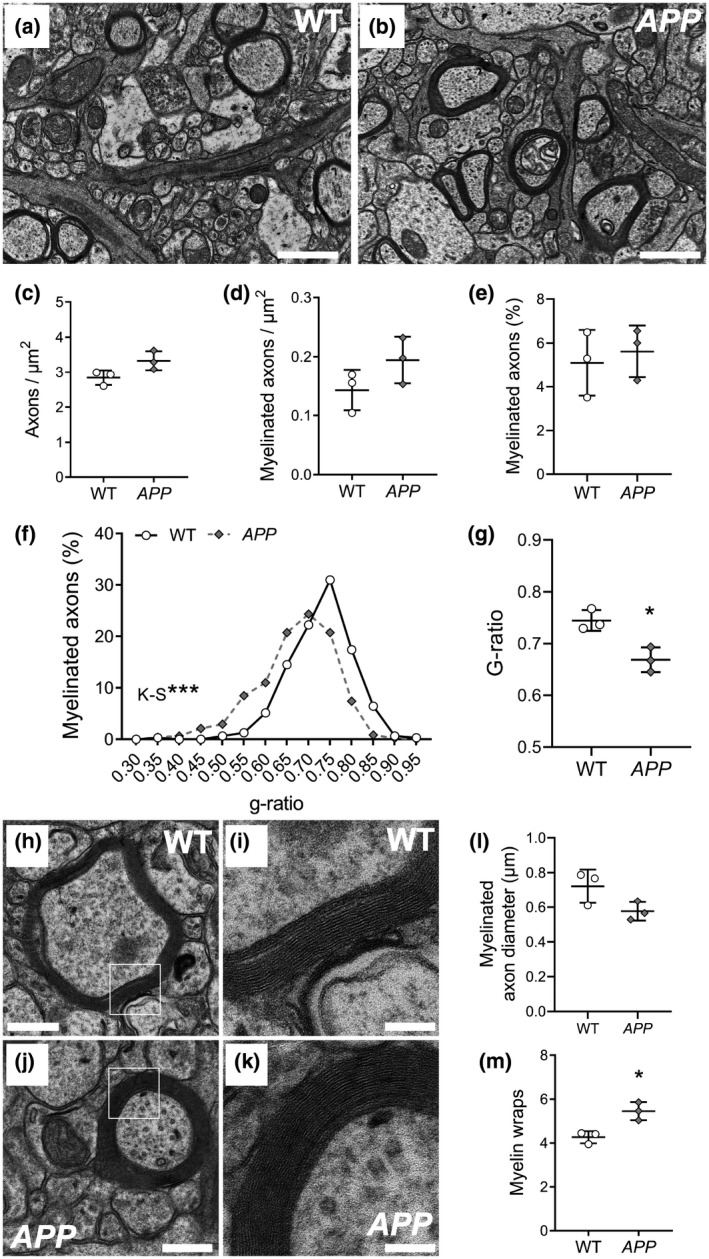
Myelin thickness is increased in APP transgenic mice. (a–b) Representative electron micrographs from the CA1 region of the hippocampus of WT (a) and *APP* (b) mice at P90. (c) Quantification of total axon density (axons/µm^2^) in WT (open circles) and *APP* (gray diamonds) mice [Two‐tailed, unpaired *t* test, *t* (4) = 2.46]. (d) Quantification of myelinated axon density (axons/µm^2^) in WT and *APP* mice [Two‐tailed, unpaired *t* test, *t* (4) = 1.69]. (e) Quantification of the proportion of myelinated axons in the CA1 of WT and *APP* mice at P90 [Two‐tailed, unpaired *t* test, *t* (4) = 0.46]. (f) Relative frequency distribution plot showing g‐ratio measurements from myelinated axons in the CA1 of P90 WT and *APP* mice [Kolmogorov–Smirnoff test, K‐S *D* = 0.2926; *n* = 310 WT and 472 *APP* axons analyzed from *n* = 3 mice per group]. (g) Quantification of average g‐ratio for WT and *APP* mice [Two‐tailed, unpaired *t* test: *t* (4) = 4.18]. (h–k) Representative high magnification electron micrographs of transected myelinated axons within CA1 of WT (h–i) and *APP* mice (j–k) at P90. (l) Average myelinated axon diameter within CA1 of WT and *APP* mice [Two‐tailed, unpaired *t* test, *t* (4) = 2.27]. (m) Quantification of average number of myelin wraps for axons within CA1 of WT and *APP* mice at P90 [Two‐tailed, unpaired *t* test, *t* (4) = 4.12]. Results are presented as mean ± *SD*, *n* = 3 WT mice and 3 *APP* mice. *p < 0.05 and ****p < 0.001 denote significant differences identified by Kolmogorov–Smirnov or unpaired *t* tests. Scale bars represent: 1 µm (a–b), 300 nm (h, j) or 100 nm (i, k)

As paranode lengthening can result from myelin decompaction (Howell et al., 2006; Stojic, Bojcevski, Williams, Diem, & Fairless, 2018) or an increased number of myelin wraps (Jeffries et al., 2016; Snaidero et al., 2014), we next examined the ultrastructure of hippocampal myelin in P100 WT (Figure 6a) and *APP* mice (Figure 6b) by transmission electron microscopy. We found that axon density (Figure 6c), myelinated axon density (Figure 6d), and the proportion of axons that are myelinated (Figure 6e) was equivalent between WT and *APP* mice, suggesting that axon number and the proportion of axons that get myelinated during development is not affected by genotype. However, the g‐ratio [axon diameter/(axon + myelin diameter)] of myelinated axons in the hippocampus of *APP* mice was reduced relative to WT mice (Figure 6f–g), suggesting that *APP* mice have thicker hippocampal myelin. This was confirmed when we measured the diameter of the myelinated axons (Figure 6h–l) and found that this was equivalent in WT and *APP* mice, confirming that the physical size of the axons was not driving the change in myelin thickness. Furthermore, we found that the number of myelin lamellae (wraps) surrounding each axon was increased in *APP* mice (Figure 6m). These data indicate that overexpressing human APP in neurons and oligodendrocytes, or early amyloid pathology influences the myelinating behavior of oligodendrocytes in the hippocampus.

### 3.6 New oligodendrocyte number is elevated in the hippocampus, entorhinal cortex, and fimbria of adult *APP* transgenic mice

**Figure 7:**
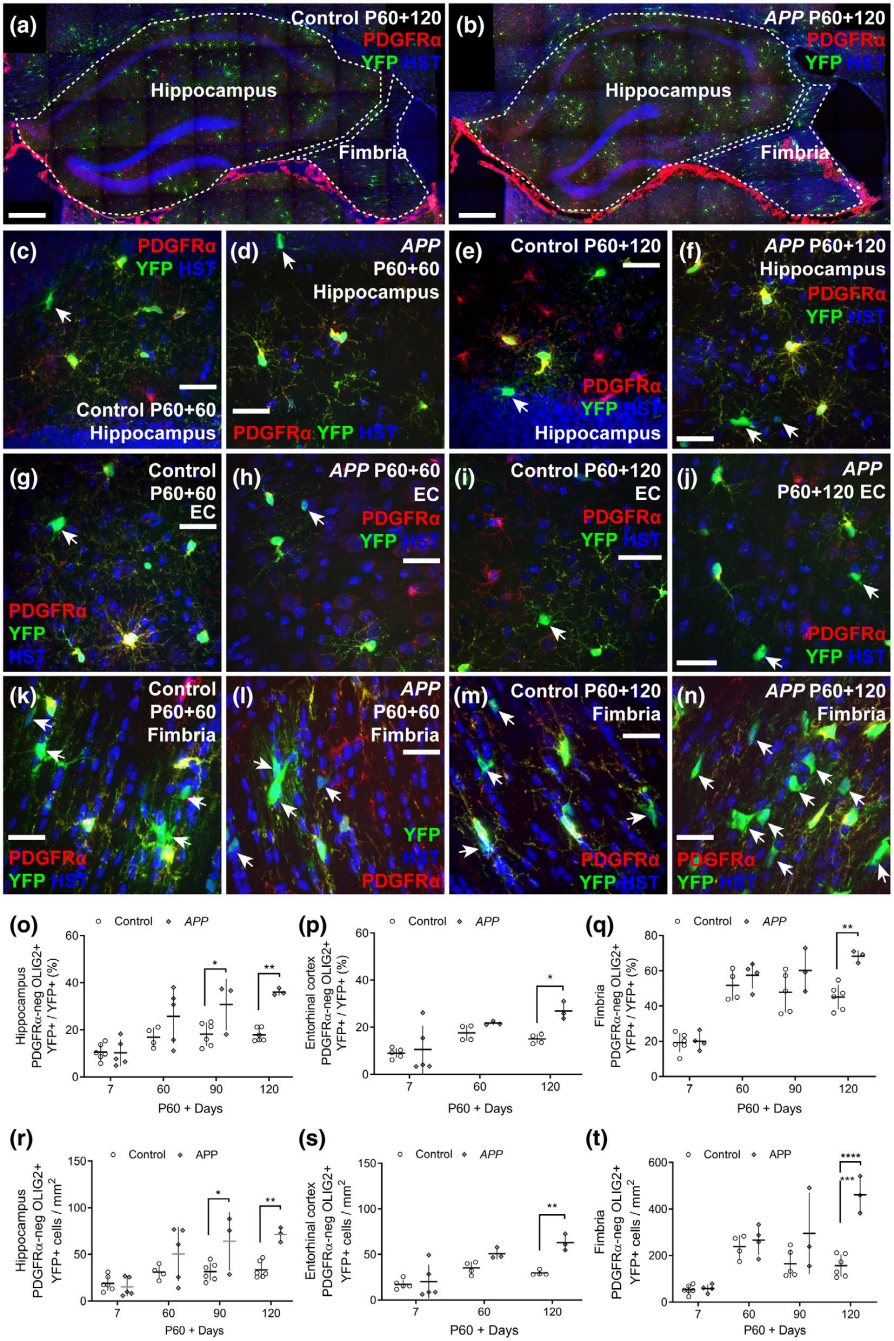
Adult oligodendrogenesis is elevated in the *APP* transgenic mouse brain. P60 control and *APP* mice received tamoxifen at P60, inducing recombination of the Cre‐sensitive YFP reporter, effectively labeling OPCs and their progeny. Immunohistochemistry was performed on brain cryosections from mice at P60 + 7 (P67), P60 + 60 (P120), P60 + 90 (P150), and P60 + 120 (P180). (a–b) Low magnification confocal images indicating the hippocampus and fimbria in P60 + 120 control and *APP* mice [YFP (green), PDGFRα (red) and Hoechst 33342 (blue)]. (c–f) Confocal images from the hippocampus of P60 + 60 and P60 + 120 control and *APP* transgenic mice showing cells labeled with YFP (green), PDGFRα (red), and Hoechst 33342 (blue). (g–j) Confocal images from the entorhinal cortex of P60 + 60 and P60 + 120 control and *APP* transgenic mice showing cells labeled with YFP (green), PDGFRα (red), and Hoechst 33342 (blue). (k–n) Confocal images from the fimbria of P60 + 60 and P60 + 120 control and *APP* transgenic mice showing cells labeled with YFP (green), PDGFRα (red), and Hoechst 33342 (blue). (o) Quantification of the proportion of YFP^+^ cells in the hippocampus of control and *APP* mice that have differentiated into PDGFRα‐negative OLIG2^+^ oligodendrocytes [Two‐way ANOVA, genotype: *F* (1, 30) = 21.35, *p* < 0.001; age: *F* (3, 30) = 13.12, *p* < 0.001; interaction: *F* (3, 30) = 3.62, *p* = 0.024]. (p) Quantification of the proportion of YFP^+^ cells in the entorhinal cortex of control and *APP* mice that have differentiated into PDGFRα‐negative OLIG2^+^ oligodendrocytes [Two‐way ANOVA, genotype: *F* (1, 18) = 7.01, *p* = 0.016; age: *F* (2, 18) = 11.61, *p* < 0.001; interaction: *F* (2, 18) = 2.03, *p* = 0.16]. (q) Quantification of the proportion of YFP^+^ cells in the fimbria that have differentiated of control and *APP* mice that have differentiated into PDGFRα‐negative OLIG2^+^ oligodendrocytes [Two‐way ANOVA, genotype: *F* (1, 27) = 14.29, *p* < 0.001; age: *F* (3, 27) = 45.15, *p* < 0.001; interaction: *F* (3, 27) = 3.11, *p* = 0.042]. (r) Quantification of the density of YFP^+^ OLIG2^+^ PDGFRα‐negative newborn oligodendrocytes in the hippocampus of control and *APP* transgenic mice (cells per mm^2^ as adjusted for *x*–*y* area only) [Two‐way ANOVA, genotype: *F* (1, 30) = 16.92, *p* < 0.001; age: *F* (3, 30) = 9.97, *p* < 0.001; interaction: *F* (3, 30) = 3.41, *p* = 0.029]. (s) Quantification of the density of YFP^+^ OLIG2^+^ PDGFRα‐negative newborn oligodendrocytes in the entorhinal cortex of control and *APP* transgenic mice (oligodendrocytes per mm^2^ as adjusted for *x*–*y* area only) [Two‐way ANOVA, genotype: *F* (1, 18) = 16.16, *p* < 0.001; age: *F* (2, 18) = 18.50, *p* < 0.001; interaction: *F* (2, 18) = 4.41, *p* = 0.027]. (t) Quantification of the density of YFP^+^ OLIG2^+^ PDGFRα‐negative newborn oligodendrocytes in the fimbria of control and *APP* transgenic mice (oligodendrocytes per mm^2^ as adjusted for *x*–*y* area only) [Two‐way ANOVA, genotype: *F* (1, 27) = 24.73, *p* < 0.001; age: *F* (3, 27) = 23.68, *p* < 0.001; interaction: *F* (3, 27) = 8.55, *p* < 0.001]. Data are presented as mean ± *SD*. Control hippocampus *n* = 6 P60 + 7, 4 P60 + 60, 6 P60 + 90, and 6 P60 + 120; *APP* hippocampus *n* = 5 P60 + 7, 5 P60 + 60, 3 P60 + 90, and 3 P60 + 120; control entorhinal cortex *n* = 5 P60 + 7, 4 P60 + 60, and 4 P60 + 120; *APP* entorhinal cortex *n* = 5 P60 + 7, 3 P60 + 60 and 3 P60 + 120; control fimbria *n* = 6 P60 + 7, 4 P60 + 60, 6 P60 + 90 and 6 P60 + 120; *APP* fimbria *n* = 4 P60 + 7, 4 P60 + 60, 3 P60 + 90, and 3 P60 + 120. *p < 0.05, **p < 0.01 and ***p < 0.001 denote significant differences identified by Bonferroni's post hoc analyses. White arrows indicate YFP^+^ PDGFRα‐negative newborn oligodendrocytes. Scale bars represent 25 µm (c–j) or 33 µm (k–n) [Color figure can be viewed at wileyonlinelibrary.com]

To determine whether the ability of OPCs to generate new oligodendrocytes was affected by early amyloid pathology, we performed tamoxifen‐mediated cre‐lox lineage tracing of adult OPCs from P60, comparing oligodendrocyte generation in control (*Pdgfra‐CreER^T2^:: Rosa26‐YFP*) and *APP* (*Pdgfra‐CreER^T2^:: Rosa26‐YFP:: Pdgfb‐hAPP^Sw.Ind^*) mice (Figure 7). Coronal brain cryosections from P60 + 7 (P67), P60 + 60 (P120), P60 + 90 (P150), and P60 + 120 (P180) control and *APP* transgenic mice, containing the hippocampus, entorhinal cortex, or fimbria, were used for immunohistochemistry to detect YFP (green), PDGFRα (red), OLIG2 and Hoechst 33342 (blue) (Figure 7a–n). PDGFRα^+^ OLIG2^+^ YFP^+^ OPCs gave rise to new PDGFRα‐neg OLIG2^+^ YFP^+^ cells over time. As 97.1% ± 1.8% of YFP^+^ cells in the hippocampus of P60 + 120 control mice and 96.7% ± 2.0% of YFP^+^ cells in the hippocampus of P60 + 120 *APP* transgenic mice were OLIG2^+^ (mean ± *SD*, *n* = 3 mice per genotype; Figure S2), essentially all YFP^+^ cells were of the oligodendrocyte lineage.

OPCs differentiated to produce new PDGFRα‐neg YFP^+^ oligodendrocytes in the hippocampus (Figure 7a–f), entorhinal cortex (Figure 7g–j), fimbria (Figure 7a,b,k–n), and retrosplenial cortex (Figure S4) of adult control and *APP* mice, however by P60 + 90, significantly more YFP^+^ new oligodendrocytes had accumulated in the hippocampus of *APP* transgenic mice compared to controls (Figure 7o). By P60 + 120, the proportion of YFP^+^ cells that were new oligodendrocytes was also significantly higher in the entorhinal cortex (Figure 7p) and fimbria (Figure 7q) of *APP* transgenic mice compared to controls. This increase in cell addition resulted in an increase in the density of newborn YFP^+^ oligodendrocytes detected in the hippocampus (Figure 7r), entorhinal cortex (Figure 7s) and fimbria (Figure 7t) of *APP* mice relative to controls. To determine whether this corresponded to an increase in the number of newborn mature oligodendrocytes, we performed immunohistochemistry to detect YFP, PDGFRα and the mature oligodendrocyte marker, ASPA in the hippocampus and fimbria of P60 + 120 control and *APP* mice (Figure S3). We found that the density of YFP^+^ PDGFRα‐neg ASPA‐neg cells (presumably premyelinating oligodendrocytes) was equivalent in the hippocampus and fimbria of WT and *APP* mice (Figure S3). By contrast, the density of YFP^+^ PDGFRα‐neg ASPA^+^ mature oligodendrocytes was significantly increased in the hippocampus and fimbria of *APP* relative to control mice (Figure S3). In P60 + 120 *APP* mice, a similar increase in newborn mature oligodendrocyte density was also detected in the retrosplenial cortex (Figure S4), a region involved in learning and navigation (Vann, Aggleton, & Maguire, 2009), suggesting that oligodendrogenesis may be increased in a number of regions of the *APP* mouse brain.

**Figure 8:**
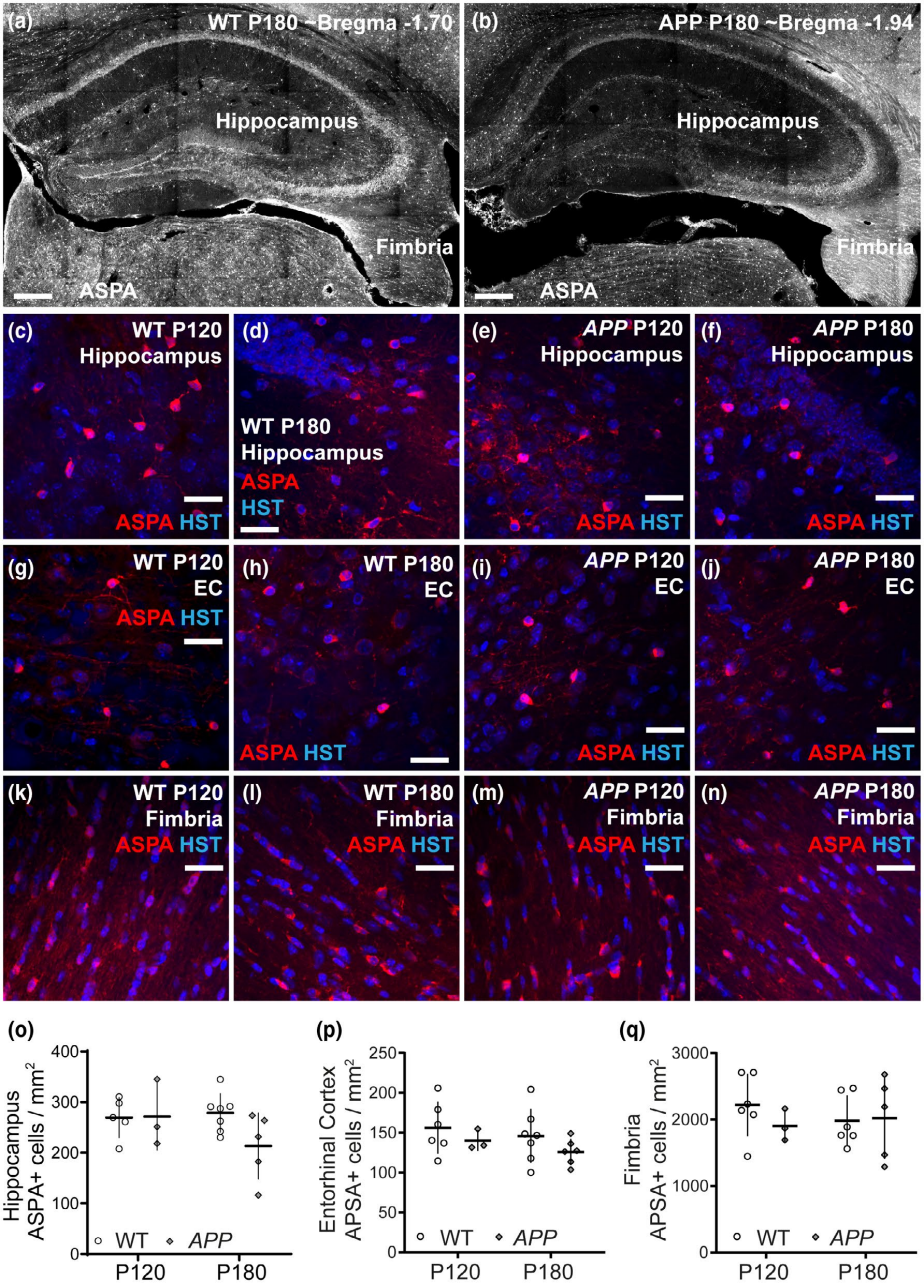
Oligodendrocyte number is normal in APP transgenic mice. (a–b) Low magnification confocal images of the hippocampus and fimbria in confocal brain sections from P180 WT and *APP* mice immunolabeled to detect ASPA. (c–f) Confocal images from the hippocampus in coronal brain cryosections (30 µm) from P120 and P180 WT and *APP* transgenic mice showing cells labeled with the oligodendrocyte marker ASPA (red) and Hoechst 33342 (blue). (g–j) Confocal images from the entorhinal cortex in coronal brain cryosections from P120 and P180 WT and *APP* transgenic mice showing cells labeled with the oligodendrocyte marker ASPA (red) and Hoechst 33342 (blue). (k–n) Confocal images from the fimbria in coronal brain cryosections from P120 and P180 WT and *APP* transgenic mice showing cells labeled with the oligodendrocyte marker ASPA (red) and Hoechst 33342 (blue). (o) Quantification of the density of ASPA^+^ oligodendrocytes in the hippocampus of WT (black bars, open circles) and *APP* (gray bars, gray diamonds) transgenic mice (oligodendrocytes per mm^2^ as adjusted for *x*–*y* area only) [Two‐way ANOVA, genotype: *F* (1, 16) = 1.78, *p* = 0.20; age: *F* (1, 16) = 1.07, *p* = 0.31; interaction: *F* (1, 16) = 2.01, *p* = 0.17]. (p) Quantification of the density of ASPA^+^ oligodendrocytes in the entorhinal cortex of WT and *APP* transgenic mice (oligodendrocytes per mm^2^ as adjusted for *x*–*y* area only) [Two‐way ANOVA, genotype: *F* (1, 18) = 2.08, *p* = 0.16; age: *F* (1, 18) = 0.95, *p* = 0.34; interaction: *F* (2, 53) = 0.02, *p* = 0.87]. (q) Quantification of the density of ASPA^+^ oligodendrocytes in the fimbria of WT and *APP* transgenic mice (oligodendrocytes per mm^2^ as adjusted for *x*–*y* area only) [Two‐way ANOVA, genotype: *F* (1, 16) = 0.40, *p* = 0.53; age: *F* (1, 16) = 0.08, *p* = 0.77; interaction: *F* (1, 16) = 0.64, *p* = 0.43]. Results are presented as mean ± *SD*. Hippocampus *n* = 5 P120 WT, 3 P120 APP, 7 P180 WT, and 5 P180 APP; entorhinal cortex *n* = 6 P120 WT, 3 P120 APP, 7 P180 WT, and 6 P180 APP; fimbria *n* = 6 P120 WT, 3 P120 APP, 6 P180 WT, and 5 P180 APP. Scale bars represent 30 µm [Color figure can be viewed at wileyonlinelibrary.com]

Surprisingly, the addition of new oligodendrocytes did not alter the total density of oligodendrocytes in the hippocampus, entorhinal cortex or fimbria of control or *APP* mice (Figure 8). By performing immunohistochemistry on coronal brain cryosections from P120 or P180 WT and *APP* transgenic mice to detect the mature oligodendrocyte marker ASPA (Figure 8a–n), we determined that the density of ASPA^+^ oligodendrocytes was higher in the fimbria (Figure 8q) and hippocampus (Figure 8o) than the entorhinal cortex (Figure 8p), but was consistent across age and between WT and *APP* transgenic mice. As microglia were previously shown to express ASPA in rat brain tissue (Madhavarao et al., 2004; Moffett et al., 2011), we also ensured that ASPA expression did not colocalize with the microglial marker Iba1 in WT or *APP* brain cryosections (Figure S5). As there are 213 ± 65 ASPA^+^ mature oligodendrocytes per mm^2^ in the hippocampus in P180 *APP* mice (Figure 8o; mean ± *SD*) and of these, 56.09 ± 4.5 per mm^2^ are adult‐born ASPA^+^ oligodendrocytes (Figure 7r; mean ± *SD*), ~26% of the mature ASPA^+^ oligodendrocytes were born after P60. By contrast, only ~10.6% of the mature ASPA^+^ oligodendrocytes present in the hippocampus of P180 WT mice were born after P60. Similarly, of the 2,018.2 ± 612.1 ASPA^+^ oligodendrocytes per mm^2^ of fimbria in P180 *APP* mice, 260.45 ± 28.69 per mm^2^ are adult born (mean ± *SD*), indicating that ~12.9% were born after P60. In WT mice ~5.0% of ASPA^+^ oligodendrocytes in the fimbria were born after P60. These data indicate that OPCs are active in *APP* mice and make a significant contribution to the total population of mature oligodendrocytes. These data may also suggest that amyloid pathology triggers oligodendrocyte death and replacement by P180, however, the total density of oligodendrocytes in the hippocampus of control and *APP* mice was variable. Considering this, it may not be possible to detect a difference in total mature oligodendrocyte density of <8% in the fimbria, between control and APP mice, or a difference of ~15% in total mature oligodendrocyte density in the hippocampus between WT and *APP* mice at P180, in the event that these cells were added to the population with no concurrent loss.

## 4 DISCUSSION

Herein we show that *APP* transgenic mice express an increased level of human APP in the hippocampus at P180, when they develop amyloid plaques (Figure 1). Behaviorally these mice were hyperactive by P60, which impacted their performance in other behavioral assessments, however, they did not develop spatial memory deficit by P180 (Figure 2). OPC density is normal in the hippocampus of *APP* transgenic mice, but by P100, they respond more robustly to GABA (Figures 3 and 4). Additionally, developmental myelination was affected in *APP* transgenic mice, as the nodes of Ranvier along hippocampal axons were shorter and the paranodes longer in young adulthood, and this phenotype was associated with increased myelin thickness (Figures 5 and 6). OPCs in the hippocampus, entorhinal cortex, and fimbria of young adult *APP* transgenic mice also produced a normal number of new oligodendrocytes, however, as pathology developed oligodendrogenesis increased (Figure 7). As this was not accompanied by a change in total oligodendrocyte number (Figure 8), we propose that *APP* transgenic mice have a higher level of oligodendrocyte turnover than their WT littermates by P180.

### 4.1 APP mice are hyperactive prior to amyloid plaque deposition

*APP* or J20 mice expressed a human variant of APP containing the Swedish (K670N/M671L) and Indiana (V717F) familial Alzheimer's disease linked mutations, in neurons and oligodendrocytes (Figure S1), driven by the *platelet‐derived growth factor‐beta chain* (*PDGF‐β*) promotor (Mucke et al., 2000). Both human APP (~100 kDa) and oligomerized amyloid β (~12 kDa) were present in the hippocampus of *APP* mice by P30, however, amyloid plaques did not form until P180 (Figure 1), consistent with previous reports (Meilandt et al., 2009; Mucke et al., 2000; Wright et al., 2013). The overexpression of human APP^SwInd^ was also associated with premature mortality (Figure 1; Cheng et al., 2007; Cissé et al., 2011; Dubal et al., 2015; Murakami et al., 2011; Verret et al., 2012), which has been previously attributed to their increased susceptibility to spontaneous seizures (Palop et al., 2007). As ~60% of *APP* mice died by P180, and it is reasonable to assume that the mice that died had developed more severe pathology, the reduced survival of *APP* mice unavoidably biased our analyses, skewing our characterization toward the less affected mice that survived to the older ages.

By subjecting WT and *APP* and mice to a battery of behavioral tasks at P60, P90, and P180, we determined that *APP* mice were hyperactive by P60, developed episodic working memory deficits by P90, and exhibited anxiety‐like behavioral traits but no spatial learning impairment by P180 (Figure 2). Most studies report that *APP* mice develop spatial learning deficits between 5 and 8 months of age (Cheng et al., 2007; Cissé et al., 2011; Flores et al., 2018; Harris et al., 2010; Mably et al., 2015; Mesquita et al., 2015; Sanchez et al., 2012: Wright et al., 2013), while others report no deficit in Barnes Maze performance before 12 months (Fujikawa et al., 2017; Nunes et al., 2015). By contrast, hyperactivity is a consistent behavioral feature of *APP* mice (Cheng et al., 2007; Cissé et al., 2011; Dubal et al., 2015; Flores et al., 2018; Fujikawa et al., 2017; Harris et al., 2010; Murakami et al., 2011; Sanchez et al., 2012; Verret et al., 2012; Wright et al., 2013), however, to our knowledge, we are the first to show a tendency toward increased anxiety‐like behavior in this strain. Most studies report a decrease in anxiety‐like behavior, or a disinhibition of caution in *APP* mice as early as 2 months of age (Cheng et al., 2007; Cissé et al., 2011; Dubal et al., 2015; Harris et al., 2010; Murakami et al., 2011; Sanchez et al., 2012) or no anxiety‐related phenotype (Dekens et al., 2018; Fujikawa et al., 2017; Wright et al., 2013). Mice expressing the Swedish (K670N/M671L), Iberian (I716F), and Artic (E693G) mutations in human *APP* (*APP^NL‐G‐F^* mice) simultaneously exhibit anxiogenic behavior in the open field (less time in the center) and anxiolytic behavior in the elevated plus maze (more time in the open arms) (Pervolaraki et al., 2019), which the authors suggest reflects altered decision‐making processes, rather than a core deficit in emotional motivation. The hyperactivity exhibited by our *APP* mice may underpin similar vagaries in decision‐making, and contribute to the apparent working memory deficit and anxiety‐like behavior in these mice.

Altered OPC and/or oligodendrocyte function could also contribute to behavioral abnormalities in *APP* mice, as both cell types influence neural circuit function, memory, and affective behavior (reviewed Pepper, Pitman, Cullen, & Young, 2018). The ablation of OPCs has been shown to produce anxiety‐like behavior within 7 days (Birey et al., 2015), and while the electrophysiological response of OPC is altered by P100 in *APP* mice, any reduction detected in OPC density is minor, and *APP* mice do not display an anxiety‐like phenotype until P180. Similarly, increased hippocampal myelin thickness and the onset of working memory deficits are detected early in *APP* mice, however, in other contexts, thick myelin enhances hippocampal‐dependent memory formation (Jeffries et al., 2016), making it unlikely that this cellular change impairs working memory performance, but this cannot be entirely ruled out within this pathological context.

### 4.2 OPCs from P100 APP transgenic mice have a heightened response to GABA

OPCs in the hippocampus of P100 *APP* mice responded more robustly to the bath application of GABA than those in control mice; this could reflect a change in GABA_A_ receptor expression, or a change in the conductance of GABA_A_ receptors. APP is a synaptic protein that can regulate GABAergic signaling, predominantly by modulating presynaptic metabotropic GABA_B_ receptors or the reversal potential of chloride (reviewed by Tang, 2019). Experimentally manipulating the expression of APP decreases expression of the chloride transporter, KCC2 (K^+^‐Cl^‐^ cotransporter 2; *SLC12A5)*, which alters the effect of GABA_A_ receptor activation on neuronal membrane potential (Chen et al., 2017; Doshina et al., 2017) and modulates GABA_A_ receptor subunit expression (Chen et al., 2017). In hippocampal neuron cultures from *APP* knockout mice, the amplitude of evoked unitary inhibitory post‐synaptic currents is reduced, as is their response to the GABA_A_ receptor agonist, isoguvacine (Chen et al., 2017). This phenotype was associated with a ~50% reduction in expression of the α1 GABA_A_ receptor subunit in the hippocampus of *APP* knockout mice, that could be rescued by potentiating KCC2 function (Chen et al., 2017). OPCs express few α1‐containing GABA_A_ receptors (Lin & Bergles, 2004), but, it is possible that the increased response to GABA detected in OPCs in the hippocampus of P100 *APP* mice is the result of an APP/KCC2 mediated increase in the expression of α1 or other GABA_A_ receptor subunits. However, human APP was not expressed by OPCs in our mice, and it is unclear whether exogenous APP can alter KCC2 and/or GABA_A_ receptor subunit expression within these cells.

APP also interacts with the presynaptic GABA_B1a_ subunit of GABA_B_ receptors to alter receptor trafficking, and *APP* deletion reduces the presynaptic GABA_B_‐mediated inhibition of neurotransmitter release (Dinamarca et al., 2019). Secreted APP can also modulate neurotransmission by binding to presynaptic GABA_B_ receptors (Rice et al., 2019). It is possible that APP‐mediated changes to presynaptic GABA_B_ signaling could affect neuron‐OPC communication and OPC receptor expression, as OPCs can modify their expression of AMPA receptors in response to altered neuronal activity (Ge et al., 2006). OPCs have also been shown to express GABA_B_ receptors (Luyt et al., 2007; Luyt, Varadi, & Molnar, 2003; Serrano‐Regal et al., 2019), however, the currents evoked in OPCs by GABA application were completely antagonized by a selective GABA_A_ receptor antagonist, excluding the direct involvement of OPC GABA_B_ receptors in the heightened response to GABA.

A change in the response of OPCs to GABA may also be the consequence of the dysfunctional glutamatergic signaling associated with Alzheimer's disease (Findley, Bartke, Hascup, & Hascup, 2019). GABA_A_ activation on OPCs is considered to be excitatory (Lin & Bergles, 2004), however, the activation of GABA_A_ receptors can still negatively modulate glutamatergic signaling by increasing membrane conductance (shunting) and/or altering intracellular chloride concentration. The bath application of GABA can significantly reduce the response of hippocampal OPCs to KA (Lin & Bergles, 2004), indicating that a heightened OPC GABA_A_ response could serve to dampen pathological glutamatergic signaling onto OPCs. How this might affect OPC behavior is unclear. The activation of GABA_A_ receptors in rat OPCs cultures has no effect on OPC differentiation or myelin protein expression (MBP or MAG; Serrano‐Regal et al., 2019). By contrast, applying the GABA_A_ receptor antagonist, GABAzine, to developmental mouse organotypic cortical slice cultures increased the number of OPCs and oligodendrocytes present at 6 days in vitro (Hamilton et al., 2017). This effect of GABAzine on oligodendrocyte lineage cell number was prevented by blocking neuronal action potentials with tetrodotoxin (TTX), suggesting that the effect of GABAzine was dependent on the release of an activity‐dependent factor from neurons (Hamilton et al., 2017). As neither GABAzine nor the GABA_A_ agonist muscimol altered node of Ranvier length in mouse organotypic cortical slice cultures (Hamilton et al., 2017; Zonouzi et al., 2015) it is likely that the changes detected in node length in our study were independent of the increased responsiveness of OPCs to GABA, additionally, the impact that GABAergic signaling has on myelin thickness has not been explored.

### 4.3 Amyloid accumulation changes myelin ultrastructure

We have shown that paranodes are longer and nodes of Ranvier shorter for axons in the hippocampus of 3‐month‐old *APP* transgenic mice, compared with WT controls. Paranodes form at the end of each myelin internode, flanking the node of Ranvier, facilitated by contact‐mediated signaling between proteins in the myelin sheath and the axon to maintain the clustered voltage‐gated sodium channels at the nodes of Ranvier and tether the myelin internode to the axon (reviewed by Pepper et al., 2018). The effect of amyloid pathology on node of Ranvier length does not appear to be the result of altered axon diameter, as axon diameter was similar in WT and *APP* transgenic mice. Furthermore, we found no correlation between node of Ranvier length and node diameter (a proxy for axon diameter) in WT or *APP* transgenic mice [linear regression node length v node width deviation of slope from 0: WT slope = 0.04, *F* (1, 227) = 2.2, *p* = 0.14; *APP* slope = 0.04, *F* (1, 140), *p* = 0.22], a finding that is consistent with a previous data showing that node of Ranvier length and diameter did not correlate in the optic nerve or fronto‐parietal motor cortex of Sprague–Dawley rats (Arancibia‐Cárcamo et al., 2017). However, APP can be found at the nodes of Ranvier (Xu et al., 2014) and can increase NaV1.6‐mediated sodium currents (Li et al., 2016), making it possible that pathological human APP could directly induce narrowing of nodes of Ranvier in the hippocampus. In the 3xTg transgenic mouse line, a qualitative decrease was noted in NaV1.6 expression in the CA1 region of the hippocampus and the entorhinal cortex of 6‐month‐old mice (Desai et al., 2009), which could be the result of myelin degradation and an overall reduction in node of Ranvier number, but could also reflect a change in nodal structure.

It is likely that the increased paranode length measured for hippocampal axons in the *APP* transgenic mice is the direct result of *APP* transgenic mice having thicker myelin. On average, oligodendrocytes that myelinated axons within the hippocampus of *APP* mice produced an extra myelin lamella, relative to those in WT mice, which is consistent with previous reports of increased myelin thickness in the hippocampus of 2‐month‐old *APP/PSEN1* transgenic mice (Wu et al., 2017). The extra layer of myelin must be anchored at the paranode, and could readily explain the increased paranode length, and perhaps, if the myelin encroaches on the node of Ranvier, the decrease in node of Ranvier length.

### 4.4 Amyloid accumulation increases oligodendrocyte turnover

OPCs generate new oligodendrocytes in the gray and white matter of the adult mouse brain (Dimou, Simon, Kirchhoff, Takebayashi, & Gotz, 2008; Fukushima et al., 2015; Hill, Patel, Medved, Reiss, & Nishiyama, 2013; Kang, Fukaya, Yang, Rothstein, & Bergles, 2010; Rivers et al., 2008; Young et al., 2013), and while the rate of OPC proliferation and oligodendrocyte addition slows with aging (reviewed by Wang & Young, 2014), OPCs can rapidly proliferate and differentiate in response to a demyelinating event, to facilitate the replacement of lost oligodendrocytes and enable remyelination (Assinck et al., 2017; Baxi et al., 2017; Tripathi, Rivers, Young, Jamen, & Richardson, 2010; Zawadzka et al., 2010). By performing cre‐lox lineage tracing using *Pdgfrα‐CreER^T2^:: Rosa26‐YFP* transgenic mice, we have shown that the number of new mature oligodendrocytes that accumulate in the hippocampus, entorhinal cortex, and fimbria of *APP* transgenic mice between P60 and P180 is significantly higher than the number added to these regions in the WT mouse brain. In the hippocampus, oligodendrocyte addition started to deviate between WT and *APP* transgenic mice between 4 and 5 months of age. However, the effect of amyloid pathology on oligodendrogenesis was delayed in the entorhinal cortex and fimbria, being seen between 5 and 6 months of age. Despite the increased addition of new mature oligodendrocytes to the brain of *APP* transgenic mice, total oligodendrocyte density in the hippocampus, entorhinal cortex and fimbria was equivalent in WT and *APP* mice, suggesting that the large number of new cells may serve to replace oligodendrocytes that are lost, rather than increasing total oligodendrocyte number.

Myelin abnormalities have been reported for a number of animal models of amyloid pathology (Chu et al., 2017; Tse et al., 2018), including focal myelin loss associated with amyloid plaques (Mitew et al., 2010; Schmued, Raymick, Paule, Dumas, & Sarkar, 2013). We found that the proportion of hippocampal axons that were myelinated in 3‐month‐old WT and *APP* transgenic mice was equivalent, however, the marked increase in oligodendrogenesis observed between 4 and 6 months of age in the *APP* transgenic mice may suggest that oligodendrocyte loss and myelination are a later feature of the pathogenesis. This time line differs significantly from that of *APP^Sw^* mice in which myelin loss was not reported until 18 months of age (Tse et al., 2018), but would be consistent with the marked reduction in myelinated axon number in the hippocampus of 6‐month‐old 3xTg mice, which occurred prior to plaque deposition (Desai et al., 2009), and the myelin aberrations detected in *APP/PS1* mice at 6 months, coincident with plaque detection (Behrendt et al., 2013).

Understanding the cellular changes that occur in the early stages of Alzheimer's disease will enable improved early detection and the development of therapies to halt disease progression. We have determined that early amyloid pathology affects OPCs and myelin morphology, and that as pathology develops there is an increase in oligodendrogenesis without an accompanying change in total oligodendrocyte number, suggesting that new oligodendrocytes are produced to replace lost oligodendrocytes. Our findings contribute to the growing body of literature suggesting that OPCs represent a viable target to protect against early degeneration of the white matter tracts in Alzheimer's disease.

## DECLARATION OF TRANSPARENCY

The authors, reviewers and editors affirm that in accordance to the policies set by the *Journal of Neuroscience Research*, this manuscript presents an accurate and transparent account of the study being reported and that all critical details describing the methods and results are present.

## CONFLICT OF INTEREST

The authors have no conflict of interest to declare.

## AUTHOR CONTRIBUTIONS

*Conceptualization,* K.M.Y. and C.L.C.; *Methodology,* K.M.Y. and C.L.C.; *Formal Analysis,* S.F., K.A.P., C.L.C., and K.M.Y.; *Data Curation,* S.F., K.A.P., N.B., S.W., B.S.S., and K.M.Y.; *Writing – Original Draft,* S.F., K.M.Y., K.A.P., and C.L.C.; *Writing – Review & Editing,* S.F., K.M.Y., K.A.P., and C.L.C.; *Supervision,* K.M.Y., K.A.P., and C.L.C.; *Project Administration,* K.M.Y. and C.L.C.; *Funding Acquisition,* K.M.Y., K.A.P., and C.L.C.

## Supporting information


**Figure S1**. Human APP is expressed in hippocampal neurons and oligodendrocytes in APP mice. (a–b) Low magnification confocal images showing the hippocampus and fimbria of P180 WT and *APP* mice following immunohistochemistry to detect human APP (hAPP; green) and Hoechst 33342 (HST; blue). (c–d) Confocal images show cells in the dentate gyrus (DG) of the hippocampus in P180 WT and *APP* mice that express hAPP (green), the microtubule associated protein 2 (MAP2, red; stains neuronal dendrites and cell bodies) and/or Hoechst 33342 (HST; blue). hAPP was not detected in any cells within the DG of WT mice (c) but was highly expressed by dentate granule neurons (open arrowheads) and mossy cells (solid arrowheads) in the DG of *APP* mice (d). (e–f) Confocal images show cells in the CA1 region of the hippocampus in P180 WT and *APP* mice that express hAPP (green), MAP2 (red) and/or HST (blue). hAPP was not detected in cells within the CA1 of WT mice (e) but was highly expressed by pyramidal neurons (double arrowheads) in *APP* mice. (g–h) Confocal images of cells within the CA3 region of the hippocampus (g) or fimbria (h) in P180 *APP* mice that are labelled with hAPP (green), PDGFRα (red) and/or ASPA (blue). hAPP was expressed by CA3 pyramidal neurons (g) and all ASPA+ oligodendrocytes (solid yellow arrowheads), but no OPCs (open yellow arrowheads) in the hippocampus. *amyloid plaque. Scale bars represent 200 μm (a–b) or 20 μm (c–h)
**Figure S2**. Essentially all YFP‐labelled cells are OLIG2+ in P60 + 120 control and APP mice. P60 control (*Pdgfrα‐CreERT2 :: Rosa26‐YFP*) and *APP* (*Pdgfrα‐CreERT2 :: Rosa26‐YFP :: Pdgfb‐hAPPSwInd*) mice received tamoxifen to initiate the cre‐mediated YFP‐labelling of OPCs and their progeny. Tissue was analysed histologically 120 days later (P60 + 120) at P180. (a–b) Low magnification confocal image showing the hippocampus and fimbria in coronal brain cryosections from P60 + 120 control and *APP* transgenic mice stained to detect YFP (green), OLIG2 (red) and Hoechst 33342 (HST, blue). (c–e) Confocal image showing cells in the hippocampus of P60 + 120 control mice that express YFP (green), OLIG2 (red) and HST (blue). (f–h) Confocal image showing cells in the hippocampus of P60 + 120 *APP* transgenic mice that express YFP (green), OLIG2 (red) and HST (blue). (i–k) Confocal image showing cells in the fimbria of P60 + 120 *APP* mice that express YFP (green), OLIG2 (red) and HST (blue). White arrows indicate YFP+ OLIG2+ cells. White arrow head shows an example of a rare YFP+ OLIG2‐negative cell. Scale bar represents 280 μm (a–b), 50 μm (c–h), or 25 μm (i–k)
**Figure S3**. The density of mature (ASPA+) newborn oligodendrocytes is increased in the hippocampus and fimbria of P60 + 120 APP mice. P60 control (*Pdgfrα‐CreERT2 :: Rosa26‐YFP*) and *APP* (*Pdgfrα‐CreERT2 :: Rosa26‐YFP :: Pdgfb‐hAPPSwInd*) mice received tamoxifen to initiate the cre‐mediated YFP‐labelling of OPCs and their progeny. Tissue was analysed histologically 120 days later (P60 + 120) at P180. (a–d) Confocal image of cells in the hippocampus of a P60 + 120 control mouse that express YFP (green), ASPA (red) and/or PDGFRα (blue). Panels show the fluorescent overlay and each channel separately. (e–h) Confocal image of cells in the hippocampus of a P60 + 120 *APP* mouse that express YFP (green), ASPA (red) and/or PDGFRα (blue). Panels show the fluorescent overlay and each channel separately. (i) Quantification of the density of: (i) newborn oligodendrocytes (YFP+ PDGFRα‐neg), newborn immature oligodendrocytes (PDGFRα‐neg, ASPA‐neg) or newborn mature oligodendrocytes (PDGFRα‐neg ASPA+) in the hippocampus of P60 + 120 control or *APP* mice [Two‐way ANOVA, genotype: *F* (1, 15) = 49.33, *p* < 0.001; cell type: *F* (2, 15) = 80.29, *p* < 0.001; interaction: *F* (2, 15) = 8.83, *p* < 0.003; *n* = 4 control and *n* = 3 *APP* mice]. (j) Quantification of the density of: (i) newborn oligodendrocytes (YFP+ PDGFRα‐neg), newborn immature oligodendrocytes (PDGFRα‐neg, ASPA‐neg) or newborn mature oligodendrocytes (PDGFRα‐neg ASPA+) in the fimbria of P60 + 120 control or *APP* mice [Two‐way ANOVA, genotype: *F* (1, 15) = 81.97, *p* < 0.001; cell type: *F* (2, 15) = 96.15, *p* < 0.001; interaction: *F* (2, 15) = 21.21, *p* < 0.001; *n* = 4 control and *n* = 3 *APP* mice). ***p < 0.001 denotes significance from Bonferroni post‐hoc analyses. White arrow heads indicate PDGFRα+ OPCs that are YFP‐labelled; yellow arrow head indicate PDGFRα‐neg YFP+ ASPA‐neg presumptive immature (premyelinating) oligodendrocytes; white arrows indicate YFP+ ASPA+ newborn mature oligodendrocytes. Scale bars represent 35 μm
**Figure S4**. Oligodendrogenesis is increased in the retrosplenial cortex of P60 + 120 APP mice. P60 control (*Pdgfrα‐CreERT2 :: Rosa26‐YFP*) and *APP* (*Pdgfrα‐CreERT2 :: Rosa26‐YFP :: Pdgfb‐hAPPSwInd*) mice received tamoxifen to initiate the cre‐mediated YFP‐labelling of OPCs and their progeny. Tissue was analysed histologically 120 days later (P60 + 120) at P180. (a–b) Low magnification confocal images show the retrosplenial granular cortex and the retrosplenial dysgranular cortex (counted together as the retrosplenial cortex) in coronal cryosections from P60 + 120 control or *APP* mice following immunohistochemistry to detect YFP (green), ASPA (red) and PDGFRα (blue). (c–f) Confocal images of cells in the retrosplenial cortex of a P60 + 120 control mouse that express YFP (green), ASPA (red) and/or PDGFRα (blue). Panels show the fluorescent overlay and each channel separately. (g–j) Confocal image of cells in the retrosplenial cortex of a P60 + 120 *APP* mouse that express YFP (green), ASPA (red) and/or PDGFRα (blue). Panels show the fluorescent overlay and each channel separately. (k) Quantification of the density of PDGFRα+ OPCs in the retrosplenial cortex of P60 + 120 control or *APP* mice [Two‐tailed, unpaired *t*‐test, *t* (5) = 2.80; *n* = 4 control and *n* = 3 *APP* mice]. (l) Quantification of the density of: (i) newborn oligodendrocytes (YFP+ PDGFRα‐neg), newborn immature oligodendrocytes (PDGFRα‐neg, ASPA‐neg) or newborn mature oligodendrocytes (PDGFRα‐neg ASPA+) in the retrosplenial cortex of P60 + 120 control or *APP* mice [Two‐way ANOVA, genotype: *F* (1, 18) = 58.04, *p* < 0.001; cell type: *F* (2, 18) = 58.84, *p* < 0.001; interaction: *F* (2, 18) = 12.37, *p* < 0.001; *n* = 4 control and *n* = 3 *APP* mice]. *p < 0.05 and ***p < 0.001 denote the significance of the unpaired *t*‐test or Bonferroni post‐hoc test. Blue arrows indicate PDGFRα+ OPCs that are YFP‐labelled; yellow arrows indicate YFP+ ASPA+ newborn mature oligodendrocytes. Scale bars represent 120 μm (a–b) or 35 μm (c–j)
**Figure S5**. APSA+ cells in the hippocampus of P180 WT or APP mice do not express the microglial marker Iba1. (a–b) Low magnification confocal images showing the hippocampus and fimbria in coronal brain cryosections (30 μm) from P180 WT and *APP* mice following immunohistochemistry to detect the microglial marker Iba1 (green), the mature oligodendrocyte marker ASPA (red) and the nuclear label Hoechst 33342 (HST, blue). We determined that 0 of 1672 ASPA+ cells analysed in WT mice (*n* = 4) and 0 of the 993 ASPA+ cells analysed in APP mice (*n* = 3) co‐labelled for Iba1. (c–f) Confocal images showing cells within the hippocampus of WT and *APP* mice that label for Iba1 or ASPA, demonstrating that individual cells do not co‐express these markers. Green arrows denote Iba1+ microglia and red arrows denote ASPA+ mature oligodendrocytes. (g–l) Confocal images showing examples of closely apposed Iba1+ microglia and ASPA+ mature oligodendrocytes (2 closely overlapping nuclei and cells of distinct shapes) within the hippocampus of WT and *APP* mice (accounts for ~2% of all APSA+ cells). Scale bars represent 280 μm (a–b) or 10 μm (c–1)
**Table S1**. List of antibodies used in this research

Transparent Science Questionnaire for Authors

Transparent Peer Review Report

## Data Availability

The data that support the findings of this study will be made available by the corresponding authors upon reasonable request.

## References

[jnr24672-bib-0001] Arancibia‐Cárcamo, I. L. , Ford, M. C. , Cossell, L. , Ishida, K. , Tohyama, K. , & Attwell, D. (2017). Node of Ranvier length as a potential regulator of myelinated axon conduction speed. Elife, 6, 1–15. 10.7554/eLife.23329 PMC5313058 28130923

[jnr24672-bib-0002] Assinck, P. , Duncan, G. J. , Plemel, J. R. , Lee, M. J. , Stratton, J. A. , Manesh, S. B. , … Tetzlaff, W. (2017). Myelinogenic plasticity of oligodendrocyte precursor cells following spinal cord contusion injury. Journal of Neuroscience, 37(36), 8635–8654. 10.1523/jneurosci.2409-16.2017 28760862 PMC6596672

[jnr24672-bib-0003] Attar, A. , Liu, T. , Chan, W.‐T.‐ C. , Hayes, J. , Nejad, M. , Lei, K. , & Bitan, G. (2013). A shortened Barnes maze protocol reveals memory deficits at 4‐months of age in the triple‐transgenic mouse model of Alzheimer's disease. PLoS One, 8(11), e80355 10.1371/journal.pone.0080355 24236177 PMC3827415

[jnr24672-bib-0004] Auderset, L. , Cullen, C. L. , & Young, K. M. (2016). Low density lipoprotein‐receptor related protein 1 is differentially expressed by neuronal and glial populations in the developing and mature mouse central nervous system. PLoS One, 11(6), e0155878 10.1371/journal.pone.0155878 27280679 PMC4900551

[jnr24672-bib-0005] Baxi, E. G. , DeBruin, J. , Jin, J. , Strasburger, H. J. , Smith, M. D. , Orthmann‐Murphy, J. L. , … Calabresi, P. A. (2017). Lineage tracing reveals dynamic changes in oligodendrocyte precursor cells following cuprizone‐induced demyelination. Glia, 65(12), 2087–2098. 10.1002/glia.23229 28940645 PMC5761347

[jnr24672-bib-0006] Behrendt, G. , Baer, K. , Buffo, A. , Curtis, M. A. , Faull, R. L. , Rees, M. I. , … Dimou, L. (2013). Dynamic changes in myelin aberrations and oligodendrocyte generation in chronic amyloidosis in mice and men. Glia, 61(2), 273–286. 10.1002/glia.22432 23090919

[jnr24672-bib-0007] Benitez, A. , Fieremans, E. , Jensen, J. H. , Falangola, M. F. , Tabesh, A. , Ferris, S. H. , & Helpern, J. A. (2014). White matter tract integrity metrics reflect the vulnerability of late‐myelinating tracts in Alzheimer's disease. NeuroImage. Clinical, 4, 64–71. 10.1016/j.nicl.2013.11.001 24319654 PMC3853114

[jnr24672-bib-0008] Birey, F. , Kloc, M. , Chavali, M. , Hussein, I. , Wilson, M. , Christoffel, D. J. , … Aguirre, A. (2015). Genetic and stress‐induced loss of NG2 glia triggers emergence of depressive‐like behaviors through reduced secretion of FGF2. Neuron, 88(5), 941–956. 10.1016/j.neuron.2015.10.046 26606998 PMC5354631

[jnr24672-bib-0009] Borges, K. , & Kettenmann, H. (1995). Blockade of K+ channels induced by AMPA/kainate receptor activation in mouse oligodendrocyte precursor cells is mediated by NA+ entry. Journal of Neuroscience Research, 42(4), 579–593. 10.1002/jnr.490420416 8568944

[jnr24672-bib-0010] Braak, H. , & Braak, E. (1996). Development of Alzheimer‐related neurofibrillary changes in the neocortex inversely recapitulates cortical myelogenesis. Acta Neuropathologica, 92(2), 197–201. 10.1007/s004010050508 8841666

[jnr24672-bib-0011] Brueggen, K. , Dyrba, M. , Cardenas‐Blanco, A. , Schneider, A. , Fliessbach, K. , Buerger, K. , … Teipel, S. J. (2019). Structural integrity in subjective cognitive decline, mild cognitive impairment and Alzheimer's disease based on multicenter diffusion tensor imaging. Journal of Neurology, 266(10), 2465–2474. 10.1007/s00415-019-09429-3 31227891

[jnr24672-bib-0012] Burgold, S. , Bittner, T. , Dorostkar, M. M. , Kieser, D. , Fuhrmann, M. , Mitteregger, G. , … Herms, J. (2011). In vivo multiphoton imaging reveals gradual growth of newborn amyloid plaques over weeks. Acta Neuropathologica, 121(3), 327–335. 10.1007/s00401-010-0787-6 21136067 PMC3038220

[jnr24672-bib-0013] Bussière, T. , Bard, F. , Barbour, R. , Grajeda, H. , Guido, T. , Khan, K. , … Buttini, M. (2004). Morphological characterization of Thioflavin‐S‐positive amyloid plaques in transgenic Alzheimer mice and effect of passive Aβ immunotherapy on their clearance. American Journal of Pathology, 165(3), 987–995. 10.1016/S0002-9440(10)63360-3 15331422 PMC1618604

[jnr24672-bib-0014] Carasatorre, M. , Ochoa‐Alvarez, A. , Velázquez‐Campos, G. , Lozano‐Flores, C. , Díaz‐Cintra, S. Y. , & Ramírez‐Amaya, V. (2015). Hippocampal synaptic expansion induced by spatial experience in rats correlates with improved information processing in the hippocampus. PLoS One, 10(8), e0132676 10.1371/journal.pone.0132676 26244549 PMC4526663

[jnr24672-bib-0015] Charlton, R. A. , Barrick, T. R. , McIntyre, D. J. , Shen, Y. , O'Sullivan, M. , Howe, F. A. , … Markus, H. S. (2006). White matter damage on diffusion tensor imaging correlates with age‐related cognitive decline. Neurology, 66(2), 217–222. 10.1212/01.wnl.0000194256.15247.83 16434657

[jnr24672-bib-0016] Chen, M. , Wang, J. , Jiang, J. , Zheng, X. , Justice, N. J. , Wang, K. , … Yang, L. (2017). APP modulates KCC2 expression and function in hippocampal GABAergic inhibition. Elife, 6, 1–26. 10.7554/eLife.20142 PMC5224924 28054918

[jnr24672-bib-0017] Cheng, I. H. , Scearce‐Levie, K. , Legleiter, J. , Palop, J. J. , Gerstein, H. , Bien‐Ly, N. , … Mucke, L. (2007). Accelerating amyloid‐β fibrillization reduces oligomer levels and functional deficits in Alzheimer disease mouse models. Journal of Biological Chemistry, 282(33), 23818–23828. 10.1074/jbc.M701078200 17548355

[jnr24672-bib-0018] Choi, S. J. , Lim, K. O. , Monteiro, I. , & Reisberg, B. (2005). Diffusion tensor imaging of frontal white matter microstructure in early Alzheimer's disease: A preliminary study. Journal of Geriatric Psychiatry and Neurology, 18(1), 12–19. 10.1177/0891988704271763 15681623

[jnr24672-bib-0019] Chopra, S. , Shaw, M. , Shaw, T. , Sachdev, P. S. , Anstey, K. J. , & Cherbuin, N. (2018). More highly myelinated white matter tracts are associated with faster processing speed in healthy adults. NeuroImage, 171(August), 332–340. 10.1016/j.neuroimage.2017.12.069 29274747

[jnr24672-bib-0020] Chu, T. H. , Cummins, K. , Sparling, J. S. , Tsutsui, S. , Brideau, C. , Nilsson, K. P. R. , … Stys, P. K. (2017). Axonal and myelinic pathology in 5xFAD Alzheimer's mouse spinal cord. PLoS One, 12(11), 1–22. 10.1371/journal.pone.0188218 PMC5703477 29176903

[jnr24672-bib-0021] Cissé, M. , Sanchez, P. E. , Kim, D. H. , Ho, K. , Yu, G.‐Q. , & Mucke, L. (2011). Ablation of cellular prion protein does not ameliorate abnormal neural network activity or cognitive dysfunction in the J20 line of human amyloid precursor protein transgenic mice. Journal of Neuroscience, 31(29), 10427–10431. 10.1523/JNEUROSCI.1459-11.2011 21775587 PMC3314063

[jnr24672-bib-0022] Clarke, L. E. , Young, K. M. , Hamilton, N. B. , Li, H. , Richardson, W. D. , & Attwell, D. (2012). Properties and fate of oligodendrocyte progenitor cells in the corpus callosum, motor cortex, and piriform cortex of the mouse. Journal of Neuroscience, 32(24), 8173–8185. 10.1523/JNEUROSCI.0928-12.2012 22699898 PMC3378033

[jnr24672-bib-0023] Collins, J. M. , King, A. E. , Woodhouse, A. , Kirkcaldie, M. T. K. , & Vickers, J. C. (2015). The effect of focal brain injury on beta‐amyloid plaque deposition, inflammation and synapses in the APP/PS1 mouse model of Alzheimer's disease. Experimental Neurology, 267, 219–229. 10.1016/j.expneurol.2015.02.034 25747037

[jnr24672-bib-0024] Deacon, R. M. J. , & Rawlins, J. N. P. (2006). T‐maze alternation in the rodent. Nature Protocols, 1(1), 7–12. 10.1038/nprot.2006.2 17406205

[jnr24672-bib-0025] Dekens, D. W. , Naudé, P. J. W. , Keijser, J. N. , Boerema, A. S. , De Deyn, P. P. , & Eisel, U. L. M. (2018). Lipocalin 2 contributes to brain iron dysregulation but does not affect cognition, plaque load, and glial activation in the J20 Alzheimer mouse model. Journal of Neuroinflammation, 15(1), 330 10.1186/s12974-018-1372-5 30501637 PMC6267886

[jnr24672-bib-0026] Desai, M. K. , Guercio, B. J. , Narrow, W. C. , & Bowers, W. J. (2011). An Alzheimer's disease‐relevant presenilin‐1 mutation augments amyloid‐beta‐induced oligodendrocyte dysfunction. Glia, 59(4), 627–640. 10.1002/glia.21131 21294162 PMC3045399

[jnr24672-bib-0027] Desai, M. K. , Mastrangelo, M. A. , Ryan, D. A. , Sudol, K. L. , Narrow, W. C. , & Bowers, W. J. (2010). Early oligodendrocyte/myelin pathology in Alzheimer's disease mice constitutes a novel therapeutic target. American Journal of Pathology, 177(3), 1422–1435. 10.2353/ajpath.2010.100087 20696774 PMC2928974

[jnr24672-bib-0028] Desai, M. K. , Sudol, K. L. , Janelsins, M. C. , Mastrangelo, M. A. , Frazer, M. E. , & Bowers, W. J. (2009). Triple‐transgenic Alzheimer's disease mice exhibit region‐specific abnormalities in brain myelination patterns prior to appearance of amyloid and tau pathology. Glia, 57(1), 54–65. 10.1002/glia.20734 18661556 PMC2584762

[jnr24672-bib-0029] Dimou, L. , Simon, C. , Kirchhoff, F. , Takebayashi, H. , & Gotz, M. (2008). Progeny of olig2‐expressing progenitors in the gray and white matter of the adult mouse cerebral cortex. Journal of Neuroscience, 28(41), 10434–10442. 10.1523/JNEUROSCI.2831-08.2008 18842903 PMC6671038

[jnr24672-bib-0030] Dinamarca, M. C. , Raveh, A. , Schneider, A. , Fritzius, T. , Früh, S. , Rem, P. D. , … Bettler, B. (2019). Complex formation of APP with GABA B receptors links axonal trafficking to amyloidogenic processing. Nature Communications, 10(1), 1–17. 10.1038/s41467-019-09164-3 PMC6430795 30902970

[jnr24672-bib-0031] Doshina, A. , Gourgue, F. , Onizuka, M. , Opsomer, R. , Wang, P. , Ando, K. , … Pierrot, N. (2017). Cortical cells reveal APP as a new player in the regulation of GABAergic neurotransmission. Scientific Reports, 7, 370 10.1038/s41598-017-00325-2 28337033 PMC5428293

[jnr24672-bib-0032] Du, A. T. , Schu, N. V. , Amend, D. , Laakso, M. P. , Hsu, Y. Y. , Jagust, W. J. , … Street, C. (2001). Magnetic resonance imaging of the entorhinal cortex and hippocampus in mild cognitive impairment and Alzheimer's disease. Journal of Neurology, Neurosurgery, and Psychiatry, 71(4), 441–447. 10.1136/jnnp.71.4.441 PMC1763497 11561025

[jnr24672-bib-0033] Dubal, D. B. , Zhu, L. , Sanchez, P. E. , Worden, K. , Broestl, L. , Johnson, E. , … Mucke, L. (2015). Life extension factor klotho prevents mortality and enhances cognition in hAPP transgenic mice. Journal of Neuroscience, 35(6), 2358–2371. 10.1523/JNEUROSCI.5791-12.2015 25673831 PMC4323521

[jnr24672-bib-0034] Fan, Y. T. , Fang, Y. W. , Chen, Y. P. , Leshikar, E. D. , Lin, C. P. , Tzeng, O. J. L. , … Huang, C. M. (2019). Aging, cognition, and the brain: Effects of age‐related variation in white matter integrity on neuropsychological function. Aging and Mental Health, 23(7), 831–839. 10.1080/13607863.2018.1455804 29634290

[jnr24672-bib-0035] Ferreira, S. , Pitman, K. A. , Young, K. M. , Cullen, C. L. , Summers, B. S. , & Wang, S. (2020). Oligodendrogenesis increases in hippocampal grey and white matter prior to locomotor or memory impairment in an adult mouse model of tauopathy. European Journal of Neuroscience, 1–23. 10.1111/ejn.14726 32181929 PMC8451881

[jnr24672-bib-0036] Findley, C. A. , Bartke, A. , Hascup, K. N. , & Hascup, E. R. (2019). Amyloid beta‐related alterations to glutamate signaling dynamics during Alzheimer's disease progression. ASN Neuro, 11 10.1177/1759091419855541 PMC6582288 31213067

[jnr24672-bib-0037] Flores, J. , Noël, A. , Foveau, B. , Lynham, J. , Lecrux, C. , & LeBlanc, A. C. (2018). Caspase‐1 inhibition alleviates cognitive impairment and neuropathology in an Alzheimer's disease mouse model. Nature Communications, 9, 3916 10.1038/s41467-018-06449-x PMC6156230 30254377

[jnr24672-bib-0038] Franklin, K. B. J. , & Paxinos, G. (2007). The mouse brain in stereotaxic coordinates (3rd ed.). Amsterdam, the Netherlands: Elsevier.

[jnr24672-bib-0039] Fujikawa, R. , Higuchi, S. , Nakatsuji, M. , Yasui, M. , Ikedo, T. , Nagata, M. , … Minami, M. (2017). Deficiency in EP4 receptor‐associated protein ameliorates abnormal anxiety‐like behavior and brain inflammation in a mouse model of Alzheimer disease. American Journal of Pathology, 187(8), 1848–1854. 10.1016/j.ajpath.2017.04.010 28624505

[jnr24672-bib-0040] Fukushima, S. , Nishikawa, K. , Furube, E. , Muneoka, S. , Ono, K. , Takebayashi, H. , & Miyata, S. (2015). Oligodendrogenesis in the fornix of adult mouse brain; the effect of LPS‐induced inflammatory stimulation. Brain Research, 1627, 52–69. 10.1016/j.brainres.2015.09.011 26385416

[jnr24672-bib-0041] Gawel, K. , Gibula, E. , Marszalek‐Grabska, M. , Filarowska, J. , & Kotlinska, J. H. (2019). Assessment of spatial learning and memory in the Barnes maze task in rodents—Methodological consideration. Naunyn‐Schmiedeberg's Archives of Pharmacology, 392(1), 1–18. 10.1007/s00210-018-1589-y PMC6311199 30470917

[jnr24672-bib-0042] Ge, W.‐P. , Yang, X.‐J. , Zhang, Z. , Wang, H.‐K. , Shen, W. , Deng, Q.‐D. , & Duan, S. (2006). Long‐term potentiation of neuron‐glia synapses mediated by Ca2+‐permeable AMPA receptors. Science, 312(5779), 1533–1537. 10.1126/science.1124669 16763153

[jnr24672-bib-0043] Goedert, M. , Spillantini, M. G. , Jakes, R. , Rutherford, D. , & Crowther, R. A. (1989). Multiple isoforms of human microtubule‐associated protein tau: Sequences and localization in neurofibrillary tangles of Alzheimer's disease. Neuron, 3(4), 519–526. 10.1016/0896-6273(89)90210-9 2484340

[jnr24672-bib-0044] Gold, B. T. , Zhu, Z. , Brown, C. A. , Andersen, A. H. , LaDu, M. J. , Tai, L. , … Smith, C. D. (2014). White matter integrity is associated with cerebrospinal fluid markers of Alzheimer's disease in normal adults. Neurobiology of Aging, 35(10), 2263–2271. 10.1016/j.neurobiolaging.2014.04.030 24866404 PMC4087077

[jnr24672-bib-0045] Grant, M. K. O. , Handoko, M. , Rozga, M. , Brinkmalm, G. , Portelius, E. , Blennow, K. , … Liu, P. (2019). Human cerebrospinal fluid 6E10‐ immunoreactive protein species contain amyloid precursor protein fragments. PLoS One, 14(2), 1–23. 10.1371/journal.pone.0212815 PMC6394962 30817799

[jnr24672-bib-0046] Grimmer, T. , Henriksen, G. , Wester, H.‐J. , Förstl, H. , Klunk, W. E. , Mathis, C. A. , … Drzezga, A. (2009). Clinical severity of Alzheimer's disease is associated with PIB uptake in PET. Neurobiology of Aging, 30(12), 1902–1909. 10.1016/j.neurobiolaging.2008.01.016 18346821

[jnr24672-bib-0047] Griner, S. L. , Seidler, P. , Bowler, J. , Murray, K. A. , Yang, T. P. , Sahay, S. , … Eisenberg, D. S. (2019). Structure based inhibitors of Amyloid Beta core suggest a common interface with Tau. ELife, 8(310), e46924 10.7554/eLife.46924 31612856 PMC6850776

[jnr24672-bib-0048] Hamilton, N. B. , Clarke, L. E. , Arancibia‐Carcamo, I. L. , Kougioumtzidou, E. , Matthey, M. , Káradóttir, R. , … Attwell, D. (2017). Endogenous GABA controls oligodendrocyte lineage cell number, myelination, and CNS internode length. Glia, 65(2), 309–321. 10.1002/glia.23093 27796063 PMC5214060

[jnr24672-bib-0049] Hamilton, T. G. , Klinghoffer, R. A. , Corrin, P. D. , & Soriano, P. (2003). Evolutionary divergence of platelet‐derived growth factor alpha receptor signaling mechanisms. Molecular and Cellular Biology, 23(11), 4013–4025. Retrieved from http://www.ncbi.nlm.nih.gov/pubmed/12748302 12748302 10.1128/MCB.23.11.4013-4025.2003 PMC155222

[jnr24672-bib-0050] Harris, J. A. , Devidze, N. , Halabisky, B. , Lo, I. , Thwin, M. T. , Yu, G.‐Q. , … Mucke, L. (2010). Many neuronal and behavioral impairments in transgenic mouse models of Alzheimer's disease are independent of caspase cleavage of the amyloid precursor protein. Journal of Neuroscience, 30(1), 372–381. 10.1523/JNEUROSCI.5341-09.2010 20053918 PMC3064502

[jnr24672-bib-0051] Hill, R. A. , Patel, K. D. , Medved, J. , Reiss, A. M. , & Nishiyama, A. (2013). NG2 cells in white matter but not gray matter proliferate in response to PDGF. Journal of Neuroscience, 33(36), 14558–14566. 10.1523/JNEUROSCI.2001-12.2013 24005306 PMC3761056

[jnr24672-bib-0052] Hirsiger, S. , Koppelmans, V. , Mérillat, S. , Erdin, C. , Narkhede, A. , Brickman, A. M. , & Jäncke, L. (2017). Executive functions in healthy older adults are differentially related to macro‐ and microstructural white matter characteristics of the cerebral lobes. Frontiers in Aging Neuroscience, 9(November), 1–14. 10.3389/fnagi.2017.00373 29249957 PMC5715235

[jnr24672-bib-0053] Horiuchi, M. , Maezawa, I. , Itoh, A. , Wakayama, K. , Jin, L.‐W. , Itoh, T. , & DeCarli, C. (2012). Amyloid β1–42 oligomer inhibits myelin sheet formation in vitro. Neurobiology of Aging, 338(3), 499–509. 10.1016/j.neurobiolaging.2010.05.007 PMC3013291 20594620

[jnr24672-bib-0054] Howell, O. W. , Palser, A. , Polito, A. , Melrose, S. , Zonta, B. , Scheiermann, C. , … Reynolds, R. (2006). Disruption of neurofascin localization reveals early changes preceding demyelination and remyelination in multiple sclerosis. Brain, 129(12), 3173–3185. 10.1093/brain/awl290 17041241

[jnr24672-bib-0055] Iseki, E. , Yamamoto, R. , Murayama, N. , Minegishi, M. , Togo, T. , Katsuse, O. , … Arai, H. (2006). Immunohistochemical investigation of neurofibrillary tangles and their tau isoforms in brains of limbic neurofibrillary tangle dementia. Neuroscience Letters, 405(1–2), 29–33. 10.1016/j.neulet.2006.06.036 16859829

[jnr24672-bib-0056] Jeffries, M. A. , Urbanek, K. , Torres, L. , Wendell, S. G. , Rubio, M. E. , & Fyffe‐Maricich, S. L. (2016). ERK1/2 activation in preexisting oligodendrocytes of adult mice drives new myelin synthesis and enhanced CNS function. Journal of Neuroscience, 36(35), 9186–9200. 10.1523/JNEUROSCI.1444-16.2016 27581459 PMC5005725

[jnr24672-bib-0057] Jin, J. , & Maren, S. (2015). Prefrontal‐hippocampal interactions in memory and emotion. Frontiers in Systems Neuroscience, 9, 170 10.3389/fnsys.2015.00170 26696844 PMC4678200

[jnr24672-bib-0058] Kalani, A. , Chaturvedi, P. , Maldonado, C. , Bauer, P. , Joshua, I. G. , Tyagi, S. C. , & Tyagi, N. (2017). Dementia‐like pathology in type‐2 diabetes: A novel microRNA mechanism. Molecular and Cellular Neuroscience, 80, 58–65. 10.1016/j.mcn.2017.02.005 28219659 PMC5432966

[jnr24672-bib-0059] Kang, S. H. , Fukaya, M. , Yang, J. K. , Rothstein, J. D. , & Bergles, D. E. (2010). NG2+ CNS glial progenitors remain committed to the oligodendrocyte lineage in postnatal life and following neurodegeneration. Neuron, 68(4), 668–681. 10.1016/j.neuron.2010.09.009 21092857 PMC2989827

[jnr24672-bib-0060] Kim, Y. S. , Woo, J. , Lee, C. J. , & Yoon, B. E. (2017). Decreased glial GABA and tonic inhibition in cerebellum of mouse model for attention‐deficit/hyperactivity disorder (ADHD). Experimental Neurobiology, 26(4), 206–212. 10.5607/en.2017.26.4.206 28912643 PMC5597551

[jnr24672-bib-0061] Lee, J.‐T. , Xu, J. , Lee, J.‐M. , Ku, G. , Han, X. , Yang, D.‐I. , … Hsu, C. Y. (2004). Amyloid‐β peptide induces oligodendrocyte death by activating the neutral sphingomyelinase–ceramide pathway. Journal of Cell Biology, 164(1), 123–131. 10.1083/jcb.200307017 14709545 PMC2171973

[jnr24672-bib-0062] Lee, S. , Viqar, F. , Zimmerman, M. E. , Narkhede, A. , Tosto, G. , Benzinger, T. L. S. , … Brickman, A. M. (2016). White matter hyperintensities are a core feature of Alzheimer's disease: Evidence from the dominantly inherited Alzheimer network. Annals of Neurology, 79(6), 929–939. 10.1002/ana.24647 27016429 PMC4884146

[jnr24672-bib-0063] Li, S. , Wang, X. , Ma, Q.‐H. , Yang, W. , Zhang, X.‐G. , Dawe, G. S. , & Xiao, Z.‐C. (2016). Amyloid precursor protein modulates Nav1.6 sodium channel currents through a Go‐coupled JNK pathway. Scientific Reports, 6(1), 39320 10.1038/srep39320 28008944 PMC5180232

[jnr24672-bib-0064] Lin, S. , & Bergles, D. E. (2004). Synaptic signaling between GABAergic interneurons and oligodendrocyte precursor cells in the hippocampus. Nature Neuroscience, 7(1), 24–32. 10.1038/nn1162 14661022

[jnr24672-bib-0065] Luyt, K. , Slade, T. P. , Dorward, J. J. , Durant, C. F. , Wu, Y. , Shigemoto, R. , … Molnár, E. (2007). Developing oligodendrocytes express functional GABA B receptors that stimulate cell proliferation and migration. Journal of Neurochemistry, 100(3), 822–840. 10.1111/j.1471-4159.2006.04255.x 17144904

[jnr24672-bib-0066] Luyt, K. , Varadi, A. , & Molnar, E. (2003). Functional metabotropic glutamate receptors are expressed in oligodendrocyte progenitor cells. Journal of Neurochemistry, 84(6), 1452–1464. 10.1046/j.1471-4159.2003.01661.x 12614345

[jnr24672-bib-0067] Ma, B. F. , Xie, M. J. , & Zhou, M. (2012). Bicarbonate efflux via GABAA receptors depolarizes membrane potential and inhibits two‐pore domain potassium channels of astrocytes in rat hippocampal slices. Glia, 60(11), 1761–1772. 10.1002/glia.22395 22855415 PMC3901573

[jnr24672-bib-0068] Mably, A. J. , Liu, W. , Mc Donald, J. M. , Dodart, J.‐C. , Bard, F. , Lemere, C. A. , … Walsh, D. M. (2015). Anti‐Aβ antibodies incapable of reducing cerebral Aβ oligomers fail to attenuate spatial reference memory deficits in J20 mice. Neurobiology of Disease, 82, 372–384. 10.1016/j.nbd.2015.07.008 26215784 PMC4641028

[jnr24672-bib-0069] Madhavarao, C. N. , Moffett, J. R. , Moore, R. A. , Viola, R. E. , Namboodiri, M. A. A. , & Jacobowitz, D. M. (2004). Immunohistochemical localization of aspartoacylase in the rat central nervous system. Journal of Comparative Neurology, 472(3), 318–329. 10.1002/cne.20080 15065127

[jnr24672-bib-0070] Meilandt, W. J. , Cisse, M. , Ho, K. , Wu, T. , Esposito, L. A. , Scearce‐Levie, K. , … Mucke, L. (2009). Neprilysin overexpression inhibits plaque formation but fails to reduce pathogenic A oligomers and associated cognitive deficits in human amyloid precursor protein transgenic mice. Journal of Neuroscience, 29(7), 1977–1986. 10.1523/jneurosci.2984-08.2009 19228952 PMC2768427

[jnr24672-bib-0071] Mesquita, S. D. , Ferreira, A. C. , Gao, F. , Coppola, G. , Geschwind, D. H. , Sousa, J. C. , … Marques, F. (2015). The choroid plexus transcriptome reveals changes in type I and II interferon responses in a mouse model of Alzheimer's disease. Brain, Behavior, and Immunity, 49(June), 280–292. 10.1016/j.bbi.2015.06.008 26092102

[jnr24672-bib-0072] Miller, D. L. , Papayannopoulos, I. A. , Styles, J. , Bobin, S. A. , Lin, Y. Y. , Biemann, K. , & Iqbal, K. (1993). Peptide compositions of the cerebrovascular and senile plaque core amyloid deposits of Alzheimer's disease. Archives of Biochemistry and Biophysics, 301(1), 41–52. 10.1006/abbi.1993.1112 8442665

[jnr24672-bib-0073] Mitew, S. , Kirkcaldie, M. T. K. , Halliday, G. M. , Shepherd, C. E. , Vickers, J. C. , & Dickson, T. C. (2010). Focal demyelination in Alzheimer's disease and transgenic mouse models. Acta Neuropathologica, 119(5), 567–577. 10.1007/s00401-010-0657-2 20198482

[jnr24672-bib-0074] Moffett, J. R. , Arun, P. , Ariyannur, P. S. , Garbern, J. Y. , Jacobowitz, D. M. , & Namboodiri, A. M. A. (2011). Extensive aspartoacylase expression in the rat central nervous system. Glia, 59(10), 1414–1434. 10.1002/glia.21186 21598311 PMC3143213

[jnr24672-bib-0075] Montarolo, F. , Martire, S. , Perga, S. , Spadaro, M. , Brescia, I. , Allegra, S. , … Bertolotto, A. (2019). NURR1 deficiency is associated to ADHD‐like phenotypes in mice. Translational Psychiatry, 9(1), 207 10.1038/s41398-019-0544-0 31455763 PMC6712038

[jnr24672-bib-0076] Mucke, L. , Masliah, E. , Yu, G.‐Q. , Mallory, M. , Rockenstein, E. M. , Tatsuno, G. , … McConlogue, L. (2000). High‐level neuronal expression of Aβ 1–42 in wild‐type human amyloid protein precursor transgenic mice: Synaptotoxicity without plaque formation. Journal of Neuroscience, 20(11), 4050–4058. 10.1523/JNEUROSCI.20-11-04050.2000 10818140 PMC6772621

[jnr24672-bib-0077] Murakami, K. , Yokoyama, S. , Murata, N. , Ozawa, Y. , Irie, K. , Shirasawa, T. , & Shimizu, T. (2011). Insulin receptor mutation results in insulin resistance and hyperinsulinemia but does not exacerbate Alzheimer's‐like phenotypes in mice. Biochemical and Biophysical Research Communications, 409(1), 34–39. 10.1016/j.bbrc.2011.04.101 21549686

[jnr24672-bib-0078] Newman, M. , Wilson, L. , Verdile, G. , Lim, A. , Khan, I. , Hani, S. , … Lardelli, M. (2014). Differential , dominant activation and inhibition of Notch signalling and APP cleavage by truncations of PSEN1 in human disease. Human Molecular Genetics, 23(3), 602–617. 10.1093/hmg/ddt448 24101600

[jnr24672-bib-0079] Nunes, M. A. , Schöwe, N. M. , Monteiro‐Silva, K. C. , Baraldi‐Tornisielo, T. , Souza, S. I. G. , Balthazar, J. , … Buck, H. S. (2015). Chronic microdose lithium treatment prevented memory loss and neurohistopathological changes in a transgenic mouse model of Alzheimer's disease. PLoS One, 10(11), 1–26. 10.1371/journal.pone.0142267 PMC4659557 26605788

[jnr24672-bib-0080] O'Dwyer, L. , Lamberton, F. , Bokde, A. L. W. , Ewers, M. , Faluyi, Y. O. , Tanner, C. , … Hampel, H. (2011). Multiple indices of diffusion identifies white matter damage in mild cognitive impairment and Alzheimer's disease. PLoS One, 6(6), e21745 10.1371/journal.pone.0021745 21738785 PMC3128090

[jnr24672-bib-0081] O'Rourke, M. , Cullen, C. L. , Auderset, L. , Pitman, K. A. , Achatz, D. , Gasperini, R. , & Young, K. M. (2016). Evaluating tissue‐specific recombination in a Pdgfra‐CreER T2 transgenic mouse line. PLoS One, 11(9), 1–19. 10.1371/journal.pone.0162858 PMC5023134 27626928

[jnr24672-bib-0082] Palop, J. J. , Chin, J. , Roberson, E. D. , Wang, J. , Thwin, M. T. , Bien‐Ly, N. , … Mucke, L. (2007). Aberrant excitatory neuronal activity and compensatory remodeling of inhibitory hippocampal circuits in mouse models of Alzheimer's disease. Neuron, 55(5), 697–711. 10.1016/j.neuron.2007.07.025 17785178 PMC8055171

[jnr24672-bib-0083] Pennanen, C. , Kivipelto, M. , Tuomainen, S. , Hartikainen, P. , Hänninen, T. , Laakso, M. P. , … Soininen, H. (2004). Hippocampus and entorhinal cortex in mild cognitive impairment and early AD. Neurobiology of Aging, 25(3), 303–310. 10.1016/S0197-4580(03)00084-8 15123335

[jnr24672-bib-0084] Pepper, R. E. , Pitman, K. A. , Cullen, C. L. , & Young, K. M. (2018). How do cells of the oligodendrocyte lineage affect neuronal circuits to influence motor function, memory and mood? Frontiers in Cellular Neuroscience, 12(November), 399 10.3389/fncel.2018.00399 30524235 PMC6262292

[jnr24672-bib-0085] Pervolaraki, E. , Hall, S. P. , Foresteire, D. , Saito, T. , Saido, T. C. , Whittington, M. A. , … Dachtler, J. (2019). Insoluble Aβ overexpression in an App knock‐in mouse model alters microstructure and gamma oscillations in the prefrontal cortex, affecting anxiety‐related behaviours. Disease Models & Mechanisms, 12(9), dmm040550 10.1242/dmm.040550 31439589 PMC6765200

[jnr24672-bib-0086] Pu, D. , Zhao, Y. , Chen, J. , Sun, Y. , Lv, A. , Zhu, S. , … Xiao, Q. (2018). Protective effects of sulforaphane on cognitive impairments and AD‐like lesions in diabetic mice are associated with the upregulation of Nrf2 transcription activity. Neuroscience, 381, 35–45. 10.1016/j.neuroscience.2018.04.017 29684505

[jnr24672-bib-0087] Racine, A. M. , Adluru, N. , Alexander, A. L. , Christian, B. T. , Okonkwo, O. C. , Oh, J. , … Johnson, S. C. (2014). Associations between white matter microstructure and amyloid burden in preclinical Alzheimer's disease: A multimodal imaging investigation. NeuroImage: Clinical, 4, 604–614. 10.1016/j.nicl.2014.02.001 24936411 PMC4053642

[jnr24672-bib-0088] Radde, R. , Bolmont, T. , Kaeser, S. A. , Coomaraswamy, J. , Lindau, D. , Stoltze, L. , … Jucker, M. (2006). Aβ42‐driven cerebral amyloidosis in transgenic mice reveals early and robust pathology. EMBO Reports, 7(9), 940–946. 10.1038/sj.embor.7400784 16906128 PMC1559665

[jnr24672-bib-0089] Rice, H. C. , De Malmazet, D. , Schreurs, A. , Frere, S. , Van Molle, I. , Volkov, A. N. , … De Wit, J. (2019). Secreted amyloid‐b precursor protein functions as a GABA B R1a ligand to modulate synaptic transmission. Science, 363(6423), eaao4827 10.1126/science.aao4827 30630900 PMC6366617

[jnr24672-bib-0090] Rivers, L. E. , Young, K. M. , Rizzi, M. , Jamen, F. , Psachoulia, K. , Wade, A. , … Richardson, W. D. (2008). PDGFRA/NG2 glia generate myelinating oligodendrocytes and piriform projection neurons in adult mice. Nature Neuroscience, 11(12), 1392–1401. 10.1038/nn.2220 18849983 PMC3842596

[jnr24672-bib-0091] Roher, A. E. , Lowenson, J. D. , Clarke, S. , Wolkow, C. , Wang, R. , Cotter, R. J. , … Greenberg, B. D. (1993). Structural alterations in the peptide backbone of β‐amyloid core protein may account for its deposition and stability in Alzheimer's disease. Journal of Biological Chemistry, 268(5), 3072–3083. 8428986

[jnr24672-bib-0092] Sanchez, P. E. , Zhu, L. , Verret, L. , Vossel, K. A. , Orr, A. G. , Cirrito, J. R. , … Mucke, L. (2012). Levetiracetam suppresses neuronal network dysfunction and reverses synaptic and cognitive deficits in an Alzheimer's disease model. Proceedings of the National Academy of Sciences, 109(42), E2895–E2903. 10.1073/pnas.1121081109 PMC3479491 22869752

[jnr24672-bib-0093] Schmidt, M. L. , Lee, V.‐ M.‐Y. , & Trojanowski, J. Q. (1990). Relative abundance of tau and neurofilament epitopes in hippocampal neurofibrillary tangles. American Journal of Pathology, 136(5), 1069–1075. 1693468 PMC1877428

[jnr24672-bib-0094] Schmued, L. C. , Raymick, J. , Paule, M. G. , Dumas, M. , & Sarkar, S. (2013). Characterization of myelin pathology in the hippocampal complex of a transgenic mouse model of Alzheimer's disease. Current Alzheimer Research, 10(1), 30–37. 10.2174/1567205011310010005 23157338

[jnr24672-bib-0095] Scott, J. A. , Braskie, M. N. , Tosun, D. , Thompson, P. M. , Weiner, M. , DeCarli, C. , & Carmichael, O. T. (2015). Cerebral amyloid and hypertension are independently associated with white matter lesions in elderly. Frontiers in Aging Neuroscience, 7(December), 1–9. 10.3389/fnagi.2015.00221 26648866 PMC4664630

[jnr24672-bib-0096] Selkoe, D. J. , & Hardy, J. (2016). The amyloid hypothesis of Alzheimer's disease at 25 years. EMBO Molecular Medicine, 8(6), 595–608. 10.15252/emmm.201606210 27025652 PMC4888851

[jnr24672-bib-0097] Serrano‐Regal, M. P. , Luengas‐Escuza, I. , Bayón‐Cordero, L. , Ibarra‐Aizpurua, N. , Alberdi, E. , Pérez‐Samartín, A. , … Sánchez‐Gómez, M. V. (2019). Oligodendrocyte differentiation and myelination is potentiated via GABAB receptor activation. Neuroscience, in press. 10.1016/j.neuroscience.2019.07.014 31349008

[jnr24672-bib-0098] Shi, L. , Zhao, L. , Wong, A. , Wang, D. , & Mok, V. (2015). Mapping the relationship of contributing factors for preclinical Alzheimer's disease. Scientific Reports, 5, 1–9. 10.1038/srep11259 PMC4507140 26190794

[jnr24672-bib-0099] Simons, M. , & Nave, K.‐A. (2016). Oligodendrocytes: Myelination and axonal support. Cold Spring Harbor Perspectives in Biology, 8(1), a020479 10.1101/cshperspect.a020479 PMC4691794 26101081

[jnr24672-bib-0100] Snaidero, N. , Möbius, W. , Czopka, T. , Hekking, L. H. P. , Mathisen, C. , Verkleij, D. , … Simons, M. (2014). Myelin membrane wrapping of CNS axons by PI(3,4,5)P3‐dependent polarized growth at the inner tongue. Cell, 156(1–2), 277–290. 10.1016/j.cell.2013.11.044 24439382 PMC4862569

[jnr24672-bib-0101] Snowden, S. G. , Ebshiana, A. A. , Hye, A. , Pletnikova, O. , O'Brien, R. , Yang, A. , … Thambisetty, M. (2019). Neurotransmitter imbalance in the brain and Alzheimer's disease pathology. Journal of Alzheimer's Disease, 72(1), 35–43. 10.3233/jad-190577 PMC12961646 31561368

[jnr24672-bib-0102] Srinivas, S. , Watanabe, T. , Lin, C. S. , William, C. M. , Tanabe, Y. , Jessell, T. M. , & Costantini, F. (2001). Cre reporter strains produced by targeted insertion of EYFP and ECFP into the ROSA26 locus. BMC Developmental Biology, 1, 4 Retrieved from http://www.ncbi.nlm.nih.gov/pubmed/11299042 11299042 10.1186/1471-213X-1-4 PMC31338

[jnr24672-bib-0103] Stassart, R. M. , Möbius, W. , Nave, K.‐A. , & Edgar, J. M. (2018). The axon‐myelin unit in development and degenerative disease. Frontiers in Neuroscience, 12(July), 467 10.3389/fnins.2018.00467 30050403 PMC6050401

[jnr24672-bib-0104] Stojic, A. , Bojcevski, J. , Williams, S. K. , Diem, R. , & Fairless, R. (2018). Early nodal and paranodal disruption in autoimmune optic neuritis. Journal of Neuropathology and Experimental Neurology, 77(5), 361–373. 10.1093/jnen/nly011 29444299

[jnr24672-bib-0105] Stricker, N. H. , Schweinsburg, B. C. , Delano‐Wood, L. , Wierenga, C. E. , Bangen, K. J. , Haaland, K. Y. , … Bondi, M. W. (2009). Decreased white matter integrity in late‐myelinating fiber pathways in Alzheimer's disease supports retrogenesis. NeuroImage, 45(1), 10–16. 10.1016/j.neuroimage.2008.11.027 19100839 PMC2782417

[jnr24672-bib-0106] Sullivan, G. M. , & Feinn, R. (2012). Using effect size‐or why the P value is not enough. Journal of Graduate Medical Education, 4(3), 279–282. 10.4300/JGME-D-12-00156.1 23997866 PMC3444174

[jnr24672-bib-0107] Sun, A. , Nguyen, X. V. , & Bing, G. (2002). Comparative analysis of an improved thioflavin‐S stain, Gallyas silver stain, and immunohistochemistry for neurofibrillary tangle demonstration on the same sections. Journal of Histochemistry and Cytochemistry, 50(4), 463–472. 10.1177/002215540205000403 11897799

[jnr24672-bib-0108] Tang, B. L. (2019). Amyloid precursor protein (APP) and GABAergic neurotransmission. Cells, 8(6), 550 10.3390/cells8060550 PMC6627941 31174368

[jnr24672-bib-0109] Tripathi, R. B. , Rivers, L. E. , Young, K. M. , Jamen, F. , & Richardson, W. D. (2010). NG2 glia generate new oligodendrocytes but few astrocytes in a murine experimental autoimmune encephalomyelitis model of demyelinating disease. Journal of Neuroscience, 30(48), 16383–16390. 10.1523/JNEUROSCI.3411-10.2010 21123584 PMC3063541

[jnr24672-bib-0110] Tse, K.‐H. , Cheng, A. , Ma, F. , & Herrup, K. (2018). DNA damage‐associated oligodendrocyte degeneration precedes amyloid pathology and contributes to Alzheimer's disease and dementia. Alzheimer's & Dementia, 14(5), 664–679. 10.1016/j.jalz.2017.11.010 PMC5938117 29328926

[jnr24672-bib-0111] Vann, S. D. , Aggleton, J. P. , & Maguire, E. A. (2009). What does the retrosplenial cortex do? Nature Reviews Neuroscience, 10(11), 792–802. 10.1038/nrn2733 19812579

[jnr24672-bib-0112] Verret, L. , Mann, E. O. , Hang, G. B. , Barth, A. M. I. , Cobos, I. , Ho, K. , … Palop, J. J. (2012). Inhibitory interneuron deficit links altered network activity and cognitive dysfunction in Alzheimer model. Cell, 149(3), 708–721. 10.1016/j.cell.2012.02.046 22541439 PMC3375906

[jnr24672-bib-0113] Wang, H. , Li, C. , Wang, H. , Mei, F. , Liu, Z. , Shen, H. Y. , & Xiao, L. (2013). Cuprizone‐induced demyelination in mice: Age‐related vulnerability and exploratory behavior deficit. Neuroscience Bulletin, 29(2), 251–259. 10.1007/s12264-013-1323-1 23558591 PMC5561883

[jnr24672-bib-0114] Wang, S. , & Young, K. M. (2014). White matter plasticity in adulthood. Neuroscience, 276, 148–160. 10.1016/j.neuroscience.2013.10.018 24161723

[jnr24672-bib-0115] Wright, A. L. , Zinn, R. , Hohensinn, B. , Konen, L. M. , Beynon, S. B. , Tan, R. P. , … Vissel, B. (2013). Neuroinflammation and neuronal loss precede Aβ plaque deposition in the hAPP‐J20 mouse model of Alzheimer's disease. PLoS One, 8(4), e59586 10.1371/journal.pone.0059586 23560052 PMC3613362

[jnr24672-bib-0116] Wu, Y. , Ma, Y. , Liu, Z. , Geng, Q. , Chen, Z. , & Zhang, Y. (2017). Alterations of myelin morphology and oligodendrocyte development in early stage of Alzheimer's disease mouse model. Neuroscience Letters, 642, 102–106. 10.1016/j.neulet.2017.02.007 28174059

[jnr24672-bib-0117] Wyss, J. M. , Swanson, L. W. , & Cowan, W. M. (1980). The organization of the fimbria, dorsal fomix and ventral hippocampal commissure in the rat. Anatomy and Embryology, 158(3), 303–306. 10.1007/bf00301819 7356182

[jnr24672-bib-0118] Xu, D.‐E. , Zhang, W.‐M. , Yang, Z. Z. , Zhu, H.‐M. , Yan, K. , Li, S. , … Xiao, Z.‐C. (2014). Amyloid precursor protein at node of Ranvier modulates nodal formation. Cell Adhesion & Migration, 8(4), 396–403. 10.4161/cam.28802 25482638 PMC4594432

[jnr24672-bib-0119] Xu, J. , Chen, S. , Ahmed, S. H. , Chen, H. , Ku, G. , Goldberg, M. P. , & Hsu, C. Y. (2001). Amyloid‐beta peptides are cytotoxic to oligodendrocytes. Journal of Neuroscience, 21(1), RC118 10.1523/JNEUROSCI.21-01-j0001.2001 11150354 PMC6762453

[jnr24672-bib-0120] Yoshiyama, Y. , Higuchi, M. , Zhang, B. , Huang, S.‐M. , Iwata, N. , Saido, T. C. , … Lee, V.‐ M.‐Y. (2007). Synapse loss and microglial activation precede tangles in a P301S tauopathy mouse model. Neuron, 53(3), 337–351. 10.1016/j.neuron.2007.01.010 17270732

[jnr24672-bib-0121] Young, K. M. , Psachoulia, K. , Tripathi, R. B. , Dunn, S.‐J. , Cossell, L. , Attwell, D. , … Richardson, W. D. (2013). Oligodendrocyte dynamics in the healthy adult CNS: Evidence for myelin remodeling. Neuron, 77(5), 873–885. 10.1016/j.neuron.2013.01.006 23473318 PMC3842597

[jnr24672-bib-0122] Zawadzka, M. , Rivers, L. E. , Fancy, S. P. J. , Zhao, C. , Tripathi, R. , Jamen, F. , … Franklin, R. J. M. (2010). CNS‐resident glial progenitor/stem cells produce Schwann cells as well as oligodendrocytes during repair of CNS demyelination. Cell Stem Cell, 6(6), 578–590. 10.1016/j.stem.2010.04.002 20569695 PMC3856868

[jnr24672-bib-0123] Zhang, Y. U. , Schuff, N. , Du, A.‐T. , Rosen, H. J. , Kramer, J. H. , Gorno‐Tempini, M. L. , … Weiner, M. W. (2009). White matter damage in frontotemporal dementia and Alzheimer's disease measured by diffusion MRI. Brain, 132(9), 2579–2592. 10.1093/brain/awp071 19439421 PMC2732263

[jnr24672-bib-0124] Zhang, Y. , Tadesse, A. , Gurfein, B. T. , Zameer, A. , Snyder, B. J. , Ge, C. , … Cre, O. (2009). Notch1 signaling plays a role in regulating precursor differentiation during CNS remyelination. Proceedings of the National Academy of Sciences of the United States of America, 106(45), 19162–19167. 10.1073/pnas.0902834106 19855010 PMC2776461

[jnr24672-bib-0125] Zonouzi, M. , Scafidi, J. , Li, P. , McEllin, B. , Edwards, J. , Dupree, J. L. , … Gallo, V. (2015). GABAergic regulation of cerebellar NG2 cell development is altered in perinatal white matter injury. Nature Neuroscience, 18(5), 674–682. 10.1038/nn.3990 25821912 PMC4459267

