# Supplementary material for: Amyloidosis is associated with thicker myelin and increased oligodendrogenesis in the adult mouse brain
Source: J Neurosci Res. 2020 Jun 18;98(10):1905–32. doi: 10.1002/jnr.24672 (PMC7540704; doi:10.1002/jnr.24672)
Supplement: Supplementary file 1 — Figure S1. Human APP is expressed in hippocampal neurons and oligodendrocytes in APP mice. (a–b) Low magnification confocal images showing the hippocampus and fimbria of P180 WT and APP mice following immunohistochemistry to detect human APP (hAPP; green) and Hoechst 33342 (HST; blue). (c–d) Confocal images show cells in the dentate gyrus (DG) of the hippocampus in P180 WT and APP mice that express hAPP (green), the microtubule associated protein 2 (MAP2, red; stains neuronal dendrites and cell bodies) and/or Hoechst 33342 (HST; blue). hAPP was not detected in any cells within the DG of WT mice (c) but was highly expressed by dentate granule neurons (open arrowheads) and mossy cells (solid arrowheads) in the DG of APP mice (d). (e–f) Confocal images show cells in the CA1 region of the hippocampus in P180 WT and APP mice that express hAPP (green), MAP2 (red) and/or HST (blue). hAPP was not detected in cells within the CA1 of WT mice (e) but was highly expressed by pyramidal neurons (double arrowheads) in APP mice. (g–h) Confocal images of cells within the CA3 region of the hippocampus (g) or fimbria (h) in P180 APP mice that are labelled with hAPP (green), PDGFRα (red) and/or ASPA (blue). hAPP was expressed by CA3 pyramidal neurons (g) and all ASPA+ oligodendrocytes (solid yellow arrowheads), but no OPCs (open yellow arrowheads) in the hippocampus. *amyloid plaque. Scale bars represent 200 μm (a–b) or 20 μm (c–h) Figure S2. Essentially all YFP‐labelled cells are OLIG2+ in P60 + 120 control and APP mice. P60 control (Pdgfrα‐CreERT2 :: Rosa26‐YFP) and APP (Pdgfrα‐CreERT2 :: Rosa26‐YFP :: Pdgfb‐hAPPSwInd) mice received tamoxifen to initiate the cre‐mediated YFP‐labelling of OPCs and their progeny. Tissue was analysed histologically 120 days later (P60 + 120) at P180. (a–b) Low magnification confocal image showing the hippocampus and fimbria in coronal brain cryosections from P60 + 120 control and APP transgenic mice stained to detect YFP (green), OLIG2 (red) and Hoechst [file JNR-98-1905-s001.pdf]

Supplementary Data

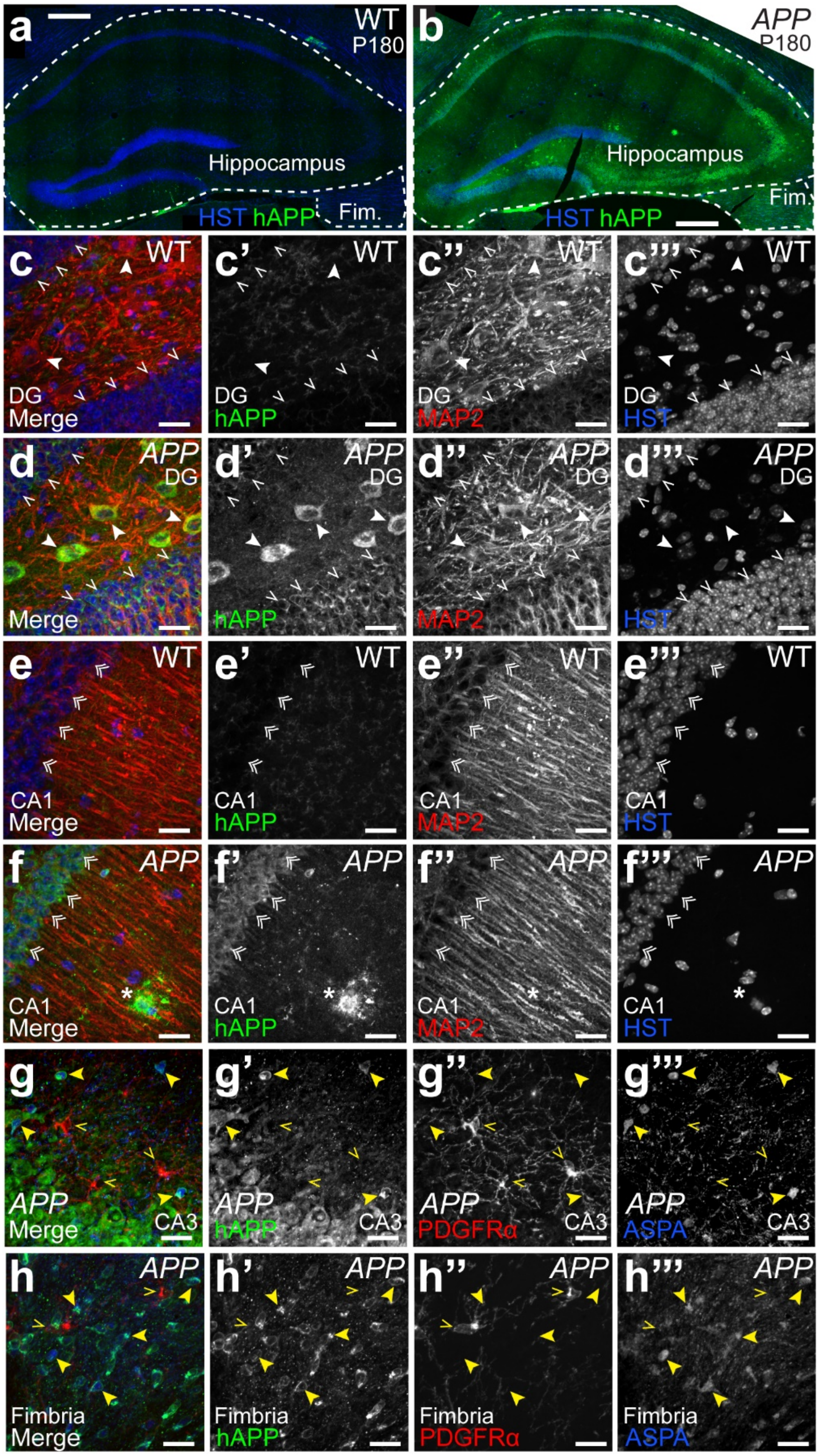

Legend on next page.

***Figure S1**: Human APP is expressed in hippocampal neurons and oligodendrocytes in APP mice*

a-b) Low magnification confocal images showing the hippocampus and fimbria of P180 WT and *APP* mice following immunohistochemistry to detect human APP (hAPP; green) and Hoechst 33342 (HST; blue). **c-d**) Confocal images show cells in the dentate gyrus (DG) of the hippocampus in P180 WT and *APP* mice that express hAPP (green), the microtubule associated protein 2 (MAP2, red; stains neuronal dendrites and cell bodies) and / or Hoechst 33342 (HST; blue). hAPP was not detected in any cells within the DG of WT mice (c) but was highly expressed by dentate granule neurons (open arrowheads) and mossy cells (solid arrowheads) in the DG of *APP* mice (d). **e-f**) Confocal images show cells in the CA1 region of the hippocampus in P180 WT and *APP* mice that express hAPP (green), MAP2 (red) and / or HST (blue). hAPP was not detected in cells within the CA1 of WT mice (e) but was highly expressed by pyramidal neurons (double arrowheads) in *APP* mice. **g-h**) Confocal images of cells within the CA3 region of the hippocampus (g) or fimbria (h) in P180 *APP* mice that are labelled with hAPP (green), PDGFRα (red) and / or ASPA (blue). hAPP was expressed by CA3 pyramidal neurons (g) and all $ASPA^+$ oligodendrocytes (solid yellow arrowheads), but no OPCs (open yellow arrowheads) in the hippocampus. * amyloid plaque. Scale bars represent 200 µm (a-b) or 20 µm (c-h).

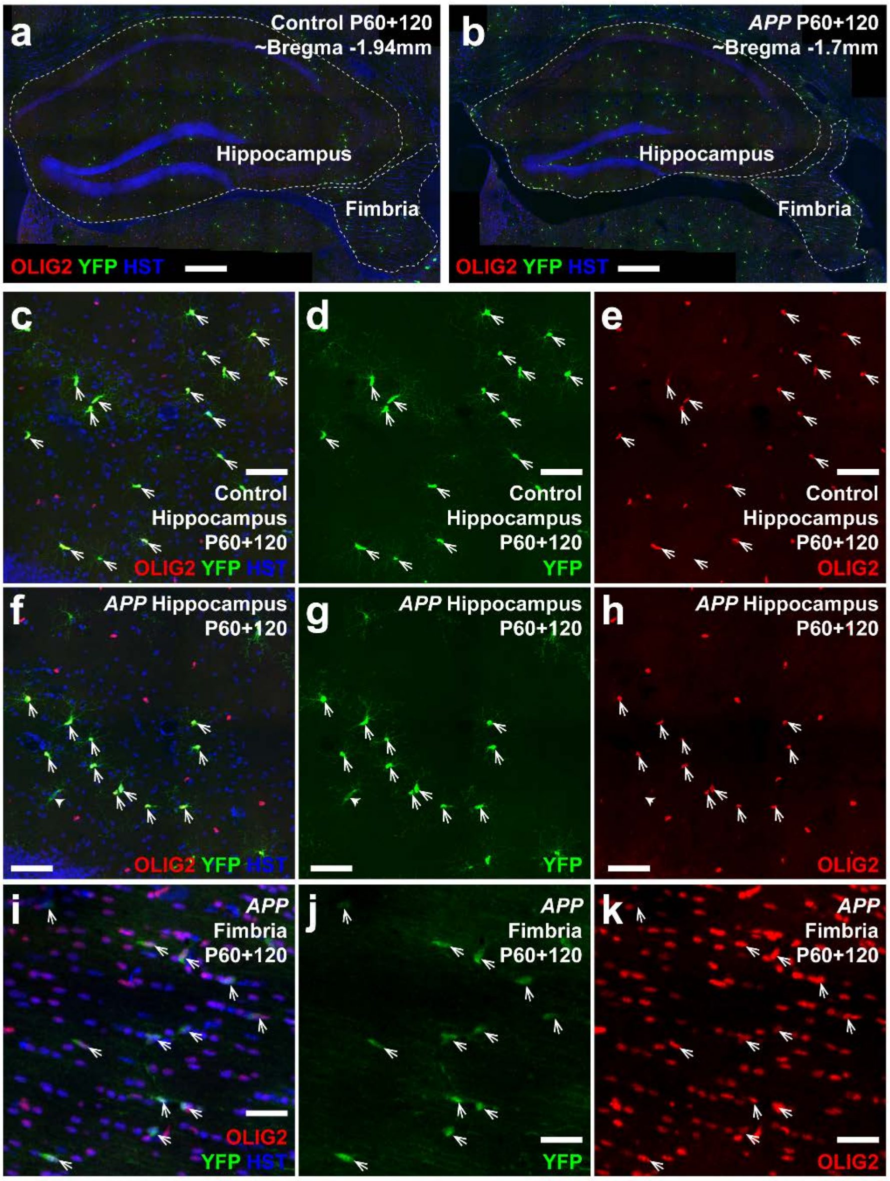

***Figure S2**: Essentially all YFP-labelled cells are OLIG2^{+} in P60+120 control and APP mice*

P60 control (*Pdgfrα-CreER^{T2} :: Rosa26-YFP*) and *APP* (*Pdgfrα-CreER^{T2} :: Rosa26-YFP :: Pdgfb-hAPP^{SwInd}*) mice received tamoxifen to initiate the cre-mediated YFP-labelling of OPCs and their progeny. Tissue was analysed histologically 120 days later (P60+120) at P180. **a-b**) Low magnification confocal image showing the hippocampus and fimbria in coronal brain cryosections from P60+120 control and *APP* transgenic mice stained to detect YFP (green), OLIG2 (red) and Hoechst 33342 (HST, blue). **c-e**) Confocal image showing cells in the hippocampus of P60+120 control mice that express YFP (green), OLIG2 (red) and HST (blue). **f-h**) Confocal image showing cells in the hippocampus of P60+120 *APP* transgenic mice that express YFP (green), OLIG2 (red) and HST (blue). **i-k**) Confocal image showing cells in the fimbria of P60+120 *APP* mice that express YFP (green), OLIG2 (red) and HST (blue). White arrows indicate YFP^{+} OLIG2^{+} cells. White arrow head shows an example of a rare YFP^{+} OLIG2-negative cell. Scale bar represents 280 µm (**a-b**), 50 µm (**c-h**), or 25 µm (**i-k**).

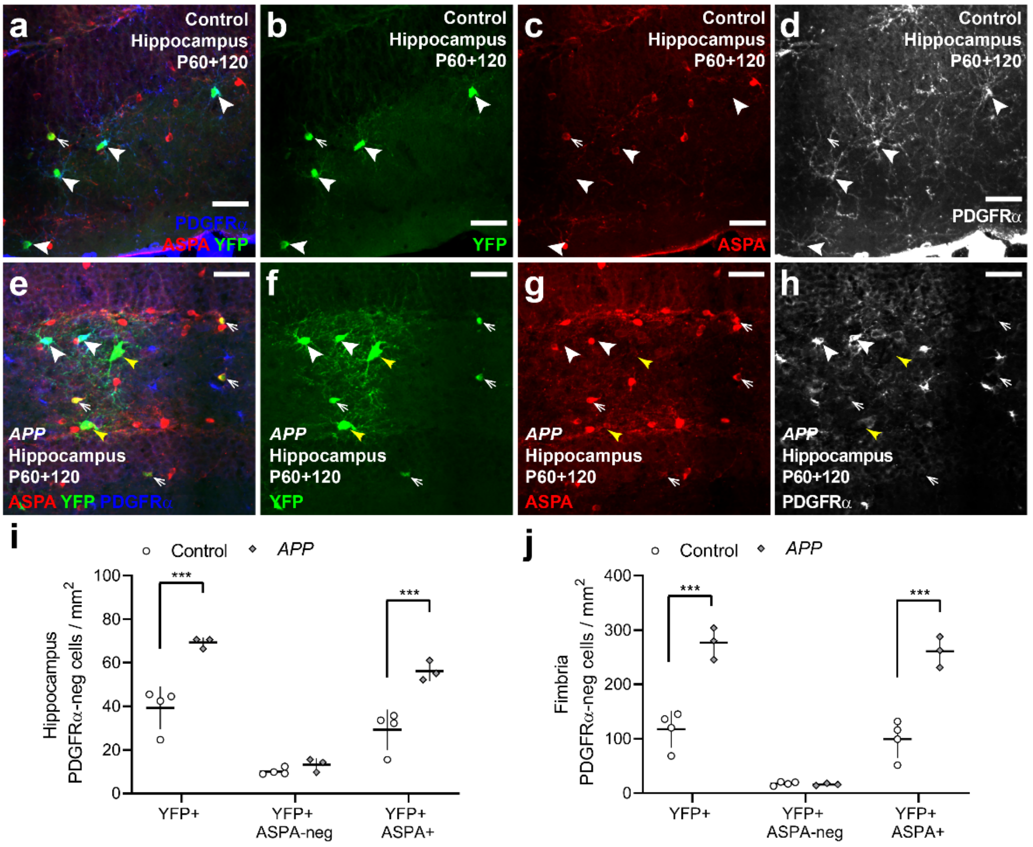

***Figure S3**: The density of mature (ASPA^{+}) newborn oligodendrocytes is increased in the hippocampus and fimbria of P60+120 APP mice*

P60 control (*Pdgfrα-CreER^{T2} :: Rosa26-YFP*) and *APP* (*Pdgfrα-CreER^{T2} :: Rosa26-YFP :: Pdgfb-hAPP^{SwInd}*) mice received tamoxifen to initiate the cre-mediated YFP-labelling of OPCs and their progeny. Tissue was analysed histologically 120 days later (P60+120) at P180. **a-d)** Confocal image of cells in the hippocampus of a P60+120 control mouse that express YFP (green), ASPA (red) and / or PDGFRα (blue). Panels show the fluorescent overlay and each channel separately. **e-h)** Confocal image of cells in the hippocampus of a P60+120 *APP* mouse that express YFP (green), ASPA (red) and / or PDGFRα (blue). Panels show the fluorescent overlay and each channel separately. **i)** Quantification of the density of: (i) newborn oligodendrocytes (YFP^{+} PDGFRα-neg), newborn immature oligodendrocytes (PDGFRα-neg, ASPA-neg) or newborn mature oligodendrocytes (PDGFRα-neg ASPA^{+}) in the hippocampus of P60+120 control or *APP* mice [Two-way ANOVA, genotype: $F(1, 15) = 49.33$, $p < 0.001$; cell type: $F(2, 15) = 80.29$, $p < 0.001$; interaction: $F(2, 15) = 8.83$, $p < 0.003$; n = 4 control and n = 3 *APP* mice]. **j)** Quantification of the density of: (i) newborn oligodendrocytes (YFP^{+} PDGFRα-neg), newborn immature oligodendrocytes (PDGFRα-neg, ASPA-neg) or newborn mature oligodendrocytes (PDGFRα-neg ASPA^{+}) in the fimbria of P60+120 control or *APP* mice [Two-way ANOVA, genotype: $F(1, 15) = 81.97$, $p < 0.001$; cell type: $F(2, 15) = 96.15$, $p < 0.001$; interaction: $F(2, 15) = 21.21$, $p < 0.001$; n = 4 control and n = 3 *APP* mice). *** $p < 0.001$ denotes significance from Bonferroni post-hoc analyses. White arrow heads indicate PDGFRα^{+} OPCs that are YFP-labelled; yellow arrow head indicate PDGFRα-neg YFP^{+} ASPA-neg presumptive immature (premyelinating) oligodendrocytes; white arrows indicate YFP^{+} ASPA^{+} newborn mature oligodendrocytes. Scale bars represent 35µm.

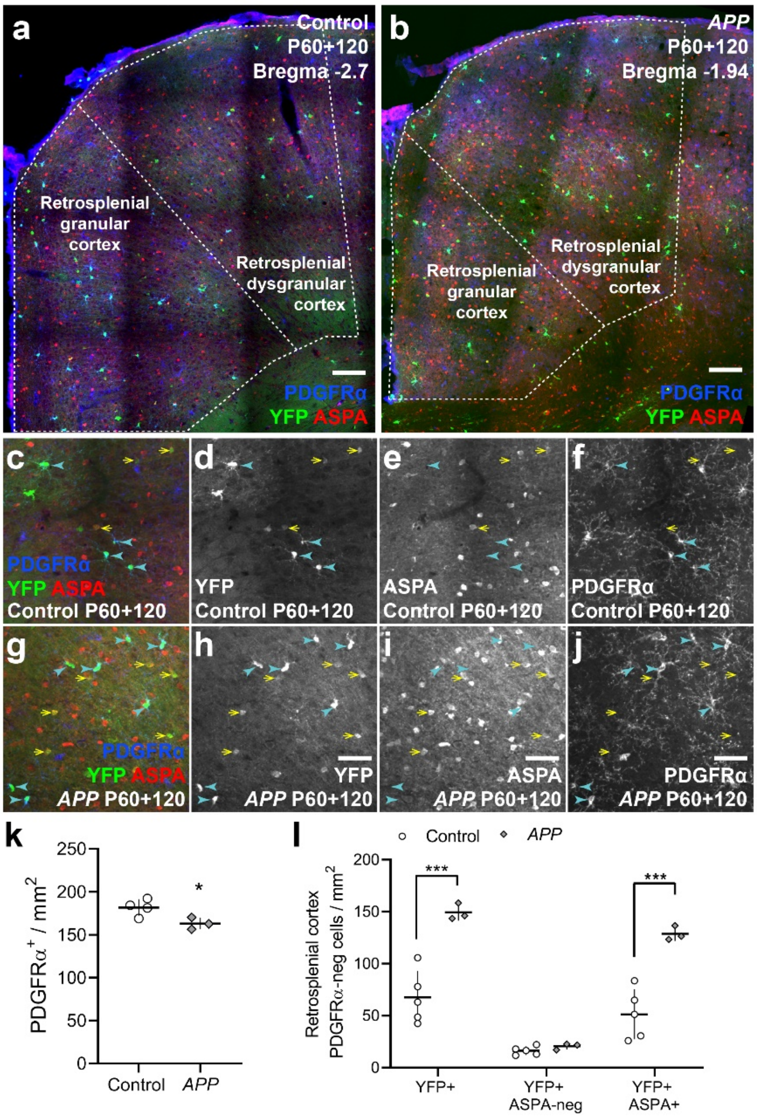

***Figure S4**: Oligodendrogenesis is increased in the retrosplenial cortex of P60+120 APP mice*

P60 control (*Pdgfrα-CreER^{T2} :: Rosa26-YFP*) and *APP* (*Pdgfrα-CreER^{T2} :: Rosa26-YFP :: Pdgfb-hAPP^{SwInd}*) mice received tamoxifen to initiate the cre-mediated YFP-labelling of OPCs and their progeny. Tissue was analysed histologically 120 days later (P60+120) at P180. **a-b**) Low magnification confocal images show the retrosplenial granular cortex and the retrosplenial dysgranular cortex (counted together as the retrosplenial cortex) in coronal cryosections from P60+120 control or *APP* mice following immunohistochemistry to detect YFP (green), ASPA (red) and PDGFRα (blue). **c-f)** Confocal images of cells in the retrosplenial cortex of a P60+120 control mouse that express YFP (green), ASPA (red) and / or PDGFRα (blue). Panels show the fluorescent overlay and each channel separately. **g-j)** Confocal image of cells in the retrosplenial cortex of a P60+120 *APP* mouse that express YFP (green), ASPA (red) and / or PDGFRα (blue). Panels show the fluorescent overlay and each channel separately. **k)** Quantification of the density of PDGFRα$^+$ OPCs in the retrosplenial cortex of P60+120 control or *APP* mice [Two-tailed, unpaired t-test, t (5) = 2.80; n = 4 control and n = 3 *APP* mice]. **l**) Quantification of the density of: (i) newborn oligodendrocytes (YFP$^+$ PDGFRα-neg), newborn immature oligodendrocytes (PDGFRα-neg, ASPA-neg) or newborn mature oligodendrocytes

(PDGFRα-neg ASPA$^+$) in the retrosplenial cortex of P60+120 control or *APP* mice [Two-way ANOVA, genotype: $F(1, 18) = 58.04$, $p < 0.001$; cell type: $F(2, 18) = 58.84$, $p < 0.001$; interaction: $F(2, 18) = 12.37$, $p < 0.001$; n = 4 control and n= 3 *APP* mice]. * $p < 0.05$ and *** $p < 0.001$ denote the significance of the unpaired t-test or Bonferroni post-hoc test. Blue arrows indicate PDGFRα$^+$ OPCs that are YFP-labelled; yellow arrows indicate YFP$^+$ ASPA$^+$ newborn mature oligodendrocytes. Scale bars represent 120μm (a-b) or 35μm (c-j).

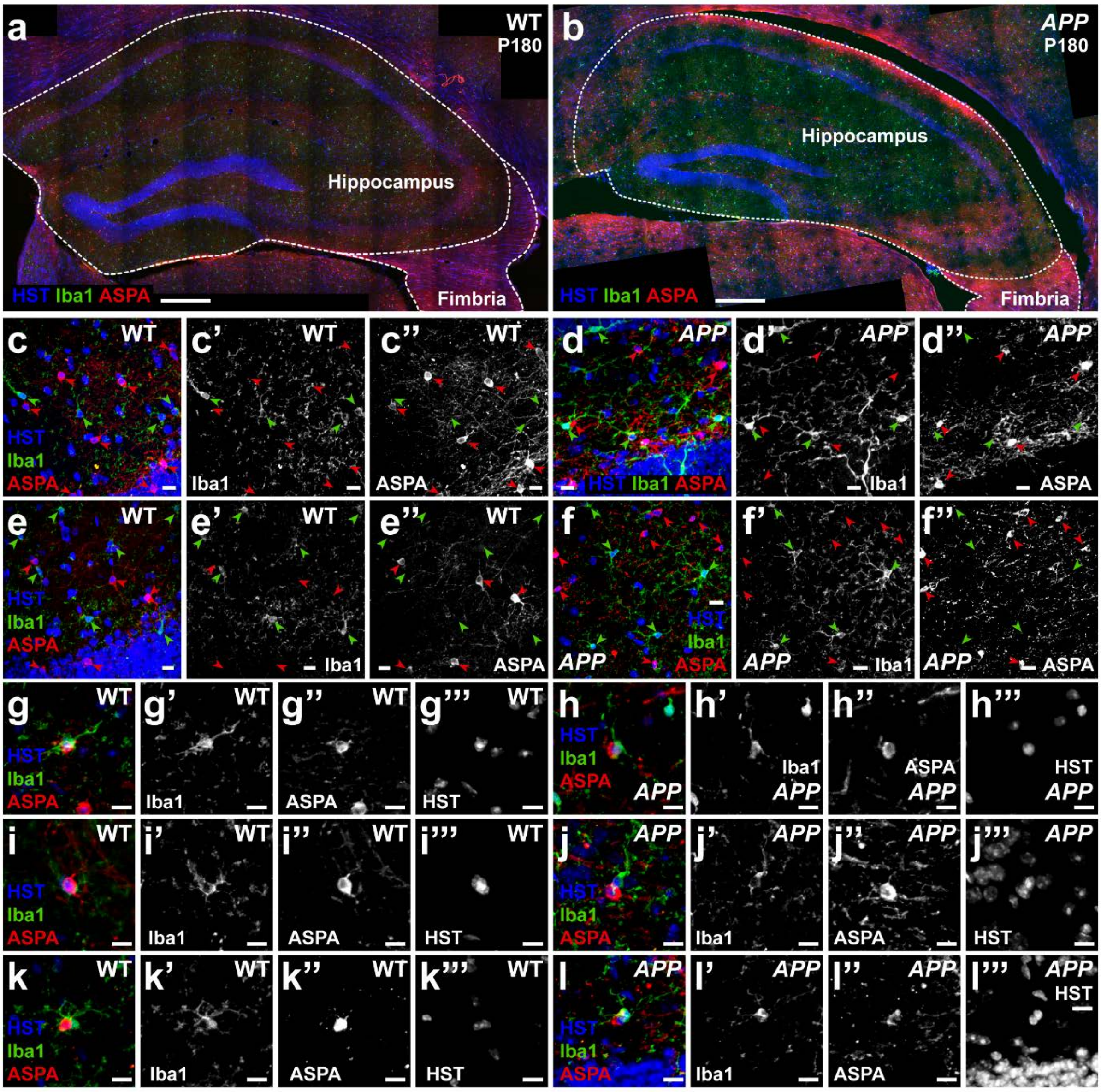

***Figure S5**: APSA⁺ cells in the hippocampus of P180 WT or APP mice do not express the microglial marker Iba1*

a-b) Low magnification confocal images showing the hippocampus and fimbria in coronal brain cryosections (30µm) from P180 WT and *APP* mice following immunohistochemistry to detect the microglial marker Iba1 (green), the mature oligodendrocyte marker ASPA (red) and the nuclear label Hoechst 33342 (HST, blue). We determined that 0 of 1672 ASPA$^+$ cells analysed in WT mice (n=4) and 0 of the 993 ASPA$^+$ cells analysed in APP mice (n=3) co-labelled for Iba1. **c-f**) Confocal images showing cells within the hippocampus of WT and *APP* mice that label for Iba1 or ASPA, demonstrating that individual cells do not co-express these markers. Green arrows denote Iba1$^+$ microglia and red arrows denote ASPA$^+$ mature oligodendrocytes. **g-l**) Confocal images showing examples of closely apposed Iba1$^+$ microglia and ASPA$^+$ mature oligodendrocytes (2 closely overlapping nuclei and cells of distinct shapes) within the hippocampus of WT and *APP* mice (accounts for ~2% of all APSA$^+$ cells). Scale bars represent 280 µm (**a-b**) or 10 µm (**c-1**).

Table S1: List of antibodies used in this research.

**Antibody**	**Immunogen**	**Manufacturer**<br>**Catalogue #**<br>**RRID**<br>**Species**<br>**Clonality**	**Conc**	**Characterisation information.**<br>* = see comment below
6E10<br>(anti-Aβ)	AA residues 1-16 of β -amyloid, epitope within AA 3-8 (EFRHDS) of β-amyloid	Covance<br>Cat #: SIG-39320<br>RRID: AB_ 662798<br>Mouse<br>Monoclonal	1:500	*(Collins et al., 2015)
β -actin	Modified β-cytoplasmic actin N-terminal peptide, Ac-Asp-Asp-Asp-Ile-Ala-Ala-Leu-Val-Ile-Asp-Asn-Gly-Ser-Gly-Lys, conjugated to KLH	Sigma-Aldrich<br>Cat #: A1978<br>RRID: AB_476692<br>Mouse<br>Monoclonal	1:5000	* Used for protein quantification in Western blot analysis as per (Ferreira et al., 2020)
Goat anti-mouse Immunoglobulins - HRP	Immunoglobulins, mainly IgG, isolated from mouse serum	Dako (Agilent)<br>Cat #: P0447<br>RRID: AB_2617137<br>Goat<br>Polyclonal	1:10000	* Used for protein quantification in Western blot analysis as per (Ferreira et al., 2020)
PDGFRα<br>(Platelet derived growth factor receptor α)	Mouse myeloma cell line NS0-derived recombinant mouse PDGFRα Leu25-Glu524 (Asp65Glu, Gly439Ala, Thr440Ala)	R&D Systems<br>Cat #: AF1062<br>RRID: AB_2236897<br>Goat<br>Polyclonal	1:200	* (O’Rourke et al., 2016)
Iba1<br>(Ionized calcium binding adaptor molecule 1)	Synthetic peptide corresponding to AA 134 to 147 from rat IBA1	Synaptic Systems<br>Cat #: 234 004<br>RRID: AB_2493179<br>Guinea pig<br>Polyclonal	1:500	* (Auderset et al., 2016; Ferreira et al., 2020)

ASPA<br><br>(Aspartoacylase)	Full-length recombinant mouse ASPA/Nur7	Merck Millipore<br><br>Cat #: ABN1698<br><br>RRID: AB_2827931<br><br>Rabbit<br><br>Polyclonal	1:200	* (Madhavarao et al., 2004 (rat); Ferreira et al., 2020 (mouse)). Lack of reactivity to Iba1$^+$ microglia demonstrated in present study.
GFP<br><br>(Green fluorescent protein)	His-GFP (full-length) fusion protein	Nacalai Tesque<br><br>Cat #: 04404-84<br><br>RRID: AB_10013361<br><br>Rat<br><br>Monoclonal	1:2000	* (Ferreira et al., 2020; O'Rourke et al., 2016) Does not stain GFP negative tissue.
Nav1.6<br><br>(Voltage gated sodium channel 1.6)	Peptide CIANHTGVDIHRN GDFQKNG, corresponding to AA residues 1042-1061 of rat NaV1.6	Alomone labs<br><br>Cat #: ASC-009<br><br>RRID: AB_2040202<br><br>Rabbit<br><br>Polyclonal	1:200	*(Young et al., 2013)
OLIG2<br><br>(Oligodendrocyte Transcription Factor 2)	Recombinant mouse Olig-2 (epitope region N/A)	Merck Millipore<br><br>Cat #: AB9610<br><br>RRID: AB_570666<br><br>Rabbit<br><br>Polyclonal	1:400	* (Ferreira et al., 2020; Young et al., 2013)
Caspr<br><br>(Contactin-associated protein)	Clone K65/35 Recombinant protein corresponding to rat Caspr (epitope region unknown)	Neuromab<br><br>Cat #: MABN69<br><br>RRID: AB_10806491<br><br>Mouse<br><br>Monoclonal	1:200	* (Young et al., 2013)
MAP2<br><br>(Microtubule-associated protein 2)	Purified Microtubule-associated protein from rat brain (epitope region N/A)	Merck Millipore<br><br>Cat #: AB5622<br><br>RRID: AB_91939<br><br>Rabbit<br><br>Polyclonal	1:1000	* (Carasatorre et al., 2015)
* Denotes antibody is commercially available and was used for Western blot visualisation and quantification or as a tissue marker which stained the appropriate pattern of cellular morphology and distribution as demonstrated in previous publications (example references given).				

**Secondary Antibodies:**		**All Life Technologies (Thermo Fisher Scientific)**		
Donkey anti-Rat IgG (H+L) - Alexa Fluor 488	IgG Heavy and Light chains	Cat #: A21208<br>RRID: AB_2535794<br>Donkey<br>Polyclonal	1:500	
Goat anti-Guinea pig IgG (H+L) - Alexa Fluor 488	IgG Heavy and Light chains	Cat #: A-11073<br>RRID: AB_2534117<br>Goat<br>Polyclonal	1:1000	
Donkey anti-Rabbit IgG (H+L) - Alexa Fluor 488	IgG Heavy and Light chains	Cat #: A21206<br>RRID: AB_2535792<br>Donkey<br>Polyclonal	1:1000	
Donkey anti-Rabbit IgG (H+L) - Alexa Fluor 568	IgG Heavy and Light chains	Cat #: A10042<br>RRID: AB_2534017<br>Donkey<br>Polyclonal	1: 1000	
Donkey anti-Rabbit IgG (H+L) - Alexa Fluor 647	IgG Heavy and Light chains	Cat #: A-31573<br>RRID: AB_2536183<br>Donkey<br>Polyclonal	1:1000	
Donkey anti-Goat IgG (H+L) - Alexa Fluor 488	IgG Heavy and Light chains	Cat #: A11055<br>RRID: AB_2534102<br>Donkey<br>Polyclonal	1:1000	
Donkey anti-Goat IgG (H+L) - Alexa Fluor 568	IgG Heavy and Light chains	Cat #: A-11057<br>RRID: AB_2534104<br>Donkey<br>Polyclonal	1:1000	

Donkey anti-Goat IgG (H+L) - Alexa Fluor 647	IgG Heavy and Light chains	Cat #: A21447<br><br>RRID: AB_2535864<br><br>Donkey<br><br>Polyclonal	1:1000	
Donkey anti-Mouse IgG (H+L) - Alexa Fluor 647	IgG Heavy and Light chains	Cat #: A31571<br><br>RRID: AB_162542<br><br>Donkey<br><br>Polyclonal	1:1000	
**Other imaging markers:**				
Hoechst 33342	N/A (dsDNA)	Invitrogen<br><br>(Thermo Fisher Scientific)<br><br>Cat #: H21492	1: 10000	
Thioflavine S	N/A (β-amyloid structures)	Sigma-Aldrich<br><br>Cat #: T1892	0.1% (w/v)	
