# Supplementary material for: Amyloidosis is associated with thicker myelin and increased oligodendrogenesis in the adult mouse brain
Source: J Neurosci Res. 2020 Jun 18;98(10):1905–32. doi: 10.1002/jnr.24672 (PMC7540704; doi:10.1002/jnr.24672)
Supplement: Supplementary file 3 — Transparent Peer Review Report [file JNR-98-1905-s003.docx]

Amyloidosis is associated with the formation of thicker myelin in development and increased adult oligodendrogenesis

Solène Ferreira, Kimberley A Pitman, Shiwei Wang, Benjamin S Summers, Nicole Bye, Kaylene M Young and Carlie L Cullen

| Review timeline: | Submission date: 23-Jan-2020  Editorial Decision: Major Revision 09-Mar-2020  Revision Received: 03-May-2020  Accepted: 25-May-2020 |  |
| --- | --- | --- |
|  |  |  |

Editor: Christopher Colwell

Statistical reviewer: David McArthur

Reviewer 1: Anthony Don

Reviewer 2: Cecile Delarasse

Reviewer 3: Dan Fulton

_____________________________________________________________________________________

1^st^ Editorial Decision

09-Mar-2020

_____________________________________________________________________________________

Dear Dr Cullen:

Thank you for submitting your manuscript to the Journal of Neuroscience Research. We've now received the reviewer feedback and have appended those reviews below. As you will see, the reviewers find the question addressed to be of potential interest. Yet, they do not find the manuscript suitable for publication in its current form.

If you feel that you can adequately address the concerns of the reviewers, you may revise and resubmit your paper within 60 days. It will require further review. Please explain in your cover letter how you have changed the present version. If you require longer than 60 days to make the revisions, please contact Dr Cristina Ghiani (cghiani@mednet.ucla.edu). You can submit your revised manuscript directly by clicking on the following link: *** PLEASE NOTE: This is a two-step process. After clicking on the link, you will be directed to a webpage to confirm. ***

https://mc.manuscriptcentral.com/jnr?URL_MASK=541c32a434674e75a68a29403ed55bbd

Thank you again for your submission to the Journal of Neuroscience Research; we look forward to reading your revised manuscript.

Best Wishes,

Dr Christopher Colwell
Associate Editor, Journal of Neuroscience Research

Dr Cristina Ghiani
Co-Editor-in-Chief, Journal of Neuroscience Research


Associate Editor: Colwell, Christopher
Comments to the Author:
Your manuscript has now been reviewed by three experts in this field as well as by our statistical editor.  
The reviewers all agree that this study has much merit. They also raised significant concerns that would require major revisions.  

The statistic editor makes a good point for the use of RM ANOVA for your analysis.  
A major concern is the low number of animals used in some of the assays. Please address this point as also suggested by the statistic editor.
This is not a proper form to report the number of animals "n ≥ 3 mice per genotype", please add the number of animals/group in the figure legends/each experiment.

Total numbers of animals used is not included.

Figure 3, The low magnification image (panels A and B) should include the fimbria.
Figure 5, 7 and 8, low magnification images (similar to supplementary figure 1) of the slices used to acquire the higher magnifications should be included. The areas of the high magnification images should be indicated in these.

We will look forward to seeing a revised version of the ms.    

Additional Editorial Comments:

The authors should state whether animals from both sexes were used.

SEX DIFFERENCES
The National Institutes of Health now mandates the inclusion of sex as a biological variable. To conform with this mandate, the Journal of Neuroscience Research has established new policy (please see our editorial: http://onlinelibrary.wiley.com/doi/10.1002/jnr.23979/full) requiring all authors to ensure proper consideration of sex as a biological variable. Please ensure that: 1) Any paper utilizing subjects of one sex state the sex of the sample in the title and abstract; 2) The number of samples/subjects of each sex used in the research must be clearly stated in the methods section; 3) The inability for any reason to study sex differences where they may exist should be discussed as a study limitation. We are also encouraging authors to report exploratory analyses of potential sex differences in studies not explicitly designed to address them

Please move the Acknowledgements and authors contribution before the references. The latter must follow the CRediT Taxonomy. You can find examples of such statements in the author guidelines on-line at http://onlinelibrary.wiley.com/journal/10.1002/(ISSN)1097-4547/homepage/ForAuthors.html

CONFLICT OF INTEREST
Please add to your paper (after the Discussion and Acknowledgments, immediately before the References) a conflict of interest statement

GRAPHICAL ABSTRACT
Please upload a graphical abstract, which we are asking of all authors submitting original research articles. This is intended to provide readers with a visual representation of the conclusions and an additional way to access the contents and appreciate the main message of the work. What we require is a .tif image file and a .doc text file containing an abbreviated abstract. For the image, labels, although useful, must be kept to a minimum and the image should be 400 x 300, 300 x 400, or 400 x 400 pixels square and at a resolution of 72 dpi. This can be one of the figures from your article, or something slightly different, as long as it represents your study.  Instructions for this can be found in our author guidelines online at http://onlinelibrary.wiley.com/journal/10.1002/(ISSN)1097-4547/homepage/ForAuthors.html

RESOURCE IDENTIFICATION INITIATIVE (Abbreviated Text)
Please incorporate Research Resource Identifiers (RRIDs) in your citation of all resources used in your manuscript (antibodies, software tools, databases, model organisms) where applicable in the text, exactly as you would a regular citation or Genbank Accession number.  Please also be sure these RRIDs are included in your keywords list in addition to the required keywords. For any antibodies, we ask that you also include the RRIDs in your antibody table, in addition to citing them in the text. An example of how to list RRIDs in your antibody table can found in the example antibody table attached and for more information about how to obtain and cite RRIDs within your text, please visit the "Resource Identification Initiative" section of our author guidelines: http://onlinelibrary.wiley.com/journal/10.1002/(ISSN)1097-4547/homepage/ForAuthors.html

Furthermore, in accord with JNR requirements, you will have to provide additional information on each of the antibodies that you use.  This must include the source (not just the manufacturer and catalog or lot number, but the species it was raised in, and the EXACT structure of the immunizing antigen - including the amino acid sequence for peptide antigens); characterization (e.g., what does it recognize on immunoblots); and appropriate controls (i.e. the effect of blocking peptides for immunohistotochemical localization).  


Statistics Editor: McArthur, David
Comments to the Author:
Use of one-way or two-way anova for those measures like the memory tasks gathered repeatedly across time on the same individuals (as is to be inferred from the narrative but never explicitly stated) is incorrect.  If indeed one or more is a  repeated measures design, they have traditionally been analyzed with repeated-measures anova that method has now been superseded for multiple reasons by mixed effects modeling, with appropriate post-hoc contrasts.  Prism 8 is able to analyze repeated measures data by fitting a mixed effects model.  Kindly consult a trained biostatistician to explain the details of that analytic approach to you.  

For those studies with n=3 (or quizzically in some instances "n>3"), please be aware that with quite small sample sizes, whatever findings are reported must be interpreted with great caution.  Kindly discuss this limitation in the narrative.  Assumption of a normal distribution for those data is unwarranted.  There is no evidence of power analysis.  On the mandated Questionnaire (not provided) item 3a asks that a sample size rationale be provided,

Why are some of your data denoted with SDs but others with SEMs?  The use of SEM is not provided with a rationale.  SD's are preferred.  SEMS's calculated on separate data distributions that all have identical means and identical variances will be the same only if the number of cases in all groups is also the same.  Thus SEMs cannot be interpreted as a stable reflection of variability for a given set of data distributions, and the visualizations using SEMs are thus also not stable.  It is for these reasons that 21st century statistical thinking has pushed for SDs.  

This journal requests that p-values be shown using a consistent decimal exactness: numerous values in this text are given to varying number of digits. Kindly choose a decimal, say 3, and stay with it throughout, with "p<0.001" reserved as appropriate.

This journal does not accept "dynamite plots" (also called "plunger plots", "toiletbrush plots", or "bogbrush plots") as they are often severe distortions of the actual data distributions.  When samples are small, kindly use dotplots, else use boxplots or violin plots with or without dotplot overlays.  You've already included the dots; simply delete the bars altogether as they add nothing of value.

The captions for Figures 2 shows "as the target zone. p)" but that "p" may be a typo.

Figure 6d is unacceptable if any of the symbols are completely overwritten by other symbols.  In this instance you'll want to explore density plots, for which a variety of options are available.

Reviewer: 1

Comments to the Author
This paper presents well researched and novel findings on the effect of expression of an amyloidogenic transgene (APP-SwInd) on the number, proliferation, and electrophysiological properties of oligodendrocyte progenitors. The research is quite descriptive, without any deep or well-connected mechanistic insight into how the amyloid transgene produces these effects on OPCs and oligodendrocytes. However, it is a comprehensive body of work and suitable for publication in a good journal.

The paper is well written. However the discussion (although well written) is too long. I estimated almost 3600 words.
(1) The authors really should cut this back to something more like 2000 words as it gets difficult to read by half way through.

(2) Can the authors present evidence indicating whether or not APP is expressed exclusively in neurons in this model, or also in glial cells (oligodendrocytes)? This should be feasible using the antibody to human APP and is quite important in terms of establishing that the effects on oligodendrocytes are caused by APP expression and/or amyloid beta production in neurons.

On p.120 the authors propose that oligodendrocyte turnover is higher in APP transgenic mice by P180. Why not P120, as figure 7 clearly shows higher OPC conversion to oligodendrocytes at P120 in the APP transgenic mice?
(3) Can the authors quantify the proportion of mature oligodendrocytes (ASPA positive) that are YFP-positive vs YFP-negative at P120? Would this indicate whether there is higher oligodendrocyte turnover in the APP transgenic mice?

Lastly, I note that in this paper, like every other paper using APP overexpression mouse models, it is assumed that the effects seen are attributed to the mutant form of APP and, by extension, amyloid beta, rather than APP overexpression itself. It is generally believed that soluble amyloid beta levels increase prior to amyloid beta plaque formation.
(4) Do the authors have any indication or measure for amyloid beta production prior to P180? Are the behavioural changes driven by amyloid generation or APP overexpression?

Minor comments:
(5) The authors should read through their methods section again and add spacing between the numeral and the unit of measure where this has not been done, e.g. should be 2 mm, not 2mm (p.6); 300 mg, not 300mg (p.5).
(6) Show the position of molecular weight markers on Figure 1a.
(7) Correct the sentence on p.10, “formation of amyloid plaque formation”.
(8) First sentence of second paragraph on p. 11 needs correcting “…imbalance in the brain, we next used whole cell…”
(9) Explain more clearly in the methods how the % error in the T-maze is calculated. Is this the % for same arm entries/total arm entries?


Reviewer: 2

Comments to the Author
In this study, the authors investigated whether amyloid pathology might affect myelin and oligodendrocytes properties in an APP mouse transgenic model (APP mice). First, they characterized the animal model and observed in the hippocampus of the mice expression of human APP that increased with aged and amyloid plaque formation at P180 as previously described. The APP mice does not develop spatial learning deficit at P180 but exhibit an anxiety-like behaviour. To determine the impairment of oligodendrocyte progenitors (OPCs), they performed electrophysiological experiment and show that the membrane properties of OPCS are unchanged but the response of OPC to GABA is increased at P100 in the hippocampus of APP mice. In addition, their data indicate that the Node of Ranvier is decreased and paranode length is increased in APP mice. By transmission electron microscopy, the authors show that the number of myelinated axons and the diameter were equivalent between WT and APP mice while the myelin thickness is increased in the hippocampus of P100 APP mice. Using an elegant strategy of cre-lox lineage tracing of adult OPCs in APP mice, the authors suggest that amyloid pathology is associated with an increase turnover of oligodendrocytes. The experiments are very well performed. The manuscript is clear and well written. This is a very interesting study showing myelin abnormalities in amyloid mouse model. The discussion of the manuscript could be improved to highlight the relevance of the data with Alzheimer’s disease.

The authors should indicate in the manuscript that presenilin not only mediates the cleavage of APP but also of Notch receptors to enable signalling by their cytoplasmic domains. It is important to mention this because Notch signalling pathway controls oligodendrocyte maturation and myelination of axons. This would highlight the pertinence of studying a model without PSEN1 mutation.

Could the authors determine in OPCs whether the expression level of GABAA receptor subunits or other related channels are modify in APP mice that may explain the increased response to GABA.

The authors should evaluate the potential effects on neurons of myelin thickness increased and modification of node of Ranvier. Are the number of neurons, the synaptic density modified ?
The discussion on the expression of the human APP transgene is too long in relation to the main topic of the study. Also, the discussion related to behavioral study is off topic. It is difficult to compare behavioral experiments that use different paradigms and mouse strains. The authors should rather discuss their behavioral results in the context of myelin and oligodendrocyte impairments they observed; and how these impairments may explain or not the anxiety and memory phenotypes. What are the potential physiological effects of decreased Node of Ranvier and increased paranode length in APP mice ?


I do not understand the hypothesis that “the increased myelin thickness could result from an upregulation in neuregulin-1 type III expression”. Could the authors clarify this point since there is no mutation of BACE1 in this transgenic model ?


Reviewer: 3

Comments to the Author
Summary
The authors have used a transgenic approach to  study the effect of amyloidosis on the development and physiology of oligodendrocytes (OL) in mouse hippocampus. Specifically, they aimed to determine if mutations in a single AD related gene, APP, could drive changes in OL function during the early stages of disease since previous knowledge in this area has been derived from models involving multiple gene mutations. Mice over expressing a mutant form of the amyloid precursor protein (APP) containing two mutations associated with human familial Alzheimer’s Disease (AD) were used. APP over expressing mice exhibited populations of hippocampal OPC whose density  remained largely unchanged through early and mid-adult ages. However, a Cre/Lox fate mapping study showed show that by mid-adulthood hippocampal OPC exhibited an increased capacity for differentiation within the hippocampus and neighbouring tissues. Interestingly, immunostaining for OL failed to detect a change in the overall density of OL leading the authors to propose a link between amyloid pathology and an increase in OL turnover. The authors also performed a comprehensive analysis of hippocampal OPC physiology using patch-clamp recordings targeted to GFP labeled OPC. Although these recordings revealed unchanged membrane properties and responses to Kainate, an increase in sensitivity to GABA was detected by P100 in APP mice. The authors discuss this interesting observation in the light of recent findings on the influence of GABA on cortical myelination and cerebellar OPC. Immunostaining and EM approaches were also used to examine changes in myelinated axons. Here, the authors report that by P90-100 APP mice display a reduction in nodal length, increased paranodal length, and thicker myelin. Behavioural tests, including the spontaneous alternating T maze and Barnes maze, were also carried out to assess performance in hippocampal-dependent tasks. Testing in the T maze task revealed deficits in working memory by P90, while their performance in the Barnes maze, which probes long-term spatial memory, remained unchanged at P180. The authors discuss this latter finding in the context of contrasting evidence from the field, and their own data showing clear evidence of amyloid plaques at this age within the hippocampi of P180 APP mice.

Overall the authors propose that their findings reveal changes in OPC and OL function that may contribute to the early stages of cognitive dysfunction in amyloid related disease.

Strengths
This work investigates an important question (contribution of OPC / OL to early stages of amyloid driven pathology) with a combination of appropriate state-of-the-art techniques. The experiments have been designed well and examine OL form and function at a number of important levels, with the resulting data being presented clearly, and discussed throughly in the context of knowledge in the field. The use of a second AD related transgenic line (MAPT) allowed the dissection of APP related changes, while the Cre/Lox fate mapping approach provided valuable information on adult oligodendrogenesis.

Main comments
1. anti-ASPA was used in Figure 8 to analyse the total population of OL. The abbreviation for ASPA was not defined in the Methods section. One possibility is that ASPA refers to aspartoacylase since this protein has been detected in the majority of differentiated OL (see URL links below).  According to the papers listed below aspartoacylase is also detected in microglia throughout the brain. A count of ASPA+ cells might therefore be expected to include both microglia and differentiated OL. In this case the conclusions relating to Figure 8 would not be valid - e.g. that there is no overall change in the density of mature OL, and that there is a high turnover of OL in the APP hippocampus, fimbria and EC. If ASPA denotes aspartoacylase then I would recommend the use of the CC1 marker (anti-APC) to label differentiated OL.  
   1. Moffett et al. 2011 Glia 59: 1414 PMID 21598311 https://www.ncbi.nlm.nih.gov/pmc/articles/PMC3143213/
   2. Madhavarao et al. 2004 J Comp Neurol PMID 15065127 https://www.ncbi.nlm.nih.gov/pubmed/15065127
2. Relating to point 1, if amyloid pathology involves increased turnover of OL it should be possible to detect an increase in OL cell death. This could be examined by histological analysis of apoptosis/necroptosis in APP and control sections at P180, or by examining chromatin condensation in existing EM micrographs.
3. Although the highest density of plaques in the J20 mice occur in the hippocampus, these mice also show amyloid depositions in the neocortex: https://www.ncbi.nlm.nih.gov/pubmed/30311654. Likewise, Alzheimer's involves clear pathology and dysfunction in the neocortex. The authors should explain more clearly in the introduction (last paragraph) the rationale for focusing solely on changes in the hippocampus. The horizontal brain sections they have used to collect data on OPC density, adult oligodendrogeneisis, nodal / paranodal morphology, amyloid deposition etc will also contain overlying neocortical areas of interest. Knowledge on whether APP mice exhibit similar changes to OPC and myelin in cortical grey matter regions associated with spatial memory, e.g. retrosplenial cortex, would be of great interest to the field.
4. Hippocampal OPC in P100 APP mice develop a greater sensitivity to bath applied GABA. As discussed by the authors, these findings are of interest since GABA is known to influence OPC function and myelination in the cerebellum and neocortex (as discussed already on pages 16/17) . Given this knowledge it would be useful to have a clearer view of the physiological relevance of the alterations in GABA sensitivity. Do the increased currents observed with bath applied GABA translate into measurable changes in the properties of GABAergic synaptic inputs? Although additional patch-clamping experiments would be beyond the scope of the present work, the authors should comment directly on the potential for changes induced through amyloid pathology to directly influence inhibitory interneuron-OPC signalling.
5. The authors show that there is an increase in adult oligodendrogenesis in the hippocampus, EC and Fimbria of APP mice. Do these newborn differentiated OL (e.g. PDGFRa negative / YFP+ cells) show signs of myelinating? Can this be deduced from YFP+ morphology? The YFP labeled OL in figure 7i seem to show a myelinating morphology. If additional sections are available, and YFP morphology is not reliable, the authors should consider using MBP or PLP staining. It would be interesting to know if these newly generated OL contribute to myelination.
6. Changes in cognitive function are detected by P90 in the T maze task (more repeat entries in the APP mice) while changes in OPC function and myelin structure appear soon after at P100. The timing of these events is interesting. Although the authors acknowledge that impairments in the T maze changes may stem from the hyperactivity characterised in these mice (page 14), working memory in the T maze task is also hippocampus-dependent, thus it would be helpful for the authors to comment on the potential link between working memory impairment and alterations in OPC and OL function.

Minor comments
Methods
1. Catalogue numbers should be included for the antibodies.
2. Why assume a normal distribution for N=3 experiments? (page 9)
3. Quantification of nodes / paranodes needs a little more description, e.g. how many fields / section, how many sections per animal?
4. Ki67 is mentioned in the list of antibodies, but has not been used, please clarify.
5. Cortical used twice in same sentence (page 17, 2nd paragraph)
6. Labelling of Y axis in Figure 7  p q and r is confusing, it mentions PDGRa- YFP+ cells, but legend describes the axis as YFP+ olig2+ PDGFRa -ve cells. Please clarify.

____________________________________________________________________________________

Author’s Response

03-May-2020

_____________________________________________________________________________________

**Reply to Reviewers**

We thank the reviewers for their constructive feedback and thank the editor for giving us 60 days to respond to these comments. In this reply we list the reviewer’s comments (italics) and detail the actions taken to address them. We are unable to access our laboratory to carry out further experiments at this time, however, we were able to carry out additional histological experiments just prior to the shut-down and have been able to revise the text to address other concerns. We trust that our altered manuscript will now be suitable for publication in *The Journal of Neuroscience Research*.

***Editor:***  *The statistic editor makes a good point for the use of RM ANOVA for your analysis.*

As the behavioural assessments can only be carried out once on any given mouse, we analysed distinct cohorts of mice at each time-point. As we did not repeatedly assess the same mice, a repeated measures ANOVA cannot be applied to analyse these data. We have now clarified this in the methods (page 6):

“Behavioural testing was carried out on wildtype (WT) and APP transgenic littermates at P60, P90 or P180. Individual mice were tested once, and separate cohorts analysed at each age.”

And in the results (page 11):

“To compare the cognitive performance of WT and APP transgenic mice prior to and during plaque formation, WT and APP mice were subjected to a battery of behavioural tasks at P60, P90 or P180, with each age representing a separate cohort”.

*A major concern is the low number of animals used in some of the assays. Please address this point as also suggested by the statistic editor.*

When we commenced the study, a larger number of mice was assigned to each group, however, the unexpectedly high level of mortality of *APP* mice significantly reduced the number available for analysis. This is a limitation of our study that has now been clearly articulated in the statistical analyses section of the methods (page 10):

“Power analyses to determine sample size were not carried out a priori for the experiments included in this paper. Based on our previous experience with the techniques being applied, we aimed for sample sizes large enough to reliably detect a moderate effect size. For example, we aimed for a minimum of n=12 mice per genotype per timepoint for behavioural analyses and n=4 mice per genotype per timepoint for histological analyses. The large number of APP mice that died unexpectedly (Figure 1) limited the number of mice available for our experimental analyses and for some experiments or timepoints we were only able to obtain n=3 mice in a group. These small sample sizes are a limitation to our study, as we do not have sufficient power to detect differences with an effect size smaller than 0.5 (Sullivan & Feinn, 2012). However, we consistently show large differences between WT and APP group means (effect sizes >1.0) and post-hoc power analyses revealed that most comparisons had a statistical power >80% (G*Power 3.1; RRID: SCR_013726), with the exception being our t-test comparison of average hippocampal node length per mouse (n=3 mice per group), which only achieved a power of 58%.”.

*This is not a proper form to report the number of animals "n ≥ 3 mice per genotype", please add the number of animals/group in the figure legends/each experiment.*

This has been amended. Each figure legend now includes the number of mice analysed per group.

*Total numbers of animals used is not included.*

In the ‘Animals’ section of the methods text (page 4), we now indicate that:

“Male and female mice were used for these experiments; n=238 mice were assigned to experimental cohorts, including n=29 APP mice that were assigned but died prior to the required timepoint, preventing analysis.”

*Figure 3, The low magnification image (panels A and B) should include the fimbria. Figure 5, 7 and 8, low magnification images (similar to supplementary figure 1) of the slices used to acquire the higher magnifications should be included. The areas of the high magnification images should be indicated in these.*

We now include new low magnification image panels in Figures 7 and 8 as requested. We did not modify the hippocampal panel in Figure 3 to include the fimbria, as low magnification PDGFRα staining in the hippocampus and fimbria is now included in Figure 7.

Indicating the location of each high magnification image on the low magnification images would not be appropriate, as they are not cropped from the low magnification images. Low magnification images were collected for cell quantification. High magnification images were collected from anywhere within the defined anatomical region to show the cellular morphology in small figure panels but were not attributed to specific subfields. On page 9 of the methods we now indicate that:

“High magnification (40x air objective) images were collected from any region of the hippocampus, fimbria and entorhinal cortex and only serve to demonstrate the morphology of the labelled cells quantified.” We have also modified the figure legend text to ensure that it is clear the panels only serve to show cells labelled with each antibody within a given region.

For Figure 5, high magnification (100x oil lens) confocal images were collected to make node and paranode measurements. As these structures can only be seen at high magnification, low magnification images were not collected and cannot be included. In the methods on page 9, we now say:

“To measure node and paranode lengths, Hoescht 33342 nuclear labelling was initially used to identify the CA1 region of the hippocampus or the fimbria, and to ensure the unbiased selection of non-overlapping fields of view within each structure. Images were collected using an UltraView Nikon Ti spinning disk confocal microscope (100x oil objective; 0.5 µm z-spacing).”

**Additional Editorial Comments:**

*The authors should state whether animals from both sexes were used.*

On page 4 of the manuscript, we now indicate that:

”Male and female mice were used for these experiments; n=238 mice were assigned to experimental cohorts, including n=29 APP mice that were assigned but died prior to the required timepoint, preventing analysis.”

*Please move the Acknowledgements and authors contribution before the references.*

We have moved the acknowledgements and author contributions to precede the references and have adjusted the contributions to follow the CRediT Taxonomy.

*Please add to your paper (after the Discussion and Acknowledgments, immediately before the References) a conflict of interest statement*

We now include:

“Conflict of Interest: The authors have no conflict of interest to declare.”

*Please incorporate Research Resource Identifiers (RRIDs) in your citation of all resources used in your manuscript. Please also be sure these RRIDs are included in your keywords list in addition to the required keywords.*

We have added RRID’s to the text and have added them to the keywords. We now include an antibodies table (attached at the end of the manuscript). All antibodies used in this study are commercially available and well validated, and the appropriate citations are provided in the antibody table.

**Statistics Editor: McArthur, David**

*Use of repeated measures one-way or two-way anovas.*

We did not use repeated measures ANOVAs to analyse our behavioural data, as each time point corresponded to a distinct cohort of mice (see response to editor).

*For those studies with n=3 (or quizzically in some instances "n>3"), please be aware that with quite small sample sizes, whatever findings are reported must be interpreted with great caution. Kindly discuss this limitation in the narrative…. Assumption of a normal distribution for those data (n = 3) is unwarranted… There is no evidence of power analysis. On the mandated Questionnaire (not provided) item 3a asks that a sample size rationale be provided.*

We have updated the statistics section of the methods to indicate that the low numbers of mice analysed is a major limitation of this study and included a statement relating to power analysis (Page 10; see response to editor). We have now amended our statement regarding assumption of normal distribution for data with n=3. The statement on Page 10 now reads:

“For data sets with n=3 in any group, we were unable to test for normality, however, we applied parametric tests to analyse these data as the non-parametric equivalents rely on ranking and are unreliable for small sample sizes (GraphPad Prism 8.0).”

*Why are some of your data denoted with SDs but others with SEMs? The use of SEM is not provided with a rationale. SD's are preferred.*

We now have changed all instances of SEM to SD.

*This journal requests that p-values be shown using a consistent decimal exactness:*

All p values are now expressed to 3 decimal points with anything smaller being expressed as p<0.001.

*When samples are small, kindly use dotplots, else use boxplots or violin plots with or without dotplot overlays.*

We have now deleted the bars from the figures to only show the dot plots.

*The captions for Figures 2 shows "as the target zone. p)" but that "p" may be a typo.*

p) has now been corrected in the Figure 2 legend to i).

*Figure 6d is unacceptable if any of the symbols are completely overwritten by other symbols. In this instance you'll want to explore density plots, for which a variety of options are available.*

This is now Figure 6f, and is presented as a relative frequency distribution plot that more clearly shows the shift towards smaller g-ratios for myelinated axons in the *APP* mice.

**Reviewer: 1**

*The discussion (although well written) is too long.*

We now include new discussion points requested by the reviewers but have reduced the overall length of the discussion substantially.

*Can the authors present evidence indicating whether or not APP is expressed exclusively in neurons in this model, or also in glial cells (oligodendrocytes)?*

We performed immunohistochemistry to detect human APP (6E10 antibody) and show that it is expressed in neurons and mature oligodendrocytes, but not OPCs, in *APP* mice (new Figure S1).

*On p.120 the authors propose that oligodendrocyte turnover is higher in APP transgenic mice by P180. Why not P120, as figure 7 clearly shows higher OPC conversion to oligodendrocytes at P120 in the APP transgenic mice?*

In Figure 7, *APP* transgenic mice have a larger number of new oligodendrocytes in the hippocampus, fimbria and entorhinal cortex at P60+120. These mice are P180, as the mice received Tamoxifen at P60 and were analysed 120 days later. We defined this nomenclature in the methods but have also clarified this on page 14 of the results text:

“Coronal brain cryosections from P60+7 (P67), P60+60 (P120), P60+90 (P150) and P60+120 (P180) control and APP transgenic mice … ”.

And in the figure legend:

“P60 control and *APP* mice received tamoxifen at P60, inducing recombination of the Cre-sensitive YFP reporter, effectively labelling OPCs and their progeny. Immunohistochemistry was performed on brain cryosections from mice at P60+7 (P67), P60+60 (P120), P60+90 (P150) and P60+120 (P180).”

*Can the authors quantify the proportion of mature oligodendrocytes (ASPA positive) that are YFP-positive vs YFP-negative at P120? Would this indicate whether there is higher oligodendrocyte turnover in the APP transgenic mice?*

We performed immunohistochemistry to detect YFP, PDGFRα and ASPA in the hippocampus and fimbria, and quantify the density of newborn oligodendrocytes (YFP^+^ PDGFRα-neg ASPA^+^ cells) at P60+120 (P180 mice). These data comprise a new Figure S3 and support the increased turnover of mature oligodendrocytes in *APP* mice.

*It is assumed that the effects seen are attributed to the mutant form of APP and, by extension, amyloid beta, rather than APP overexpression itself…Do the authors have any indication or measure for amyloid beta production prior to P180? Are the behavioural changes driven by amyloid generation or APP overexpression?*

The mice do not overexpress mouse APP, but they do overexpress a known pathological variant of human APP. These mice cannot be used to study the impact of APP overexpression alone, as Aβ-oligomers (~12 kDa band) can be detected in *APP* mice from P30 (Figure 1), even though amyloid plaques are not detected until P180. We now include an extra panel showing Aβ-oligomers in Figure 1 and indicate that any phenotype could be the result of result of elevated human APP or Aβ-oligomers.

Minor comments:

(5) The authors should read through their methods section again and add spacing between the numeral and the unit of measure where this has not been done, e.g. should be 2 mm, not 2mm (p.6); 300 mg, not 300mg (p.5).

We have gone through the text to ensure there is consistently a space after numerals and their unit of measurements.

(6) Show the position of molecular weight markers on Figure 1a.

Fig 1a now includes the molecular weight markers.

(7) Correct the sentence on p.10, “formation of amyloid plaque formation”.

This has been corrected.

(8) First sentence of second paragraph on p. 11 needs correcting “…imbalance in the brain, we next used whole cell…”

This has been corrected.

(9) Explain more clearly in the methods how the % error in the T-maze is calculated. Is this the % for same arm entries/total arm entries?

On page 6 of the methods we now include the formulae for calculating T-maze errors:

“The number of trials in which the mouse failed to alternate was recorded and is expressed as a proportion (%) of the number of trials [(errors / total trial number) x100]”.

**Reviewer: 2**

*The authors should indicate in the manuscript that presenilin not only mediates the cleavage of APP but also of Notch receptors to enable signalling by their cytoplasmic domains. It is important to mention this because Notch signalling pathway controls oligodendrocyte maturation and myelination of axons. This would highlight the pertinence of studying a model without PSEN1 mutation.*

We have altered the introduction on page 3:

“The influence of amyloid pathology on OPC and oligodendrocyte health has also been examined in vivo, in mice carrying human pathological variants of APP and PSEN1 (Desai et al., 2011). …. However, in these mice, it is difficult to attribute specific changes in cells of the oligodendrocyte lineage with APP overexpression or amyloidosis, as PSEN1 modulates Notch signalling (Newman et al., 2014), and can directly impact oligodendrocyte maturation and myelination (Zhang et al., 2009).”

*Could the authors determine in OPCs whether the expression level of GABAA receptor subunits or other related channels are modify in APP mice that may explain the increased response to GABA.*

The change in the response to GABA could be due to a change in the expression of GABA_A_ receptor subunits, or a change in the function of GABA_A_ receptors. As the membrane resistance and resting membrane potential of OPCs was normal in *APP* mice, our data do not point to other ion channel changes that may modulate the magnitude of the current through GABA_A_ receptors. Additionally, OPCs comprise ~5% of brain cells, such that any change in their expression of GABA_A_ receptor subunits or other ion channels would not be detected in hippocampal protein lysates and the poor quality of GABA_A_ receptor subunit immunohistochemistry prevents the single cell quantification of GABA_A_ receptor subunit expression (for example <https://www.eneuro.org/content/6/4/ENEURO.0496-18.2019>). We now include data showing that human APP is expressed by neurons and oligodendrocytes but not OPCs (new Figure. S1) and hypothesise that exogenous human APP alters OPC GABA_A_ receptor expression or function. We have updated the discussion to include this idea.

*The authors should evaluate the potential effects on neurons of myelin thickness increased and modification of node of Ranvier. Are the number of neurons, the synaptic density modified?*

Myelination is impacted by neuron and oligodendrocyte signalling, and OPCs and oligodendrocytes influence neurotransmitter release and synaptic efficacy (reviewed Pepper et al., 2018). This makes it very difficult to determine whether neuronal or glial changes are responsible for our phenotype. However, human APP is expressed in oligodendrocytes (Figure S1) and our TEM data indicate that axon density is normal in *APP* mice at P120 (when paranode length and myelin thickness are increased), suggesting that projection neuron number is normal. Furthermore, the diameter of myelinated axons is also normal in *APP* mice, suggesting that altered axon size is not responsible for the increased myelin thickness. These points are now highlighted in the results text.

The text on page 14 now reads:

“We found that axon density (Fig. 6c), myelinated axon density (Fig. 6d), and the proportion of axons that are myelinated (Fig. 6e) was equivalent between WT and APP mice, suggesting that axon number and the proportion of axons that get myelinated during development is not affected by genotype. However, the g-ratio [axon diameter / (axon + myelin diameter)] of myelinated axons in the hippocampus of APP mice was reduced relative to WT mice (Fig. 6f-g), suggesting that APP mice have thicker hippocampal myelin. This was confirmed when we measured the diameter of the myelinated axons (Fig. 6h-l), this was equivalent in WT and APP mice, confirming that the physical size of the axons was not driving the change in myelin thickness, but found that the number of myelin lamellae (wraps) surrounding each axon was increased in APP mice (Fig. 6m).”

*The discussion on the expression of the human APP transgene is too long in relation to the main topic of the study. Also, the discussion related to behavioral study is off topic. It is difficult to compare behavioural experiments that use different paradigms and mouse strains. The authors should rather discuss their behavioral results in the context of myelin and oligodendrocyte impairments they observed; and how these impairments may explain or not the anxiety and memory phenotypes. What are the potential physiological effects of decreased Node of Ranvier and increased paranode length in APP mice ?*

The discussion has now been substantially shortened.

*I do not understand the hypothesis that “the increased myelin thickness could result from an upregulation in neuregulin-1 type III expression”. Could the authors clarify this point since there is no mutation of BACE1 in this transgenic model ?*

This was removed when we shortened the discussion.

**Reviewer: 3**

Main comments

*1. anti-ASPA was used in Figure 8 to analyse the total population of OL. The abbreviation for ASPA was not defined in the Methods section.*

We have now defined ASPA and details of the antibody are included in the antibody table.

*According to the papers listed below aspartoacylase is also detected in microglia throughout the brain. A count of ASPA+ cells might therefore be expected to include both microglia and differentiated OL.*

We performed immunohistochemistry to detect ASPA^+^ and the microglial marker Iba1 (new Figure S5). ~2% of ASPA^+^ cells were closely apposed with Iba1^+^ cell bodies, however, no microglia expressed ASPA. It should be noted that we did not use the same antibody used by Moffett et al., (2011), which showed strong ASPA labelling in morphologically defined microglia in rat tissue.

*2. Relating to point 1, if amyloid pathology involves increased turnover of OL it should be possible to detect an increase in OL cell death. This could be examined by histological analysis of apoptosis/necroptosis in APP and control sections at P180, or by examining chromatin condensation in existing EM micrographs.*

The TEM micrographs were collected at P120. This precedes the significant increase in new oligodendrocyte number detected from P150 in the hippocampus and P180 in the fimbria and entorhinal cortex and cannot be used to quantify oligodendrocyte death. New data in Figure S1 indicate that oligodendrocytes express human APP in APP transgenic mice. New data presented in Figure S3 allowed us to determine that ~11% of all ASPA^+^ mature oligodendrocytes are newborn oligodendrocytes in the hippocampus of P180 control mice, compared with ~26% of all ASPA^+^ mature oligodendrocytes in the hippocampus of P180 *APP* mice. Similarly, ~5% of mature oligodendrocytes in the fimbria of control mice are newborn compared with ~13% in APP mice. We were unable to obtain TUNEL labelling prior to the Covid-19 Institute closure and are therefore unable to conclude that oligodendrocytes die. We have therefore been careful not to claim that oligodendrocyte turnover does occur – and only state that our data may be explained by an increase in oligodendrocyte turnover.

*3. The authors should explain more clearly in the introduction (last paragraph) the rationale for focusing solely on changes in the hippocampus… Knowledge on whether APP mice exhibit similar changes to OPC and myelin in cortical grey matter regions associated with spatial memory, e.g. retrosplenial cortex, would be of great interest to the field.*

We have edited page 4 to clarify our choice of regions / mouse type:

” We aimed to determine whether pathological APP signalling, in the absence of pathological PSEN1 or MAPT, was sufficient to influence oligodendrocyte lineage cell function in the hippocampus, fimbria or entorhinal cortex in early stages of disease. The hippocampus and entorhinal cortex are brain regions affected early in Alzheimer’s disease (Du et al., 2001; Pennanen et al., 2004), and the fimbria is the major white matter tract that connects the hippocampal hemispheres to subcortical and cortical regions such as the thalamus and prefrontal cortex (Jin & Maren, 2015; Wyss, Swanson, & Cowan, 1980).”

At the reviewer’s request, we have also quantified oligodendrocyte addition in the retrosplenial cortex and include these data as new Figure S4.

*Do the increased currents observed with bath applied GABA translate into measurable changes in the properties of GABAergic synaptic inputs? … Although additional patch-clamping experiments would be beyond the scope of the present work, the authors should comment directly on the potential for changes induced through amyloid pathology to directly influence inhibitory interneuron-OPC signalling.*

Neurons can communicate with OPCs via both synaptic and non-synaptic mechanisms. While our experiments examined “post synaptic” changes (ie changes in OPCs), we do not know how pathological human APP may influence the “pre-synaptic” neurons to modulate neuron-OPC communication. As APP modulates presynaptic GABA_B_-receptor signalling, we have revised our discussion to include the idea that APP could modulate presynaptic neuron-OPC signalling.

5. *It would be interesting to know if these newly generated OL contribute to myelination.*

Unfortunately, YFP labelling does not extend into the myelin sheath, which prevents co-labelling with myelin markers such as MBP or PLP. However, we now include new data in Figure S3 demonstrating that most of the adult-born oligodendrocytes express ASPA and are therefore mature oligodendrocytes.

6. *Changes in cognitive function are detected by P90 in the T maze task (more repeat entries in the APP mice) while changes in OPC function and myelin structure appear soon after at P100. The timing of these events is interesting. Although the authors acknowledge that impairments in the T maze changes may stem from the hyperactivity characterised in these mice (page 14), working memory in the T maze task is also hippocampus-dependent, thus it would be helpful for the authors to comment on the potential link between working memory impairment and alterations in OPC and OL function.*

In our discussion on page 17, we have included the following text:

“Altered OPC and/or oligodendrocyte function could also contribute to behavioural abnormalities in APP mice, as both cell types influence neural circuit function, memory and affective behaviour (reviewed Pepper, Pitman, Cullen, & Young, 2018). The ablation of OPCs has been shown to produce anxiety-like behaviour within 7 days (Birey et al., 2015), and while the electrophysiological response of OPC is altered by P100 in APP mice, any reduction detected in OPC density is minor, and APP mice do not display an anxiety-like phenotype until P180. Similarly, increased hippocampal myelin thickness and the onset of working memory deficits are detected early in APP mice, however, in other contexts, thick myelin enhances hippocampal-dependent memory formation (Jeffries et al., 2016), making it unlikely that this cellular change impairs working memory performance, but this cannot be entirely ruled out within this pathological context.”

Minor comments

Methods
1. Catalogue numbers should be included for the antibodies.

The catalogue numbers are now included in the antibody table and RRIDs for all antibodies are included in the text and in the table.

2. Why assume a normal distribution for N=3 experiments? (page 9).

The statistical analysis text in the methods has been updated to clarify this point:

“For data sets with n=3 in any group, we were unable to test for normality, however, we applied parametric tests to analyse these data as the non-parametric equivalents rely on ranking and are unreliable for small sample sizes (GraphPad Prism 8.0).”

3. Quantification of nodes / paranodes needs a little more description, e.g. how many fields / section, how many sections per animal?

The confocal microscopy methods section has now been updated to give more details about node/paranode analysis:

“To measure node and paranode lengths, Hoescht 33342 nuclear labelling was initially used to identify the CA1 region of the hippocampus or the fimbria, and to ensure the unbiased selection of non-overlapping fields of view within each structure. Images were collected using an UltraView Nikon Ti spinning disk confocal microscope (100x oil objective; 0.5 µm z-spacing). When nodes and their flanking paranodes were intact within a single z-plane, they were measured manually using Fiji software (NIH, Washington DC, USA). For the CA1 region of the hippocampus, 30-90 nodes and paranodes were measured, sampled from 4 fields of view per mouse. For the fimbria, 36-94 nodes and paranodes were measured, sampled from 2 field of view per mouse. All measurements were made by a researcher blind to genotype.”

4. Ki67 is mentioned in the list of antibodies, but has not been used, please clarify.

This was an error and has now been deleted from the text.

5. Cortical used twice in same sentence (page 17, 2nd paragraph).

The discussion has been revised substantially and this double use of cortical has been removed.

6. Labelling of Y axis in Figure 7 p q and r is confusing, it mentions PDGRa- YFP^+^ cells, but legend describes the axis as YFP+ olig2+ PDGFRa -ve cells. Please clarify.

We have corrected this discrepancy.
